# Palladium‐Catalyzed Non‐Directed C─H Functionalization of Arenes with Trifluoromethylated Olefins

**DOI:** 10.1002/chem.202501973

**Published:** 2025-07-23

**Authors:** Levin Stockmann, Claire Empel

**Affiliations:** ^1^ Institute of Organic Chemistry RWTH Aachen University Landoltweg 1 52074 Aachen Germany; ^2^ University of Bayreuth Universitätsstraße 30 95447 Bayreuth Germany

**Keywords:** C─H Functionalization, fluorine chemistry, Pd‐catalysis, trifluoro methylated olefines

## Abstract

Herein, we report the palladium‐catalyzed non‐directed C─H functionalization reaction of simple arenes with α‐trifluoromethyl styrene derivatives. The application of 2‐pyridone ligands increased the yield and selectivity of the alkenylation reaction and enabled a broad scope of different simple arenes and α‐trifluoromethyl styrenes (25 examples, up to 94% yield). Overall, a robust reaction is developed as described by a sensitivity screening. Detailed experimental studies on the reaction were performed to further understand the mechanism. Palladium‐catalyzed non‐directed C─H alkenylation of simple arenes with α‐trifluoromethyl styrene derivatives is accomplished using 2‐pyridone ligands, which significantly enhances both yield and selectivity. This approach enables efficient functionalization across a wide range of arenes and styrene substrates. Mechanistic studies further clarify the reaction pathway.

## Graphical abstract

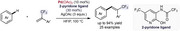

## 1 Introduction

The selective activation of C─H bonds still belongs to one of the most challenging approaches in organic chemistry.^[^ ^1^ ^]^ In general, two distinct approaches find regular application. The first approach involves directing group‐mediated C─H activation to enable the selective activation of one specific C─H bond. The advantage of this concept clearly lies within the high control over the C─H activation. However, the requirement of a directing group impacts on the applicability of such reactions.^[^ ^2^ ^]^ The second approach describes a non‐directed C─H activation. In this case, the selectivity of the C─H activation step is influenced by the design of the ligand system. The advantage of such non‐directed C─H activations is that simple arenes can be used without any pre‐functionalization or installation of directing groups.^[^ ^3^ ^]^

**Scheme 1:**
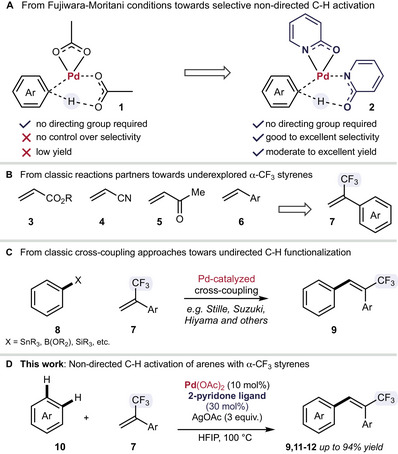
**A** From classic Fujiwara‐Moritani conditions toward non‐directed C─H activation; **B** α‐trifluoromethyl styrene derivatives as reaction partner; **C** Classic cross‐coupling reactions for the synthesis of 3,3,3‐trifluoroprop‐1‐ene‐1,2‐diyl)dibenzene; **D** Non‐directed C─H activation with α‐trifluoromethyl styrene derivatives.

Over the past years, 2‐pyridone ligands were recognized as a powerful handle to accelerate palladium‐catalyzed C─H activation reactions (Scheme 1A).^[^ ^4^ ^]^ 2‐Pyridone ligands or systems based on electron‐poor *N*‐heterocyclic ligands in combination with *N*‐acetylglycine and derivatives thereof (dual ligand approach)^[^ ^5^ ^]^ and others^[^ ^6^ ^]^ build an extremely powerful tool to fine‐tune the electronic properties of the Pd‐complex enabling selective C─H activation reactions. Applications of such non‐directed palladium‐catalyzed C─H functionalization reactions include cyanation,^[^ ^7^ ^]^ iodination,^[^ ^8^ ^]^ deuteration,^[^ ^9^ ^]^ alkynylation,^[^ ^10^ ^]^ or olefination^[^ ^11^ ^]^ reactions.

However, such olefination reactions or Fujiwara‐Moritani‐type reactions are limited in substrate scope and mainly focus on the application of electron.poor olefines such as acrylates **3** as well as nitriles **4**, ketones **5**, amides, sulfonamides and phosponates are used.^[^ ^12^ ^]^

Commonly used methods for the synthesis of multiple‐substituted olefins, such as (3,3,3‐trifluoroprop‐1‐ene‐1,2‐diyl)dibenzene, rely on classic approaches such as the Wittig reaction^[^ ^13^ ^]^ or cross‐coupling reactions;, e.g., via Stille,^[^ ^14^ ^]^ Suzuki,^[^ ^15^ ^]^ Hiyama,^[^ ^16^ ^]^ and other^[^ ^17^ ^]^ cross‐coupling reactions (Scheme 1C).

To overcome the limitation of mono‐substituted olefines in non‐directed C─H functionalization reactions we envisioned to study α‐alkyl styrene derivatives. Especially α‐trifluoromethyl styrene derivatives caught our attention, as the trifluoromethyl group manipulates the electronic properties and displays a suitable reaction partner. Additionally, the importance of fluorine in organic chemistry,^[^ ^18^ ^]^ indicates great potential of α‐trifluoromethyl styrene derivatives as reaction partners in Fujiwara‐Moritani reactions (Scheme 1D).

## 2 Results and Discussion

**Scheme 2:**
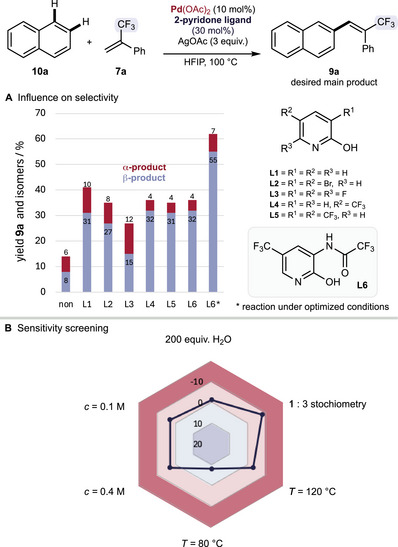
**A** Influence of 2‐pyridon ligands on the selectivity; **B** sensitivity screening.

In a first step, we investigated different 2‐pyridone ligands in the non‐directed C─H functionalization reaction of naphthalene **8a** with α‐trifluoromethyl styrene **7a**. This model reaction was selected because it can yield two distinct regioisomers, resulting from C─H activation at either the α‐ or β‐position. Furthermore, *E*‐ and *Z*‐isomers can be formed in the reaction, resulting in a total of four different potential reaction products. Initial results in the absence of any ligand delivered a non‐selective, low‐yielding reaction outcome, which we considered an ideal starting point to study the influence of 2‐pyridone ligands on the selectivity. In general, the addition of 2‐pyridone ligands improved the reaction yield significantly. Moreover, we identified a trifluorometyl group in the 5‐position of the 2‐pyridone skeleton to be crucial to improve the regioselectivity of the reaction. The importance of the electron‐withdrawing substituent in the 5‐position can be rationalized by its influence on the keto–enol tautomerism, shifting the equilibrium toward the keto form, which is more favored for coordination.^[^ ^19^ ^]^ As a result, the coordination occurs faster and more efficiently, suppressing the less selective and less efficient reaction catalyzed by Pd(OAc)_2_. With these results in hand, we further investigated 5‐(trifluoromethyl)pyridin‐2(1*H*)‐one derivatives and identified ligand **L6** as a suitable candidate for further reaction optimization (Scheme 1C). After further optimization of reaction parameters such as palladium source, oxidant, stoichiometry and reaction temperature, we were delighted to obtain the desired β‐functionalized naphthalene product – (*E*)‐2‐(3,3,3‐trifluoro‐2‐phenylprop‐1‐en‐1‐yl)naphthalene) **9a** – in good yield and regioselectivity (Scheme 1C and ESI Tables S1–S6). Moreover, the present reaction is very robust and changes in concentration, stoichiometry or temperature only slightly impact on the reaction yield. Notably, a reduced reaction temperature leads to a higher formation of the undesired α‐functionalized naphthalene (Scheme 2B).

**Scheme 3:**
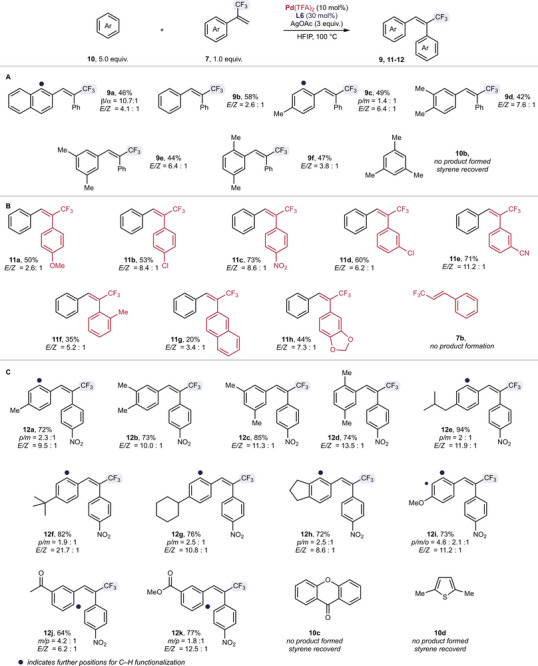
Scope of different arenes in non‐directed C─H functionalization reaction with α‐trifluoromethyl styrene derivatives yields refer to isolated E‐isomers.

Next, we investigated the scope of this non‐directed C─H functionalization using different simple arenes in the reaction with α‐trifluoromethyl styrene **7**. Overall, moderate to good yields of the desired *E*‐isomers were isolated (**9a**‐**f**). In the case of toluene as reaction partner, two regioisomers were obtained with a regioselectivity of 1.4: 1. As expected, different xylenes gave only one regioisomer, while sterically more demanding mesitylene did not give the desired reaction product and styrene **7a** remained untouched in the reaction mixture. Notably, the *E‐* to *Z*‐selectivity increased significantly when using xylene derivatives (Scheme 3A).

To further broaden the applications of this non‐directed C─H functionalization, we next studied different α‐trifluoromethyl styrenes in the reaction with benzene. To our delight, a variety of different α‐trifluoromethyl styrenes were compatible with the reaction protocol and isolated yields for the *E‐*products range from 20–73% (**11a**‐**h**). Additionally, we noticed, that the *E‐* to *Z*‐selectivity strongly depends on the α‐trifluoromethyl styrene reaction partner – electron‐withdrawing substituents (e.g., nitro‐ or cyano‐groups) resulted in the preferred formation of the desired *E*‐isomer (**11c**, **11e**). Based on this, isolated yields for electron‐withdrawing substituents are higher as isolated yields refer exclusively the desired *E*‐isomer. When investigating (*E*‐3,3,3‐trifluoroprop‐1‐en‐1‐yl)benzene **7b** under the optimized reaction conditions in the reaction with benzene, we did not observe any product (Scheme 3B).

In a last step, we studied different arenes in the reaction with 1‐nitro‐4‐(3,3,3‐trifluoroprop‐1‐en‐2‐yl)benzene **7c**, which was chosen to selectively study the regioselectivity for different arenes as the *E‐* to *Z*‐selectivity is expected to be high. A broad variety of different simple arenes was compatible and the desired products were isolated in good to excellent yield, low regioselectivity and good to excellent *E‐* to *Z*‐selectivity (**12a**‐**h**).

Simple functional groups like ethers, ketones, and esters were well tolerated, and the corresponding products were isolated in good yield (**12i**‐**k**). In most cases, only the C─H functionalizations of the *para*‐ and the *meta*‐position were observed, only in the case of anisole, the *ortho*‐position got activated and resulted in the formation of the *ortho*‐functionalized product as the minor product. Unfortunately, heterocycles such as 9*H*‐xanthen‐9‐one **10c** and 2,5‐dimethylthiophene **10d** were incompatible with our reaction protocol and styrene **7c** remained unreacted in the reaction mixture. For 9*H*‐xanthen‐9‐one **10c** the larger highly conjugated π‐system and the lower ability toward palladation can explain the incompatibility. For 2,5‐dimethylthiophene **10d**, however, the poisoning of the Pd‐catalyst seems to be reasonable (Scheme 3C).

**Scheme 4:**
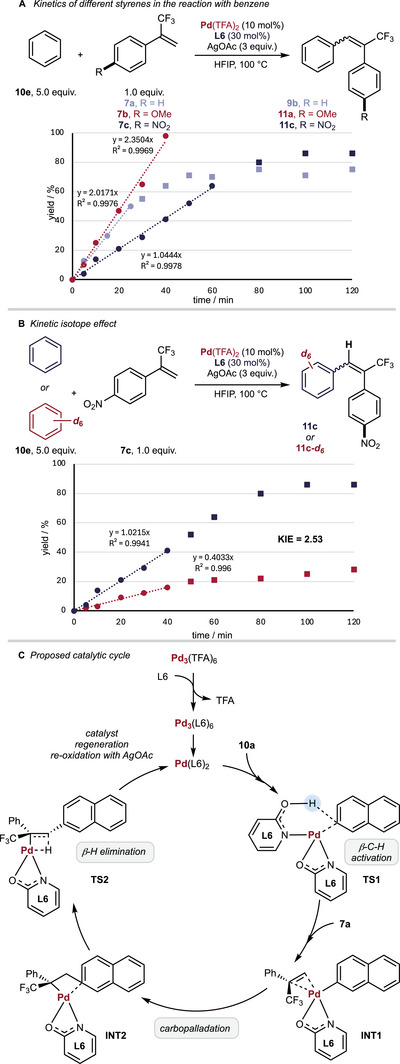
**A** Investigation of reaction kinetics of different α‐trifluoromethyl styrene derivatives; **B** Investigation of kinetic isotope effect; **C** Proposed catalytic cycle. ***Combined yields of E‐ and Z‐product are presented***.

To further understand the influence of different substrates on our Pd‐catalyzed, non‐directed C─H functionalization reactions, we investigated the reaction kinetics of electron‐rich, electron‐neutral, and electron‐poor α‐trifluoromethyl styrenes. While electron‐rich and electron‐neutral α‐trifluoromethyl styrenes reacted similarly fast, the product formation in the reaction with 1‐nitro‐4‐(3,3,3‐trifluoroprop‐1‐en‐2‐yl)benzene **7c** was significantly slowed down. However, a slower reaction seems to result in a higher *E‐* to *Z*‐selectivity (Scheme 4A and Figures S3–S5). Next, we investigated the kinetic isotope effect (KIE) of our reaction.

When performing the reaction of deuterated benzene **8e** with 1‐nitro‐4‐(3,3,3‐trifluoroprop‐1‐en‐2‐yl)benzene **7c** we observed a significantly reduced rate of product formation and calculated the KIE to be 2.53, which indicates the C─H activation step to be rate‐determining (Scheme 4B).

Based on our experimental mechanistic investigations and literature precedence,^[^ ^20^ ^]^ we propose the trimeric palladium complex first dissociates to a palladium monomer, followed by the coordination of **8a**. Next the selectivity determining C─H activation takes place via **TS1** to give **INT1** upon coordination of **7a**. Interestingly, reactions in the presence of *N*‐(2‐hydroxy‐5‐(trifluoromethyl)pyridin‐3‐yl)acetamide as ligand β/α‐selectivity decreased significantly, pointing at the coordination via the 2‐pyridon functional group (for details please see ESI Table S1, entry 8).^[^ ^[Link]^, ^[Link]^ ^]^

Next, the C─C bond forming takes place to furnish **INT2** via carbopalladation. Following, the β‐hydride elimination takes place to give the *E*‐configurated reaction product as the main product. In a last step, the re‐oxidation of the intermediately‐formed Pd^0^ species takes place in the presence of AgOAc to regenerate Pd^II^(L6)_2_ (Scheme 4C). The preferred formation of the *E‐*configurated product can be reasoned by a possible π‐π‐stacking of the two aromatic rings in the β‐H elimination step. At the same time, this geometry prevents the repulsion of the π‐electrons of the naphthalene and the CF_3_‐group of **7a**.

## 3 Conclusion

In summary, we described the non‐directed palladium‐catalyzed C─H activation of simple arenes followed by their functionalization with α‐trifluoromethyl styrene derivatives. Depending on the electronic properties of the α‐trifluoromethyl styrene derivatives high yields and good *E‐* to *Z*‐selectivities can be achieved. Kinetic experiments suggest that at the C─H activation step to be rate determining.

## Supporting Information

Additional references cited within the Supporting Information.^[^ ^16^, ^17^, ^21^ ^]^

## Conflict of Interest

The author declares no conflict of interest.

## Supporting information



Supporting Information

## Data Availability

The data that support the findings of this study are available in the supplementary material of this article.
