# Supplementary material for: Palladium‐Catalyzed Non‐Directed C─H Functionalization of Arenes with Trifluoromethylated Olefins
Source: Chemistry. 2025 Jul 23;31(47):e01973. doi: 10.1002/chem.202501973 (PMC12376254; doi:10.1002/chem.202501973)
Supplement: Supplementary file 1 — Supporting Information [file CHEM-31-e01973-s001.pdf]

Table of Contents

1.	General Information	S1
2.	Reaction Optimization	S2
3.	Sensitivity Screening	S5
4.	General Procedure	S6
5.	Kinetic Studies	S6
6.	Physical Data	S12
7.	NMR Spectra	S26
8.	References	S66

1. General Information

Unless otherwise noted, all commercially available compounds were used as provided without further purification. Chemicals used in this manuscript were purchased from Sigma Aldrich, Alfa Aesar, Chempur, Fluorochem, Activate Scientific and Carl Roth. Solvents used in reactions were p.A. grade. Solvents for chromatography were technical grade and distilled prior to use. Analytical thin-layer chromatography (TLC) was performed on Macherey-Nagel silica gel aluminium plates with F-254 indicator, visualized by irradiation with UV light. Column chromatography was performed using silica gel Merck 60 (particle size 0.063 – 0.2 mm). Solvent mixtures are understood as volume/volume. ^{1}H NMR, ^{19}F NMR and ^{13}C{^{1}H,^{19}F} NMR were recorded on a Bruker 600 MHz NMR spectrometer in $CDCl_3$. Data are reported in the following order: chemical shift (δ) in ppm; multiplicities are indicated brs (broadened singlet), s (singlet), d (doublet), t (triplet), q (quartet), m (multiplet); coupling constants (*J*) are in Hertz (Hz). HRMS data were recorded on a ThermoFisher Scientific LTQ Orbitrap XL using ESI ionization or on a Finnigan MAT 95 using EI ionization at 70 eV. IR spectra were recorded on a Perkin Elmer-100 spectrometer and are reported in terms of frequency of absorption (cm^{-1}).

2. Reaction Optimization

Structural analysis of products was performed based on ^{19}F NMR spectroscopy.[16,17,21]

Table S1: Ligand Optimization.

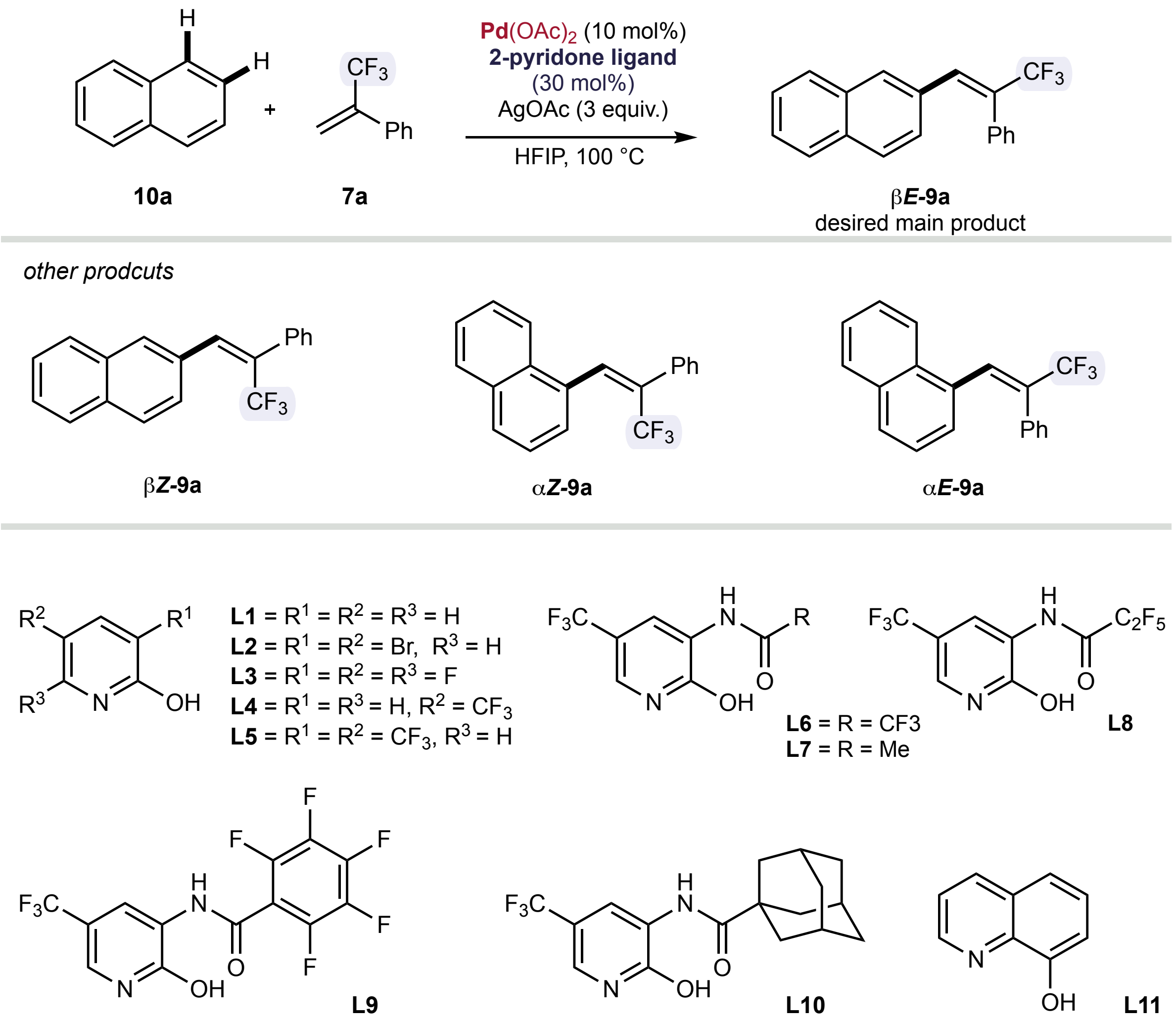

#	Ligand	**α*E*-9a** / %	**α*Z*-9a** / %	***βE*-9a** / %	***βZ*-9a** / %	overall yield / %	*E/Z*	β/α
1	-	3	3	4	4	14	1 : 1	1.3 : 1
2	L1	7	3	24	7	41	3.1 : 1	3.1 : 1
3	L2	5	3	21	6	34	2.9 : 1	3.4 : 1
4	L3	8	4	10	5	27	2 : 1	1.3 : 1
5	L4	3	1	25	6	35	4 : 1	7.7 : 1
6	L5	3	1	21	10	35	2.2 : 1	7.6 : 1
7	L6	3	1	24	8	36	3 : 1	8 : 1
8	L7	3	10	45	13	71	3.4 : 1	4.5 : 1
9	L8	3	1	24	8	36	3 : 1	8 : 1
10	L9	3	1	24	6	34	3.7 : 1	7.5 : 1
11	L10	3	1	24	6	34	3.7 : 1	7.5 :1
12	L11	*no reaction*						

Reaction conditions: Naphtalene (0.2 mmol, 1.0 equiv.), α-CF_3-Styrene (2.0 equiv.), Ligand (30 mol%), $Pd(OAc)_2$ (10 mol%) and AgOAc (3.0 equiv.) in HFIP (1 mL) at 100 °C for 16 h. The yield was determined by ^{19}F-NMR using trifluoromethylbenzene (25 μL*)* as internal standar

Table S2: Palladium source optimization.

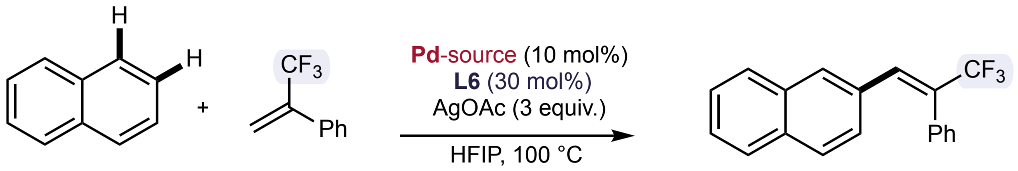

10a, 1.0 equiv. **7a**, 2.0 equiv β***E*-9a** desired main product

#	Pd-source	**α*E*-9a** / %	**α*E*-9a** / %	***βE*-9a** / %	***βZ*-9a** / %	overall yield / %	*E/Z*	β/α
1	$Pd(OAc)_2$	3	2	31	10	46	2.8 : 1	8.2 : 1
2	$Pd(TFA)_2$	5	1	34	12	52	3 : 1	7.7 : 1
3	$Pd_2(dba)_3$	4	1	27	8	39	3.4 : 1	7 : 1
4	$[(Ph)_3P]PdCl_2$	4	1	26	8	39	3.3 : 1	6.4 : 1
5	$PdCl_2$	*no reaction*						

Reaction conditions: Naphtalene (0.2 mmol, 1 equiv.), α-CF_3-Styrene (2.0 equiv.), L6 (30 mol%), Pd-source (10 mol%) and AgOAc (3.0 equiv.) in HFIP (1 mL) at 100 °C for 16 h. The yield was determined by ^{19}F-NMR using trifluoromethylbenzene (25 µL*)* as internal standard.

Table S3: Stoichiometry optimization.

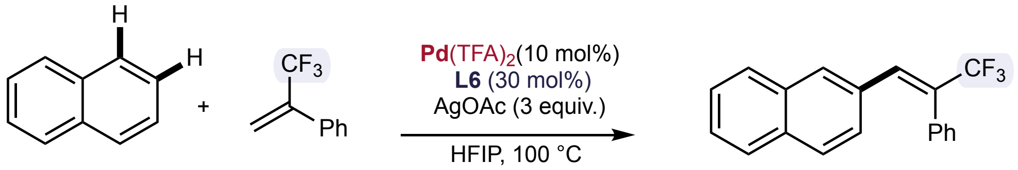

10a **7a** β***E*-9a** desired main product

#	stoichiometry **8a** : **7a**	**α*E*-9a** / %	**α*E*-9a** / %	***βE*-9a** / %	***βZ*-9a** / %	overall yield / %	*E/Z*	β/α
1	1 : 2	5	1	31	14	51	2.4 : 1	7.5 : 1
2	3 : 1	6	1	35	12	54	3.2 : 1	6.7 : 1
3	5 : 1	5	2	41	14	62	2.9 : 1	7.9 : 1

Reaction conditions: Naphtalene, α-CF_3-Styrene, L6 (30 mol%), $Pd(TFA)_2$ (10 mol%) and AgOAc (3.0 equiv.) in HFIP (1 mL) at 100 °C for 16 h. The yield was determined by ^{19}F-NMR using trifluoromethylbenzene (25 µL*)* as internal standard.

Table S4: Oxidant optimization.

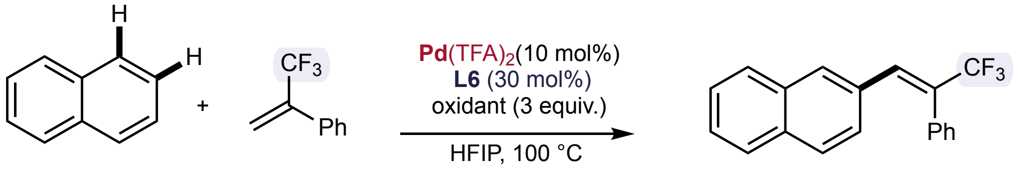

10a, 5.0 equiv. **7a**, 1.0 equiv β***E*-9a** desired main product

#	oxidant	***αE*-9a**/ %	***αE*-9a** / %	***βE*-9a** / %	***βZ*-9a** / %	overall yield / %	*E/Z*	β/α
1	Ag_2CO_3	4	3	38	11	56	3 : 1	7 : 1
2	AgTFA	15	3	27	5	50	5.3 : 1	1.8 : 1
3	$PhI(OAc)_2$	10	2	16	2	30	6.5 : 1	1.5 : 1
4	$Cu(OAc)_2$	11	2	39	5	57	7.1 : 1	3.4 : 1
5	AgF	7	4	19	11	41	1.7 : 1	2.7 : 1
6	$K_2S_2O_8$	18	3	17	2	40	7.0 : 1	0.9 : 1
7	AgOAc	5	2	42	13	62	3.1 : 1	7.9 : 1

Reaction conditions: Naphtalene, α-CF_3-Styrene, L6 (30 mol%), $Pd(TFA)_2$ (10 mol%) and oxidant (3.0 equiv.) in HFIP (1 mL) at 100 °C for 16 h. The yield was determined by ^{19}F-NMR using trifluoromethylbenzene (25 µL*)* as internal standard.

Table S5: Concentration optimization.

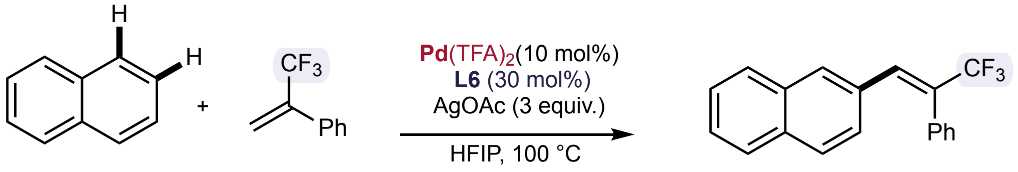

10a, 5.0 equiv. **7a**, 1.0 equiv β***E*-9a** desired main product

#	concentration / M	***αE*-9a** / %	***αE*-9a** / %	***βE*-9a** / %	***βZ*-9a** / %	overall yield / %	*E/Z*	β/α
1	0.4	4	3	41	11	59	3.2 : 1	7.4 : 1
2	0.2	5	2	42	13	62	3.1 : 1	7.9 : 1
3	0.1	5	3	40	11	59	3.2 : 1	6.3 : 1

Reaction conditions: Naphtalene, α-CF_3-Styrene, L6 (30 mol%), $Pd(TFA)_2$ (10 mol%) and oxidant (3 equiv.) in HFIP at 100 °C for 16 h. The yield was determined by ^{19}F-NMR using trifluoromethylbenzene (25 µL*)* as internal standard.

Table S6: Temperature optimization.

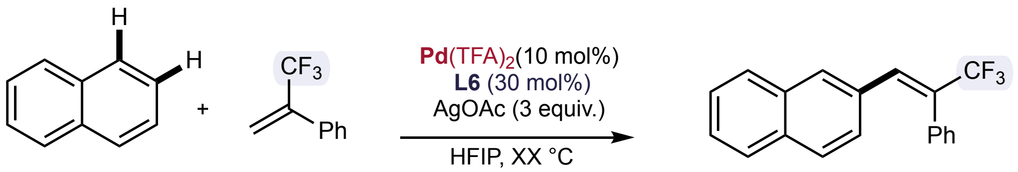

10a, 5.0 equiv. **7a**, 1.0 equiv β***E*-9a** desired main product

#	Temperature / °C	**α*E*-9a** / %	**α*E*-9a** / %	**β*E*-9a** / %	**β*Z*-9a** / %	overall yield / %	*E/Z*	β/α
1	80	10	3	40	17	70	2.5 : 1	4.4 : 1
2	100	5	2	42	13	62	3.1 : 1	7.9 : 1
3	120	6	2	38	13	59	2.9 : 1	6.4 : 1

Reaction conditions: Naphtalene, α-CF_3-Styrene, L6 (30 mol%), Pd(TFA)$_2$ (10 mol%) and oxidant (3.0 equiv.) in HFIP (1 mL) for 16 h. The yield was determined by ^{19}F-NMR using trifluoromethylbenzene (25 µL*)* as internal standard.

3. Sensitivity Screening

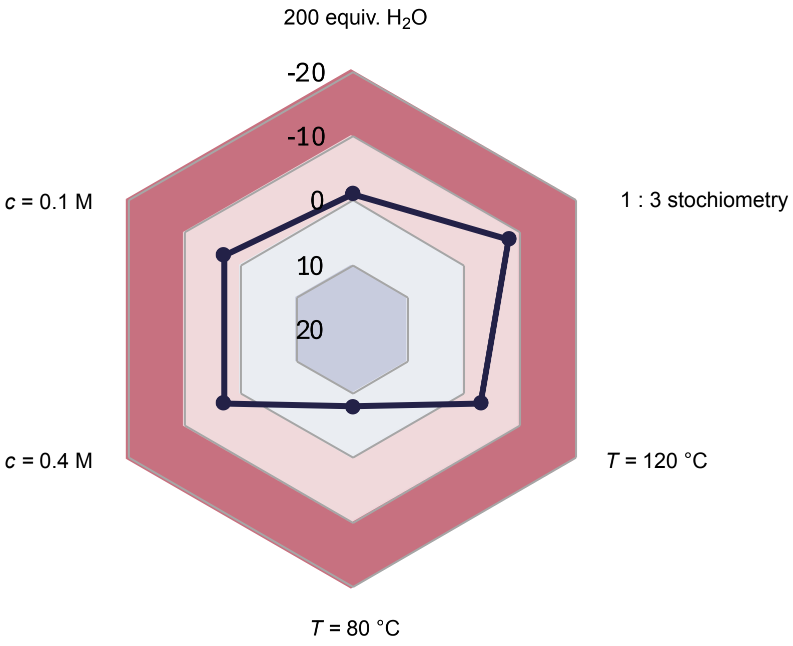

Note: The higher reaction yield at lower reaction temperatures is due to an increase in formation of the undesired α-isomer.

Figure S1: Robustness Screening.

4. General Procedure

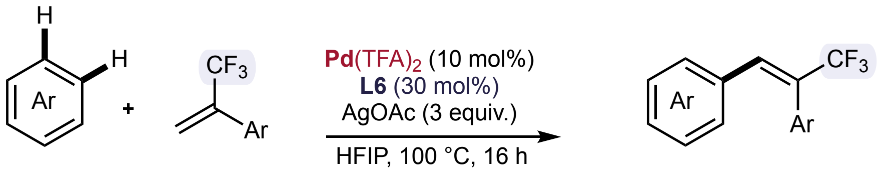

10 **7** **9,11-12**

An oven-dried screw cap tube (total volume 12 mL) was loaded with $Pd(TFA)_2$ (10 mol%), L6 (30 mol%), AgOAc (3 equiv., 0.6 mmol), arene (5.0 equiv., 1.0 mmol) and α-trifluoromethyl styrene (1 equiv., 0.2 mmol), then 1 mL of HFIP was added. The test tube was caped and stirred for 16 h at 100 °C. The desired product was obtained after silica column chromatography using *n*-pentane / Et_2O as eluent.

5. Kinetic Studies

General procedure for kinetic studies

An oven-dried GC-vial (total volume 2 mL) was loaded with $Pd(TFA)_2$ (10 mol%), L6 (30 mol%), AgOAc (3 equiv.), arene (5.0 equiv.) and α-trifluoromethyl styrene (1 equiv., 0.025 mmol), then 0.25 mL of HFIP was added. The GC-vial was caped and stirred for the time indicated at 100 °C. The yield was determined by ^{19}F-NMR using trifluoromethylbenzene (25 µL*)* as internal standard.

Investigation of Kinetic Isotope Effect (KIE)

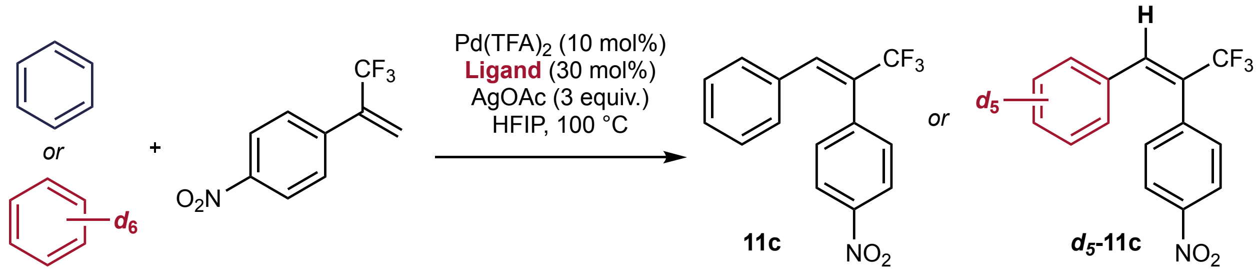

time / min	0	5	10	20	30	40	50	60	80	100	120
yield **10c**	0	4	14	21	29	41	52	64	80	86	86
yield ***d*$_5$-10c**	0	2	3	9	12	16	20	21	22	25	28

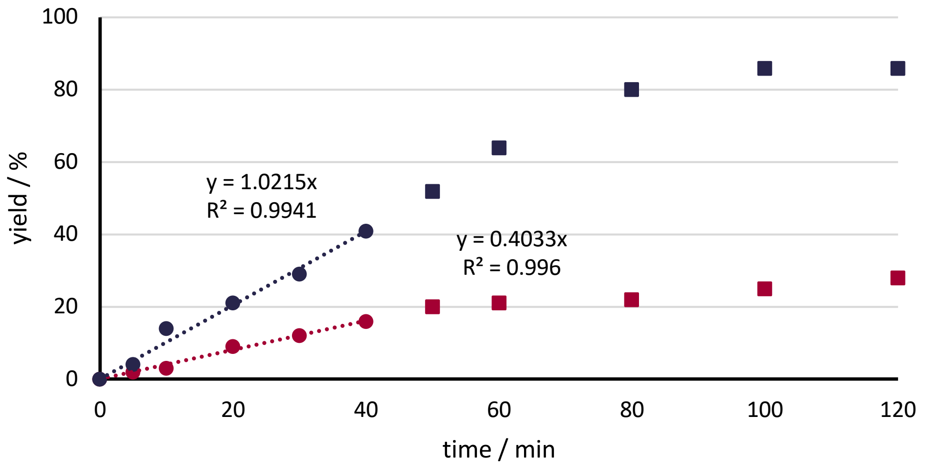

Figure S2: Reaction kinetics for the formation of **11c** and ***d*$_5$-11c**.

(*E*)-1-(3,3,3-trifluoro-2-(4-nitrophenyl)prop-1-en-1-yl)benzene-2,3,4,5,6-*d*5 (*d*5-11c)

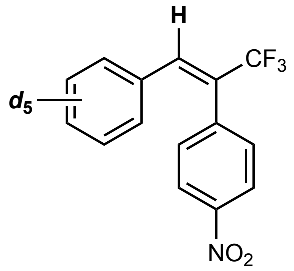

^{1}H NMR (600 MHz, Chloroform-*d*): δ = 8.27 (d, *J* = 8.7 Hz, 2H), 7.52 (d, *J* = 8.5 Hz, 2H), 7.39 (d, *J* = 1.8 Hz, 1H) ppm.
^{19}F NMR (565 MHz, Chloroform-*d*): δ= -65.19 ppm.
HRMS (ESI): *m/z*: $[M]^+$ Calcd. for $C_{15}H_5D_5NO_2F_3^+$: 298.0972; Found: 298.0970.
IR (KBr): 3694, 3620, 3418, 2924, 2855, 2727, 2286, 2164, 2071, 2039, 1972, 1945, 1727, 1654, 1601, 1524, 1448, 1375, 1347, 1265, 1172, 1119, 2032, 916, 845, 745, 703 cm^{-1}.

^{1}H NMR (600 MHz, Chloroform-*d*)

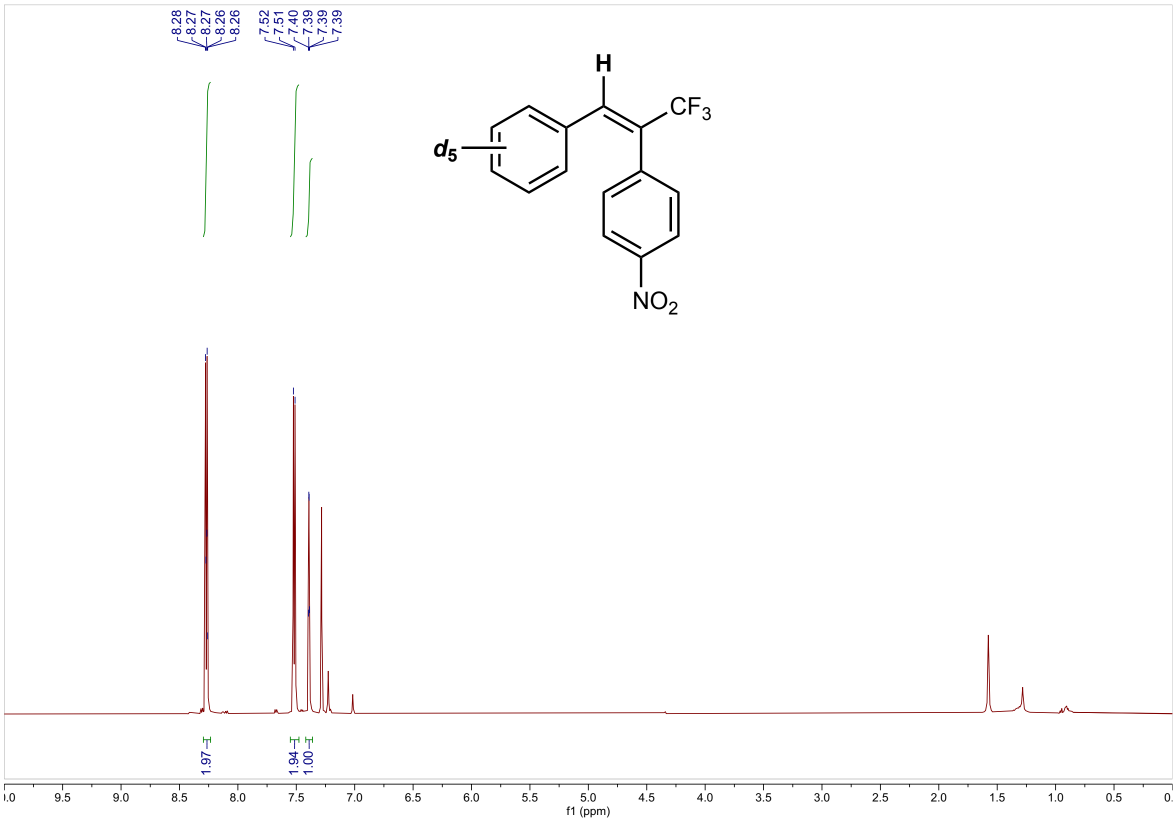

Investigation of Product Formation Rates

An oven-dried test tube was loaded with $Pd(TFA)_2$ (10 mol%), L6 (30 mol%), AgOAc (3 equiv.), benzene (5.0 equiv.) and the corresponding α-trifluoromethyl styrene (1 equiv., 0.05 mmol), then 0.25 mL of HFIP was added. The test tube was caped and stirred for the time indicated at 100 °C. The crude reaction mixture was analyzed by ^{19}F NMR using (trifluoromethyl)benzene as internal standard.

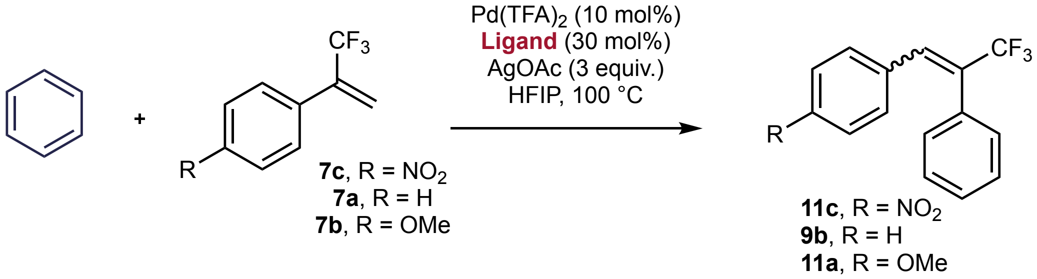

time / min	0	5	10	20	30	40	50	60	80	100	120
yield **9b**	0	4	14	21	29	41	52	64	80	86	86
yield **11c**	0	13	30	50	55	64	71	70	75	71	75
yield **11a**	0	10	25	47	65	98	-	-	-	-	-

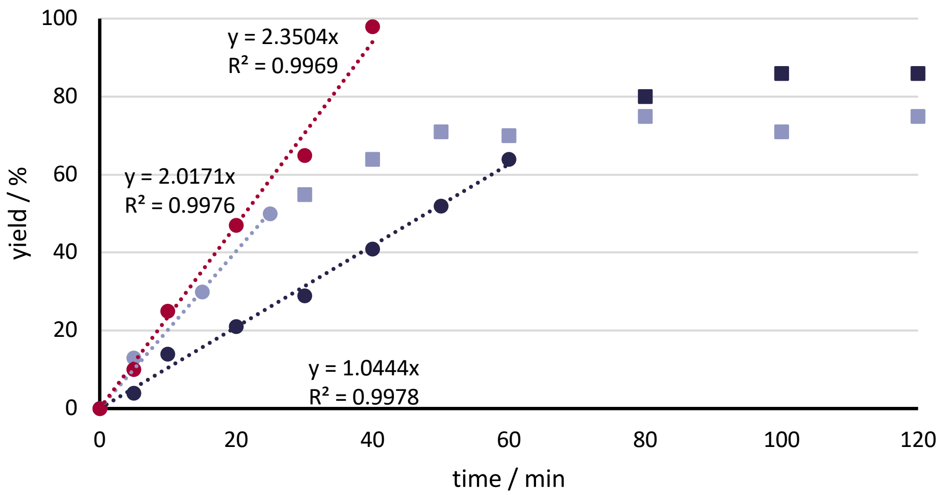

Figure S3: Reaction kinetics for the formation of **9b**, **11a** and **11c**.

Investigation of *E/Z* Ratio over time

An oven-dried test tube was loaded with $Pd(TFA)_2$ (10 mol%), L6 (30 mol%), AgOAc (3 equiv.), benzene (5.0 equiv.) and the corresponding α-trifluoromethyl styrene (1 equiv., 0.05 mmol), then 0.25 mL of HFIP was added. The test tube was caped and stirred for the time indicated at 100 °C. The crude reaction mixture was analyzed by ^{19}F NMR using (trifluoromethyl)benzene as internal standard.

For 1-Nitro-4-(3,3,3-trifluoroprop-1-en-2-yl)benzene

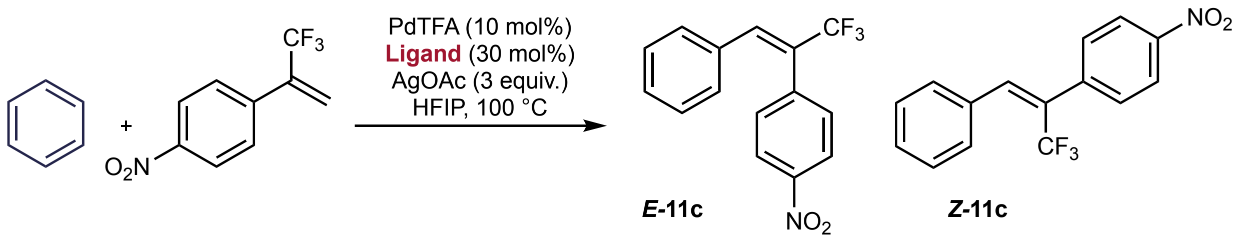

time / min	0	5	10	20	30	40	50	60	80	100	120
yield *E*-11c	0	7	10	20	29	42	52	64	79	84	84
yield *Z*-11c	0	1	1	2	4	6	7	8	10	10	10
**combined**	0	8	11	22	33	48	59	72	89	94	94

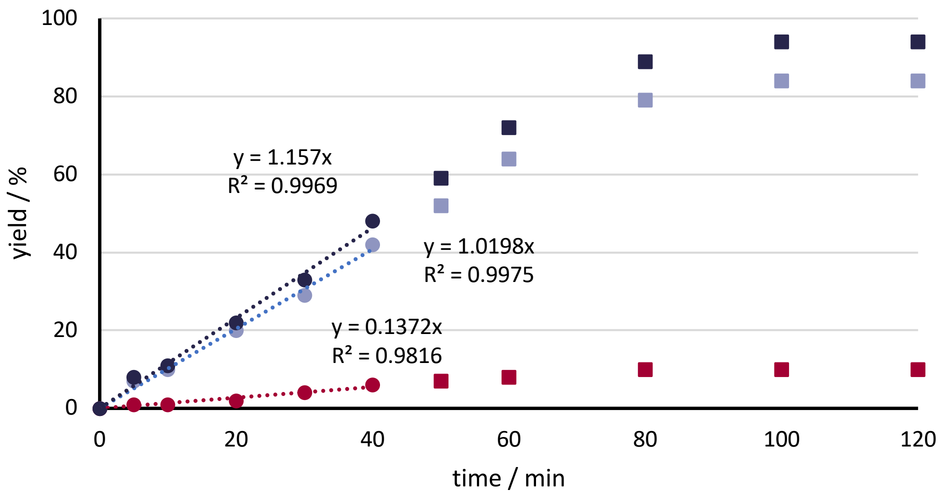

Figure S4: Reaction kinetics for the formation of ***E*-10c** and ***Z*-10c**.

For (3,3,3-Trifluoroprop-1-en-2-yl)benzene

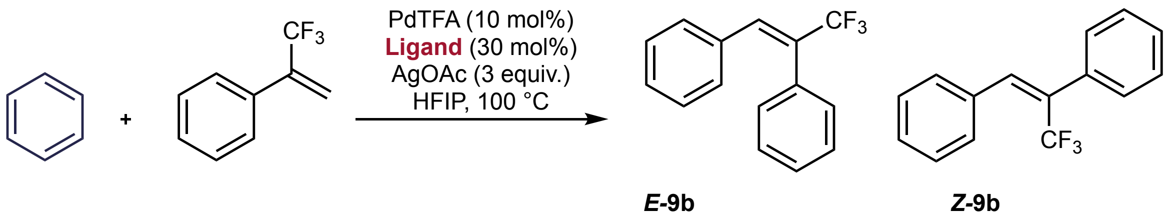

time / min	0	5	10	20	30	40	50	60	80	100	120
yield ***E*-9b**	0	8	20	32	36	41	47	48	53	40	54
yield ***Z*-9b**	0	5	10	18	19	23	24	22	22	21	21
**combined**	0	13	30	50	55	64	71	70	75	71	75

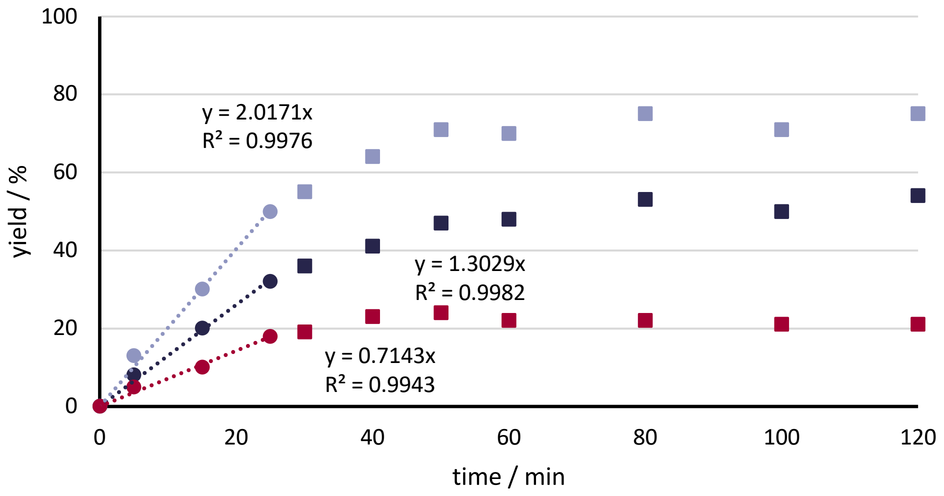

Figure S5: Reaction kinetics for the formation of ***E*-9b** and ***Z*-9b**.

For 1-Methoxy-4-(3,3,3-trifluoroprop-1-en-2-yl)benzene

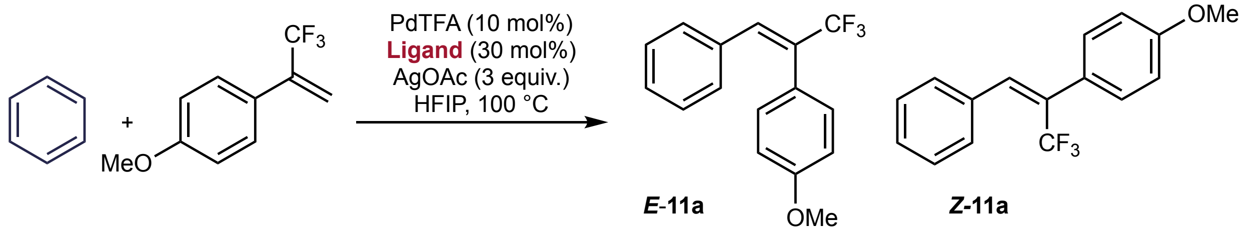

time / min	0	5	10	20	30	40
yield ***E*-11a**	0	7	17	35	48	74
yield ***Z*-11a**	0	3	8	12	17	24
combined	0	10	25	47	65	98

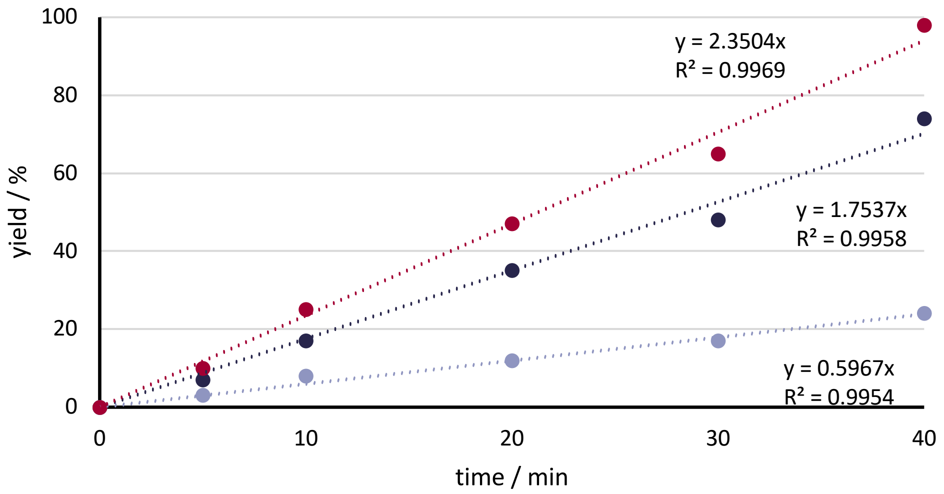

Figure S6: Reaction kinetics for the formation of ***E*-11a** and ***Z*-11a**.

6. Physical Data

(*E*)-2-(3,3,3-Trifluoro-2-phenylprop-1-en-1-yl)naphthalene (9a)

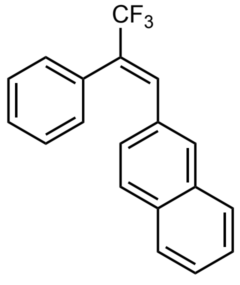

The title compound **9a** was synthesized according to the general procedure (GP-1), and was obtained after silica column chromatography (pure *n*-hexane) as a colorless oil (46%, 27.1 mg). crude *E/Z* = 4.1 : 1; curde β / α = 10.7 : 1.
^{1}H NMR (600 MHz, Chloroform-*d*): δ = 7.81 – 7.72 (m, 1H), 7.71 – 7.65 (m, 1H), 7.63 (s, 1H), 7.58 (d, *J* = 8.6 Hz, 1H), 7.50 – 7.41 (m, 6H), 7.39 – 7.33 (m, 2H), 7.00 (dd, *J* = 8.6, 1.8 Hz, 1H) ppm.
^{19}F NMR (565 MHz, Chloroform-*d*): δ= -65.67 (s) ppm.
^{13}C NMR{^{1}H, ^{19}F} (151 MHz, Chloroform-*d*): δ = 133.25, 133.21, 132.95 132.8, 131.1, 130.7, 130.0, 128.9, 128.8, 128.3, 127.7, 127.5, 126.9, 126.5, 126.3 ppm.
HRMS (ESI): *m/z*: $[M]^+$ Calcd. for $C_{19}H_{13}F_3^+$: 298.0963; Found: 298.0960.
IR (KBr): 3058, 2926, 2856, 2323, 2185, 2086, 1994, 1727, 1648, 1598, 1498, 1445, 1350, 1272, 1152, 1114, 954, 919, 855, 816, 746, 700 cm^{-1}.
The data is in line with the literature.[15]

(*E*)-(3,3,3-Trifluoroprop-1-ene-1,2-diyl)dibenzene (9b)

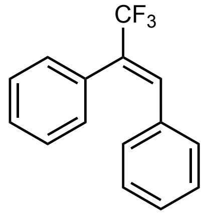

The title compound **9b** was synthesized according to the general procedure (GP-1) and was obtained after silica column chromatography (pure *n*-pentane) as a colorless oil (58%, 28.6 mg). crude *E/Z* = 2.6 : 1.
^{1}H NMR (600 MHz, Chloroform-*d*): δ = 7.45 – 7.39 (m, 2H), 7.39 – 7.30 (m, 2H), 7.27 – 7.22 (m, 2H), 7.21 – 7.15 (m, 2H), 7.08 – 7.01 (m, 2H) ppm.
^{19}F NMR (565 MHz, Chloroform-*d*): δ= -65.85 (s) ppm.
^{13}C NMR{^{1}H, ^{19}F} (151 MHz, Chloroform-*d*): δ = 133.5, 133.1, 132.7, 130.0, 129.8, 128.9, 128.8, 128.7, 128.2, 127.1, 123.8 ppm.
^{13}C NMR{^{1}H} (126 MHz, Chloroform-*d*): δ = 133.5, 133.1 (q, *J* = 5.6 Hz), 132.7, 130.2 (q, *J* = 29.8 Hz), 130.0, 129.8, 128.97, 128.90, 128.8, 128.2, 123.8 (q, *J* = 273.3 Hz) ppm.
HRMS (ESI): *m/z*: $[M]^+$ Calcd. for $C_{15}H_{11}F_3^+$: 248.0807; Found: 248.0806.
IR (KBr): 3066, 3030, 2926, 2856, 2322, 2104, 1957, 1891, 1813, 1730, 1650, 1600, 1575, 1492, 1446, 1389, 1323, 1270, 1218, 1142, 1106, 1029, 997, 955, 927, 904, 834, 759, 739, 695 cm^{-1}.
The data is in line with the literature.[16]

(*E*)-1-Methyl-4-(3,3,3-trifluoro-2-phenylprop-1-en-1-yl)benzene (9c)

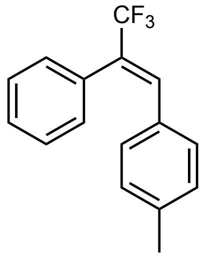

The title compound **9c** was synthesized according to the general procedure (GP-1) and was obtained after silica column chromatography (pure *n*-pentane) as a colorless oil (49%, 25.7 mg). cure *p*/*m* = 1.4/1, crude *E/Z* = 6.7 : 1.

^{1}H NMR (600 MHz, Chloroform-*d*): δ = 7.48 – 7.39 (m, 9.23H), 7.38 – 7.30 (m, 6.35H), 7.22 (s, 2.42H), 7.09 – 7.03 (m, 3.08H), 6.99 (d, *J* = 7.8 Hz, 2H), 6.92 (d, *J* = 7.8 Hz, 2H), 6.87 (s, 1.45H), 6.82 – 6.73 (m, 1.71H), 2.29 (s, 3H, minor product), 2.21 (s, 4.27H, major product) ppm.

^{19}F NMR (565 MHz, Chloroform-*d*): δ= -65.72 (s, 3F), -65.82 (s, 4.10F) ppm.

^{13}C NMR{^{1}H, ^{19}F} (151 MHz, Chloroform-*d*): δ = 139.1, 137.8, 133.4, 133.2, 133.0, 132.8, 132.1, 131.0, 130.7, 130.07, 130.03, 129.9, 129.8, 129.6, 129.0, 128.9, 128.8, 128.7, 128.6, 128.1, 127.0, 123.9, 123.8, 21.24, 21.23 ppm.

^{13}C NMR{^{1}H} (126 MHz, Chloroform-*d*): δ = 139.1, 137.8, 133.4, 133.2 (q, *J* = 6.2 Hz), 133.0 (q, *J* = 5.6 Hz), 132.9, 132.8, 131.0, 130.6, 130.0, 129.92, 129.90, 129.6, 129.0, 128.9, 128.9, 128.73, 128.71, 128.1, 127.0, 123.91 (q, *J* = 273.4 Hz), 123.85 (q, *J* = 273.3 Hz), 21.28, 21.26 ppm.

HRMS (ESI): *m/z*: [M]$^{+}$ Calcd. for $C_{16}H_{13}F_{3}^{+}$:262.0963; Found: 262.0962.

IR (KBr): 3728, 3059, 2925, 2859, 2342, 2228, 2203, 2170, 2120, 2025, 1992, 1945, 1879, 1807, 1747, 1687, 1650, 1605, 1489, 1446, 1382, 1274, 1245, 1149, 1114, 958, 912, 843, 812, 780, 739, 701 cm^{-1}.

(*E*)-1,2-Dimethyl-4-(3,3,3-trifluoro-2-phenylprop-1-en-1-yl)benzene (9d)

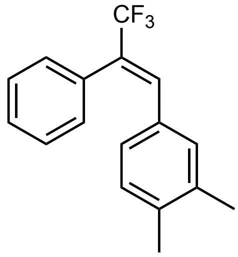

The title compound **9d** was synthesized according to the general procedure (GP-1) and was obtained after silica column chromatography (pure *n*-pentane) as a colorless oil (42%, 23.1 mg). crude *E/Z* = 7.6 : 1.

^{1}H NMR (600 MHz, Chloroform-*d*): δ = 7.53 – 7.38 (m, 3H), 7.33 (d, *J* = 4.5 Hz, 2H), 7.19 (s, 1H), 6.92 (d, *J* = 7.9 Hz, 1H), 6.82 (s, 1H), 6.72 (d, *J* = 7.9 Hz, 1H), 2.20 (s, 3H), 2.11 (s, 3H) ppm.

^{19}F NMR (565 MHz, Chloroform-*d*): δ= -65.71 (s) ppm.

^{13}C NMR{^{1}H, ^{19}F} (151 MHz, Chloroform-*d*): δ = 137.8, 136.4, 133.18, 133.14, 132.1, 131.6, 131.0, 129.9, 129.5, 129.0, 128.8, 128.6, 127.4, 123.9, 19.6, 19.5 ppm.

^{13}C NMR{^{1}H} (126 MHz, Chloroform-*d*): δ = 137.8, 136.4, 133.17 (q, *J* = 6.3 Hz), 133.12, 131.6, 131.0, 129.9, 129.5, 128.9, 128.6, 127.4, 123.9 (q, *J* = 273.3 Hz), 19.66, 19.60 ppm.

HRMS (ESI): *m/z*: [M]$^{+}$ Calcd. for $C_{17}H_{15}F_{3}^{+}$: 276.1120; Found: 276.1119.

IR (KBr): 3727, 3566, 3062, 2925, 2861, 2664, 2327, 2174, 2117, 2008, 1941, 1889, 1729, 1648, 1609, 1572, 1498, 1447, 1379, 1298, 1273, 1243, 1167, 1114, 1020, 916, 818, 773, 748, 701 cm^{-1}.

(*E*)-1,3-Dimethyl-5-(3,3,3-trifluoro-2-phenylprop-1-en-1-yl)benzene (9e)

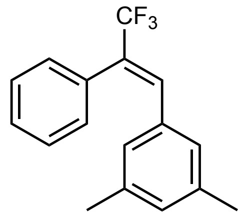

The title compound **9e** was synthesized according to the general procedure (GP-1), and was obtained after silica column chromatography (pure *n*-pentane) as a colorless oil (44%, 24.3 mg). crude *E/Z* = 6.4 : 1.
^{1}H NMR (600 MHz, Chloroform-*d*): δ = 7.44 – 7.38 (m, 4H), 7.38 – 7.27 (m, 3H), 7.19 (s, 1H), 6.87 (s, 1H), 6.63 (s, 2H), 2.15 (s, 6H) ppm.
^{19}F NMR (565 MHz, Chloroform-*d*): δ= -65.78 (s) ppm.
^{13}C NMR{^{1}H, ^{19}F} (151 MHz, Chloroform-*d*): δ = 137.6, 133.3, 133.3, 133.0, 130.5, 129.9, 129.8, 128.8, 128.6, 128.0, 123.8, 21.1 ppm.
HRMS (ESI): *m/z*: $[M]^+$ Calcd. for $C_{17}H_{15}F_3^+$: 276.1120; Found: 276.1118.
IR (KBr): 3022, 2922, 2864, 2323, 2117, 1880, 1732, 1648, 1600, 1492, 1445, 1382, 1293, 1255, 1150, 1114, 1031, 968, 928, 847, 819, 774, 748, 694 cm^{-1}.

(*E*)-1,4-Dimethyl-2-(3,3,3-trifluoro-2-phenylprop-1-en-1-yl)benzene (9f)

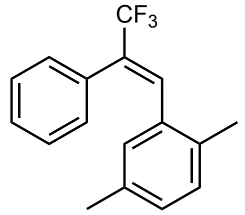

The title compound **9f** was synthesized according to the general procedure (GP-1), and was obtained after silica column chromatography (*n*-hexane : ethyl acetate 20:1 → 10:1) as a colorless oil (47%, 26.2 mg). crude *E/Z* = 3.8 : 1.
^{1}H NMR (600 MHz, Chloroform-*d*): δ = 7.41 (s, 1H), 7.35 – 7.27 (m, 2H), 7.23 (d, *J* = 6.7 Hz, 2H), 7.04 (d, *J* = 7.7 Hz, 1H), 6.93 (d, *J* = 7.8 Hz, 1H), 6.58 (s, 1H), 2.30 (s, 3H), 2.03 (s, 3H) ppm.
^{19}F NMR (565 MHz, Chloroform-*d*): δ= -64.96 (s) ppm.
^{13}C NMR{^{1}H, ^{19}F} (151 MHz, Chloroform-*d*): δ = 134.7, 133.9, 132.7, 132.6, 132.4, 131.2, 130.0, 129.9, 129.8, 129.1, 128.4, 128.3, 123.9, 20.6, 19.3 ppm.
HRMS (ESI): *m/z*: $[M]^+$ Calcd. for $C_{17}H_{15}F_3^+$: 276.1120; Found: 276.118.
IR (KBr): 3456, 2926, 2859, 2293, 2217, 2161, 2068, 2039, 1976, 1954, 1892, 1730, 1647, 1605, 1495, 1458, 1377, 1272, 1168, 1117, 1074, 1038, 999, 962, 910, 811, 775, 748, 700 cm^{-1}.

(*E*)-1-Methoxy-4-(3,3,3-trifluoro-1-phenylprop-1-en-2-yl)benzene (11a)

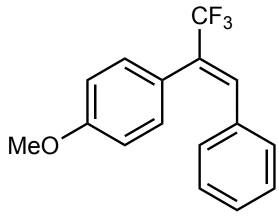

The title compound **11a** was synthesized according to the general procedure (GP-1), and was obtained after silica column chromatography (*n*-hexane : ethyl acetate 10:1 → 4:1) as a colorless oil (50%, 27.8 mg). crude *E/Z* = 2.6 : 1.

^{1}H NMR (600 MHz, Chloroform-*d*): δ = 7.26 – 7.17 (m, 6H), 7.10 – 7.03 (m, 2H), 6.98 – 6.88 (m, 2H), 3.86 (s, 3H) ppm.

^{19}F NMR (565 MHz, Chloroform-*d*): δ= -66.02 (s) ppm.

^{13}C NMR{^{1}H, ^{19}F} (151 MHz, Chloroform-*d*): δ = 159.9, 133.7, 132.9, 131.1, 130.0, 129.7, 128.7, 128.2, 124.7, 123.8, 114.4, 55.2 ppm.

HRMS (ESI): *m/z*: $[M]^+$ Calcd. for $C_{16}H_{13}OF_3^+$: 278.0913; Found: 278.0908.

IR (KBr): 3007, 2936, 2841, 2320, 2094, 1888, 1730, 1650, 1607, 1575, 1513, 1452, 1390, 1269, 1249, 1154, 1111, 1033, 958, 930, 897, 828, 755, 692 cm^{-1}.

(*E*)-1-chloro-4-(3,3,3-trifluoro-1-phenylprop-1-en-2-yl)benzene (11b)

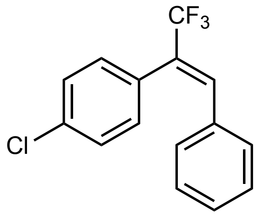

The title compound **11b** was synthesized according to the general procedure (GP-1) and was obtained after silica column chromatography (pure *n*-pentane) as a colorless oil (53%, 29.8 mg). crude *E/Z* = 8.4 : 1.

^{1}H NMR (600 MHz, Chloroform-*d*): δ = 7.42 – 7.36 (m, 2H), 7.36 – 7.24 (m, 4H), 7.25 – 7.18 (m, 2H), 7.05 (d, *J* = 7.6 Hz, 2H) ppm.

^{19}F NMR (565 MHz, Chloroform-*d*): δ= -65.78 (s) ppm.

^{13}C NMR{^{1}H, ^{19}F} (151 MHz, Chloroform-*d*): δ = 134.9, 133.8, 133.2, 131.3, 131.1, 129.9, 129.3, 129.1, 128.4, 128.2, 123.5 ppm.

HRMS (ESI): *m/z*: $[M]^+$ Calcd. for $C_{15}H_{10}ClF_3^+$: 282.0417; Found: 282.0416.

IR (KBr): 3036, 2928, 2321, 2094, 1996, 1901, 1729, 1651, 1593, 1492, 1449, 1392, 1323, 1271, 1221, 1154, 1115, 1017, 959, 934, 899, 823, 748, 693 cm^{-1}.

(*E*)-1-Nitro-4-(3,3,3-trifluoro-1-phenylprop-1-en-2-yl)benzene (11c)

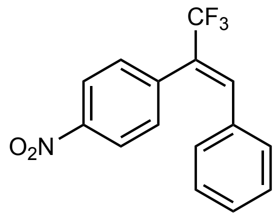

The title compound **11c** was synthesized according to the general procedure (GP-1), and was obtained after silica column chromatography (*n*-hexane : ethyl acetate 20:1 → 10:1) as a colorless oil (73%, 42.5 mg). crude *E/Z* = 8.6 : 1.
^{1}H NMR (600 MHz, Chloroform-*d*): δ = 8.32 – 8.19 (m, 2H), 7.53 – 7.46 (m, 2H), 7.39 (s, 1H), 7.32 – 7.25 (m, 2H), 7.23 (t, *J* = 7.6 Hz, 2H), 7.01 (d, *J* = 7.6 Hz, 2H) ppm.
^{19}F NMR (565 MHz, Chloroform-*d*): δ= -65.19 (s) ppm.
^{13}C NMR{^{1}H, ^{19}F} (151 MHz, Chloroform-*d*): δ = 148.1, 139.6, 135.1, 132.5, 131.2, 129.9, 129.5, 128.6, 124.1, 123.2 ppm.
^{13}C NMR{^{1}H} (126 MHz, Chloroform-*d*): δ =148.0, 139.6, 135.1 (q, *J* = 5.5 Hz), 132.5, 131.2, 129.9, 129.6, 128.6, 128.3 (q, *J* = 30.0 Hz), 123.2 (q, *J* = 273.4 Hz) 124.1 ppm.
HRMS (ESI): *m/z*: $[M]^+$ Calcd. for $C_{15}H_{10}O_2NF_3^+$: 293.0658; Found: 293.0658.
IR (KBr): 3081, 2933, 2859, 2452, 2100, 1919, 1798, 1725, 1651, 1600, 1522, 1493, 1450, 1396, 1343, 1273, 1218, 1148, 1110, 1017, 938, 910, 851, 754, 739, 698 cm^{-1}.

(*E*)-1-Chloro-3-(3,3,3-trifluoro-1-phenylprop-1-en-2-yl)benzene (11d)

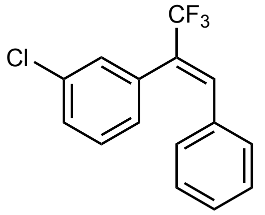

The title compound **11d** was synthesized according to the general procedure (GP-1) and was obtained after silica column chromatography (pure *n*-pentane) as a colorless oil (60%, 34.1 mg). crude *E/Z* = 6.2 : 1.
^{1}H NMR (600 MHz, Chloroform-*d*): = δ 7.41 (d, *J* = 8.4 Hz, 1H), 7.38 – 7.32 (m, 2H), 7.30 – 7.25 (m, 2H), 7.21 (q, *J* = 7.8 Hz, 3H), 7.04 (d, *J* = 7.6 Hz, 2H) ppm.
^{19}F NMR (565 MHz, Chloroform-*d*): δ= -65.71 (s) ppm.
^{13}C NMR{^{1}H, ^{19}F} (151 MHz, Chloroform-*d*): δ = 134.8, 134.4, 134.0, 133.0, 130.2, 130.0, 129.8, 129.2, 129.0, 128.4, 128.2, 123.5 ppm.
HRMS (ESI): *m/z*: $[M]^+$ Calcd. for $C_{15}H_{10}ClF_3^+$:282.0417; Found: 282.0415.
IR (KBr): 3066, 2929, 2324, 2096, 1950, 1731, 1651, 1594, 1565, 1477, 1450, 1414, 1383, 1323, 1272, 1221, 1156, 1114, 969, 929, 895, 834, 805, 785, 753, 717, 692 cm^{-1}.

(*E*)-3-(3,3,3-Trifluoro-1-phenylprop-1-en-2-yl)benzonitrile (11e)

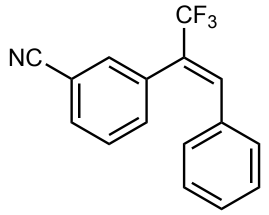

The title compound **11e** was synthesized according to the general procedure (GP-1) and was obtained after silica column chromatography (*n*-pentane : diethyl ether 80:1 → 40:1) as a colorless oil (71%, 38.7 mg). crude *E/Z* = 11.2 : 1.
^{1}H NMR (600 MHz, Chloroform-*d*): δ = 7.72 (dd, *J* = 7.4, 1.8 Hz, 1H), 7.62 (s, 1H), 7.59 – 7.48 (m, 2H), 7.36 (s, 1H), 7.31 – 7.26 (m, 1H), 7.26 – 7.19 (m, 2H), 6.99 (d, *J* = 7.6 Hz, 2H) ppm.
^{19}F NMR (565 MHz, Chloroform-*d*): δ= -65.55 (s) ppm.
^{13}C NMR{^{1}H, ^{19}F} (151 MHz, Chloroform-*d*): δ = 135.0, 134.6, 134.1, 133.5, 132.5, 132.4, 129.93, 129.90, 129.5, 128.6, 128.0, 123.3, 118.1, 113.4 ppm.
HRMS (ESI): *m/z*: [M]$^+$ Calcd. for $C_{16}H_{10}NF_3^+$:273.0759; Found: 273.0755.
IR (KBr): 3067, 2929, 2233, 2112, 1728, 1650, 1578, 1486, 1450, 1421, 1382, 1324, 1272, 1211, 1159, 1114, 1029, 981, 931, 903, 838, 801, 755, 692 cm^{-1}.

(*E*)-1-Methyl-2-(3,3,3-trifluoro-1-phenylprop-1-en-2-yl)benzene (11f)

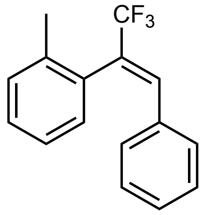

The title compound **11f** was synthesized according to the general procedure (GP-1), and was obtained after silica column chromatography (*n*-hexane : ethyl acetate 20:1 → 10:1) as a colorless oil (35%, 18.3 mg). crude *E/Z* = 5.2 : 1.
^{1}H NMR (600 MHz, Chloroform-*d*): δ = 7.42 – 7.35 (m, 1H), 7.35 – 7.22 (m, 5H), 7.18 (t, *J* = 7.6 Hz, 2H), 6.99 (d, *J* = 7.7 Hz, 2H), 2.15 (s, 3H) ppm.
^{19}F NMR (565 MHz, Chloroform-*d*): δ= -66.22 (s) ppm.
^{13}C NMR{^{1}H, ^{19}F} (151 MHz, Chloroform-*d*): δ = 137.3, 133.7, 133.5, 132.0, 130.6, 130.1, 129.5, 129.1, 129.0, 128.8, 128.7, 128.4, 127,2, 127.1, 126.3, 123.9, 19.4 ppm.
HRMS (ESI): *m/z*: [M]$^+$ Calcd. for $C_{16}H_{13}F_3^+$: 262.0963; Found: 262.0962.
IR (KBr): 3064, 3028, 2928, 2322, 2201, 2112, 2000, 1806, 1729, 1650, 1600, 1492, 1450, 1324, 1270, 1207, 1153, 1114, 1040, 1000, 955, 933, 901, 834, 759, 732, 691 cm^{-1}.

(*E*)-2-(3,3,3-Trifluoro-1-phenylprop-1-en-2-yl)naphthalene (11g)

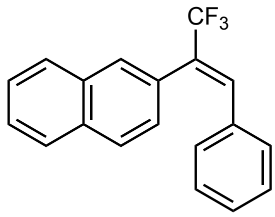

The title compound **11g** was synthesized according to the general procedure (GP-1) and was obtained after silica column chromatography (pure *n*-pentane) as a colorless oil (20%, 34.2 mg). crude *E/Z* = 3.4 : 1.

^{1}H NMR (600 MHz, Chloroform-*d*): δ = 7.94 – 7.76 (m, 3H), 7.68 – 7.49 (m, 2H), 7.42 – 7.31 (m, 2H), 7.23 – 7.17 (m, 1H), 7.17 – 7.11 (m, 2H), 7.10 – 7.03 (m, 3H) ppm.

^{19}F NMR (565 MHz, Chloroform-*d*): δ= -65.49 (s) ppm.

^{13}C NMR{^{1}H, ^{19}F} (151 MHz, Chloroform-*d*): δ = 133.4, 130.1, 129.1, 128.9, 128.6, 128.33, 128.32, 127.7, 127.4, 126.7, 126.3 ppm.

HRMS (ESI): *m/z*: $[M]^+$ Calcd. for $C_{19}H_{13}F_3^+$:298.0963; Found: 298.0961.

IR (KBr): 3059, 2928, 2322, 2100, 1730, 1650, 1599, 1498, 1449, 1390, 1321, 1271, 1220, 1155, 1112, 1021, 980, 934, 898, 856, 817, 749, 692 cm^{-1}.

(*E*)-5-(3,3,3-Trifluoro-1-phenylprop-1-en-2-yl)benzo[*d*][1,3]dioxole (11h)

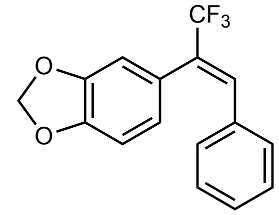

The title compound **11h** was synthesized according to the general procedure (GP-1), and was obtained after silica column chromatography (*n*-hexane : ethyl acetate 20:1 → 10:1) as a colorless oil (44%, 25.9 mg). crude *E/Z* = 7.3 : 1.

^{1}H NMR (600 MHz, Chloroform-*d*): δ = 7.17 – 7.09 (m, 3H), 7.00 (d, *J* = 7.4 Hz, 2H), 6.78 – 6.71 (m, 1H), 6.71 – 6.64 (m, 2H), 5.93 (s, 2H) ppm.

^{19}F NMR (565 MHz, Chloroform-*d*): δ= -66.06 (s) ppm.

^{13}C NMR{^{1}H, ^{19}F} (151 MHz, Chloroform-*d*): δ = 148.1, 148.0, 133.5, 133.3, 132.1, 130.0, 129.7, 128.9, 128.3, 125.9, 123.7, 110.1, 108.9, 101.3 ppm

HRMS (ESI): *m/z*: $[M]^+$ Calcd. for $C_{16}H_{11}O_2F_3^+$: 292.0705; Found: 292.0704.

IR (KBr): 3066, 2900, 2780, 2059, 1859, 1727, 1651, 1607, 1492, 1446, 1388, 1330, 1270, 1236, 1155, 1110, 1038, 971, 933, 890, 865, 812, 758, 735, 694 cm^{-1}.

(*E*)-1-Methyl-4-(3,3,3-trifluoro-2-(4-nitrophenyl)prop-1-en-1-yl)benzene (12a)

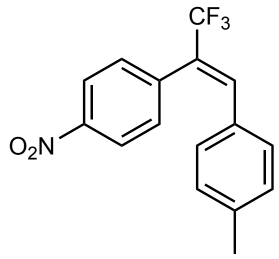

The title compound **12a** was synthesized according to the general procedure (GP-1), and was obtained after silica column chromatography (*n*-hexane : ethyl acetate 20:1 → 10:1) as a colorless oil (72%, 44.4 mg). crude *E/Z* = 9.5 : 1, *p/m* = 2.3 : 1.

^{1}H NMR (600 MHz, Chloroform-*d*): δ = 8.21 – 8.09 (m, 6.34H), 7.47 – 7.31 (m, 5.40H), 7.33 – 7.20 (m, 3.59H), 7.04 – 6.95 (m, 4.40H), 6.92 (d, *J* = 8.0 Hz, 2.10H), 6.79 (d, *J* = 7.6 Hz, 3.79H), 6.66 – 6.50 (m, 2.81H), 2.20 (s, 3.00H), 2.13 (s, 6.47H) ppm.

^{19}F NMR (565 MHz, Chloroform-*d*): δ= -65.02 (s, 3F, minor product), -65.12 (s, 7.49F, major product) ppm.

^{13}C NMR{^{1}H, ^{19}F} (151 MHz, Chloroform-*d*): δ = 148.0, 139.99, 139.94, 139.7, 138.3, 135.3, 135.0, 132.5, 131.27, 131.25, 130.9, 130.3, 129.9, 129.3, 128.4, 126.7, 124.1, 124.0, 21.28, 21.24 ppm.

HRMS (ESI): *m/z*: $[M]^+$ Calcd. for $C_{16}H_{12}ON_2F_3^+$: 307.0814; Found: 307.0811.

IR (KBr): 3031, 2926, 2861, 2298, 2103, 1925, 1803, 1731, 1649, 1600, 1521, 1381, 1346, 1276, 1246, 1172, 1150, 1112, 1017, 961, 924, 852, 815, 784, 744, 706 cm^{-1}.

(*E*)-1,2-Dimethyl-4-(3,3,3-trifluoro-2-(4-nitrophenyl)prop-1-en-1-yl)benzene (12b)

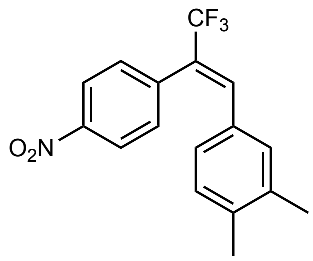

The title compound **12b** was synthesized according to the general procedure (GP-1), and was obtained after silica column chromatography (*n*-hexane : ethyl acetate 20:1 → 10:1) as a colorless oil (73, 47.2 mg). crude *E/Z* = 10.0 : 1.

^{1}H NMR (600 MHz, Chloroform-*d*): δ = 7.50 – 7.38 (m, 2H), 7.38 – 7.30 (m, 2H), 7.19 (s, 1H), 6.92 (d, *J* = 7.9 Hz, 1H), 6.82 (s, 1H), 6.72 (d, *J* = 7.9 Hz, 1H), 2.20 (s, 3H), 2.11 (s, 3H) ppm.

^{19}F NMR (565 MHz, Chloroform-*d*): δ= -65.71 (s) ppm.

^{13}C NMR{^{1}H, ^{19}F} (151 MHz, Chloroform-*d*): δ = 137.8, 136.4, 133.17, 133.14, 131.6, 131.07, 129.9, 129.5, 129.0, 128.8, 128.6, 127.4, 123.9, 19.6, 19.5 ppm.

HRMS (ESI): *m/z*: $[M]^+$ Calcd. for $C_{16}H_{12}O_2NF_3^+$: 307.0814; Found: 307.0806.

IR (KBr): 3031, 2926, 2861, 2325, 2092, 1927, 1802, 1737, 1649, 1600, 1521, 1382, 1346, 1276, 1246, 1173, 1149, 1113, 1017, 961, 924, 852, 814, 784, 744, 706 cm^{-1}.

(*E*)-1,3-Dimethyl-5-(3,3,3-trifluoro-2-(4-nitrophenyl)prop-1-en-1-yl)benzene (12c)

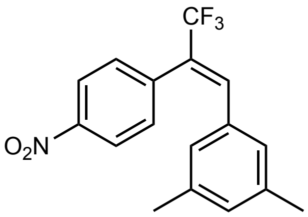

The title compound **12c** was synthesized according to the general procedure (GP-1), and was obtained after silica column chromatography (*n*-hexane : ethyl acetate 20:1 → 10:1) as a colorless oil (85%, 54.9 mg). crude *E/Z* = 11.3 : 1.

^{1}H NMR (600 MHz, Chloroform-*d*): δ = 8.36 – 8.18 (m, 2H), 7.51 (d, *J* = 8.3 Hz, 2H), 7.33 (s, 1H), 6.92 (s, 1H), 6.62 (s, 2H), 2.16 (s, 6H) ppm.

^{19}F NMR (565 MHz, Chloroform-*d*): δ= -65.05 (s) ppm.

^{13}C NMR{^{1}H, ^{19}F} (151 MHz, Chloroform-*d*): δ = 147.9, 139.9, 138.1, 135.4, 132.4, 131.26, 131.25, 131.0, 127.8, 123.9, 123.3, 21.1 ppm.

HRMS (ESI): *m/z*: [M]$^+$ Calcd. for $C_{17}H_{14}O_2NF_3^+$:321.0971; Found: 321.0967.

IR (KBr): 2922, 2863, 2329, 2094, 1924, 1741, 1648, 1600, 1522, 1457, 1382, 1346, 1292, 1255, 1173, 1150, 1113, 1018, 971, 930, 851, 727, 694 cm^{-1}.

(*E*)-1,4-Dimethyl-2-(3,3,3-trifluoro-2-(4-nitrophenyl)prop-1-en-1-yl)benzene (12d)

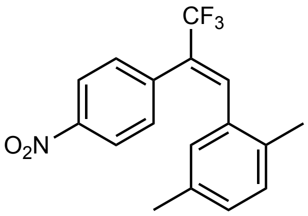

The title compound **12d** was synthesized according to the general procedure (GP-1), and was obtained after silica column chromatography (*n*-hexane : ethyl acetate 20:1 → 10:1) as a colorless oil (74%, 37.2 mg). crude *E/Z* = 13.5 : 1. *p/m* = 1.4 : 1

^{1}H NMR (600 MHz, Chloroform-*d*): δ = 8.47 – 8.24 (m, 0.43H), 8.21 – 8.11 (m, 2.33H), 7.68 (d, *J* = 8.3 Hz, 0.41H), 7.55 (s, 1H), 7.41 (d, *J* = 8.3 Hz, 1.09H), 7.23 (s, 2.10H), 7.17 – 7.08 (m, 1.01H), 7.07 (d, *J* = 7.8 Hz, 1.00H), 6.98 (d, *J* = 7.8 Hz, 1.08H), 6.52 (s, 1.02H), 2.37 (s, 0.58H), 2.32 (s, 0.56H), 2.31 (s, 3.16H), 2.05 (s, 3H) ppm.

^{19}F NMR (565 MHz, Chloroform-*d*): δ= -56.82 (s, 1F), -64.40 (s, 6.11F) ppm.

^{13}C NMR{^{1}H, ^{19}F} (151 MHz, Chloroform-*d*): δ = 140.9, 139.5, 135.2, 135.1, 133.9, 131.8, 131.0, 130.3, 129.9, 129.8, 129.68, 129.62, 129.4, 129.1, 128.6, 123.7, 123.5, 123.3, 20.9, 20.7, 19.5, 19.3 ppm.

HRMS (ESI): *m/z*: [M]$^+$ Calcd. for $C_{17}H_{14}O_2F_3N^+$: 321.0971; Found: 321.0970.

IR (KBr): 3074, 2928, 2862, 2289, 2167, 2107, 1986, 1804, 1729, 1651, 1597, 1518, 1384, 1348, 1292, 1272, 1213, 1173, 1152, 1103, 1004, 964, 920, 850, 815, 773, 706 cm^{-1}.

(*E*)-1-Isobutyl-4-(3,3,3-trifluoro-2-(4-nitrophenyl)prop-1-en-1-yl)benzene (12e)

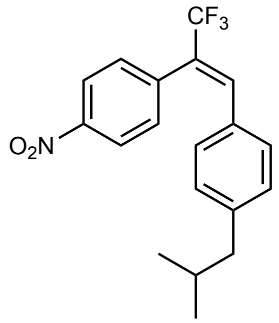

The title compound **12e** was synthesized according to the general procedure (GP-1), and was obtained after silica column chromatography (*n*-hexane : ethyl acetate 20:1 → 10:1) as a colorless oil (95%, 66.3 mg). crude *E/Z* = 11.9 : 1. *p/m* = 2.8 : 1.

^{1}H NMR (600 MHz, Chloroform-*d*): δ = 8.31 – 8.18 (m, 7.06H), 7.50 (t, *J* = 9.6 Hz, 6.90H), 7.36 (s, 2.31H), 7.32 (s, 1.11H), 7.18 – 7.07 (m, 2.58H), 7.04 (d, *J* = 7.6 Hz, 2.27H), 7.01 – 6.94 (m, 2.31H), 6.94 – 6.87 (m, 2.32H), 6.82 (d, *J* = 7.7 Hz, 2.29H), 6.75 (s, 2.36H), 2.41 (d, *J* = 7.1 Hz, 2.23H), 2.29 (d, *J* = 7.2 Hz, 4.54H), 1.80 (dt, *J* = 13.4, 6.7 Hz, 1H), 1.62 (dt, *J* = 13.5, 6.8 Hz, 1.87H), 0.85 (dd, *J* = 6.7, 1.7 Hz, 6H), 0.75 (dd, *J* = 6.8, 1.8 Hz, 14.26H) ppm.

^{19}F NMR (565 MHz, Chloroform-*d*): δ= -65.04 (s, 3F, minor product), -65.11 (s, 7.56F, major product) ppm.

^{13}C NMR{^{1}H, ^{19}F} (151 MHz, Chloroform-*d*): δ = 147.9, 143.8, 142.0, 139.9, 139.8, 135.5, 135.0, 132.3, 131.29, 131.27, 130.8, 130.4, 129.8, 129.3, 128.3, 127.2, 124.1, 124.0, 123.3, 45.0, 30.0, 22.2, 22.1 ppm.

HRMS (ESI): *m/z*: $[M]^+$ Calcd. for $C_{19}H_{18}O_2NF_3^+$: 349.1284; Found: 349.1280.

IR (KBr): 2958, 2870, 2291, 2086, 1944, 1801, 1733, 1649, 1600, 1522, 1464, 1345, 1275, 1172, 1150, 1114, 1018, 957, 926, 852, 789, 744, 707 cm^{-1}.

(*E*)-1-(*tert*-Butyl)-4-(3,3,3-trifluoro-2-(4-nitrophenyl)prop-1-en-1-yl)benzene (12f)

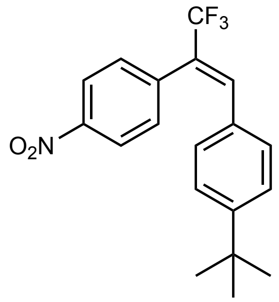

The title compound **12f** was synthesized according to the general procedure (GP-1), and was obtained after silica column chromatography (*n*-hexane : ethyl acetate 20:1 → 10:1) as a colorless oil (82%, 57.7 mg). crude *E/Z* = 21.7 : 1, crude *p/m* = 1.9 : 1.

^{1}H NMR (600 MHz, Chloroform-*d*): δ = 8.35 – 8.20 (m, 5.78H), 7.54 (dt, *J* = 8.8, 2.1 Hz, 5.86H), 7.40 (s, 2.06H), 7.35 – 7.29 (m, 3.18H), 7.26 – 7.21 (m, 2.17H), 7.18 (td, *J* = 7.8, 1.8 Hz, 1.85H), 7.00 (d, *J* = 2.1 Hz, 2.05H), 6.94 (dd, *J* = 8.5, 1.9 Hz, 2.10H), 6.87 (d, *J* = 7.7 Hz, 1.90H), 1.28 (d, *J* = 1.9 Hz, 9H), 1.13 (d, *J* = 1.8 Hz, 16.21H) ppm.

^{19}F NMR (565 MHz, Chloroform-*d*): δ= -65.07 (s, 3F, minor product), -65.09 (s, 5.43F, major product) ppm.

^{13}C NMR{^{1}H, ^{19}F} (151 MHz, Chloroform-*d*): δ = 153.2, 151.4, 148.1, 148.02, 140.08, 140.01, 135.6, 134.8, 132.5, 132.19, 132.15, 131.3, 131.2, 129.9, 129.6, 128.4, 127.8, 127.29, 127.27, 127.1, 126.6, 125.6, 124.1, 124.0, 123.4, 123.3, 31.0, 30.9 ppm.

HRMS (ESI): *m/z*: $[M]^+$ Calcd. for $C_{19}H_{18}O_2F_3N^+$: 349.1284; Found: 349.1282.

IR (KBr): 3077, 2963, 2869, 2294, 2119, 1924, 1802, 1733, 1648, 1600, 1522, 1483, 1346, 1272, 1221, 1157, 1113, 1018, 966, 924, 852, 796, 752, 699, 662 cm^{-1}.

(*E*)-1-cyclohexyl-4-(3,3,3-trifluoro-2-(4-nitrophenyl)prop-1-en-1-yl)benzene (12g)

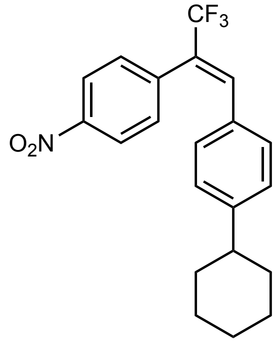

The title compound **12g** was synthesized according to the general procedure (GP-1), and was obtained after silica column chromatography (*n*-hexane : ethyl acetate 20:1 → 10:1) as a colorless oil (76%, 57.2 mg). crude *E/Z* = 10.8 : 1, crude *p/m* = 2.3 : 1.
^{1}H NMR (600 MHz, Chloroform-*d*): δ = 8.32 – 8.23 (m, 2.67H), 7.58 – 7.48 (m, 2.58H), 7.37 (s, 1H), 7.33 (s, 0.44H), 7.19 – 7.09 (m, 1.86H), 7.09 – 7.03 (m, 0.86H), 6.96 – 6.90 (m, 0.83H), 6.84 (s, 0.88H), 6.84 – 6.76 (m, 0.82H), 2.52 – 2.41 (m, 0.73H), 2.35 (td, *J* = 11.9, 5.8 Hz, 1H), 1.98 – 1.65 (m, 7.59H), 1.45 – 1.26 (m, 3.64H), 1.26 – 1.04 (m, 3.50H) ppm.
^{19}F NMR (565 MHz, Chloroform-*d*): δ= -65.03 (s, 3F, minor product), -65.11 (s, 7.37F, major product) ppm.
^{13}C NMR{^{1}H, ^{19}F} (151 MHz, Chloroform-*d*): δ = 150.0, 148.3, 148.0, 140.0, 135.5, 134.9, 132.4, 131.3, 131.2, 130.1, 129.9, 128.6, 128.5, 128.4, 127.9, 127.3, 127.1, 124.1, 124.0, 123.3, 44.3, 44.0, 34.1, 34.1, 26.7, 26.6, 26.0 ppm.
HRMS (ESI): *m/z*: $[M]^+$ Calcd. for $C_{21}H_{20}O_2NF_3^+$:375.1440; Found: 375.1431.
IR (KBr): 3057, 2926, 2854, 2301, 2119, 1939, 1732, 1648, 1600, 1522, 1448, 1345, 1316, 1277, 1172, 1150, 1113, 1016, 967, 929, 852, 795, 766, 747, 707 cm^{-1}.

(*E*)-5-(3,3,3-Trifluoro-2-(4-nitrophenyl)prop-1-en-1-yl)-2,3-dihydro-1*H*-indene (12h)

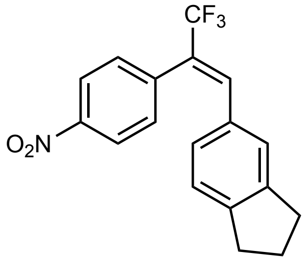

The title compound **12h** was synthesized according to the general procedure (GP-1), and was obtained after silica column chromatography (*n*-hexane : ethyl acetate 20:1 → 10:1) as a colorless oil (72%, 48.1 mg). crude *E/Z* = 8.6 : 1, crude 5/4 position = 2.5 : 1, ratio: 5/4 position = 1 : 3.7.

^{1}H NMR (600 MHz, Chloroform-*d*): δ = 8.30 – 8.24 (m, 7.43H), 8.24 – 8.17 (m, 2.10H), 7.56 – 7.51 (m, 8.07H), 7.51 – 7.43 (m, 3.73H), 7.36 (s, 3.71H), 7.15 (d, *J* = 7.3 Hz, 1.41H), 7.05 (d, *J* = 7.8 Hz, 3.44H), 6.89 (s, 3.33H), 6.86 (t, *J* = 7.6 Hz, 1.09H), 6.75 (d, *J* = 7.8 Hz, 3.49H), 6.50 (d, *J* = 7.8 Hz, 1H), 2.93 (dt, *J* = 15.3, 7.6 Hz, 6.48H), 2.87 (t, *J* = 7.5 Hz, 7.12H), 2.78 (t, *J* = 7.5 Hz, 7.11H), 2.23 – 2.09 (m, 2.99H), 2.11 – 2.00 (m, 9.43H) ppm.

^{19}F NMR (565 MHz, Chloroform-*d*): δ= -64.64 (s, 3F, minor product), -64.89 (s, 11.03F, major product) ppm.

^{13}C NMR{^{1}H, ^{19}F} (151 MHz, Chloroform-*d*): δ = 146.4, 144.8, 140.1, 135.60, 133.65, 131.2, 131.1, 130.4, 128.1, 126.29, 126.24, 126.0, 125.4, 124.4, 124.0, 123.8, 123.4, 32.8, 32.7, 32.60, 31.65, 25.2, 24.8 ppm.

HRMS (ESI): *m/z*: $[M]^+$ Calcd. for $C_{18}H_{14}O_2NF_3^+$: 333.0970; Found: 333.0971.

IR (KBr): 3077, 2951, 2849, 2318, 2106, 1926, 1726, 1546, 1599, 1521, 1437, 1378, 1345, 1280, 1234, 1166, 1111, 1017, 966, 923, 852, 822, 783, 746, 706 cm^{-1}.

(*E*)-1-Methoxy-4-(3,3,3-trifluoro-2-(4-nitrophenyl)prop-1-en-1-yl)benzene (12i)

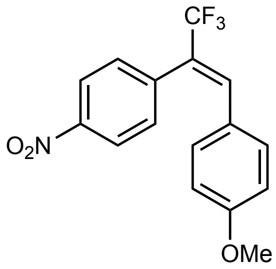

The title compound **12i** was synthesized according to the general procedure (GP-1), and was obtained after silica column chromatography (*n*-hexane : ethyl acetate 10:1 → 40:1) as a colorless oil (73%, 47.2 mg). crude *E/Z* = 11.2 : 1. *p/m/o* = 4.6 : 2.1 : 1.

^{1}H NMR (600 MHz, Chloroform-*d*): δ = 8.35 – 8.23 (m, 3H), 8.23 – 8.18 (m, 0.43H), 7.67 (s, 0.25H), 7.52 (d, *J* = 8.2 Hz, 3H), 7.49 (d, *J* = 8.3 Hz, 0.45H), 7.35 (s, 1H), 7.35 – 7.27 (m, 0.75H), 7.25 (dd, *J* = 8.6, 4.5 Hz, 0.17H), 7.14 (t, *J* = 8.0 Hz, 1H), 6.94 (d, *J* = 8.5 Hz, 0.78H), 6.89 (d, *J* = 8.3 Hz, 0.23H), 6.83 (dd, *J* = 8.3, 2.5 Hz, 1H), 6.75 – 6.71 (m, 0.84H), 6.65 (d, *J* = 4.6 Hz, 0.43H), 6.60 (d, *J* = 7.7 Hz, 1H), 6.53 (d, *J* = 2.7 Hz, 1H), 3.87 (s, 0.84H), 3.83 – 3.74 (m, 1.30H), 3.62 (s, 3H) ppm.

^{19}F NMR (565 MHz, Chloroform-*d*): δ= -64.50 (s, 3F, minor product), -64.82 (s, 5.18F, minor product), -65.22 (s, 12.50F, major product) ppm.

^{13}C NMR{^{1}H, ^{19}F} (151 MHz, Chloroform-*d*): δ = 160.6, 159.4, 157.9, 148.0, 140.1, 139.6, 135.0, 134.5, 133.8, 131.7, 131.3, 131.28, 131.25, 131.0, 130.9, 130.2, 129.6, 128.5, 125.8, 124.9, 124.1, 124.0, 123.8, 123.5, 123.2, 122.4, 120.2, 115.3, 115.1, 114.0, 110.7, 55.4, 55.2, 55.0 ppm.

HRMS (ESI): *m/z*: $[M]^+$ Calcd. for $C_{16}H_{12}O_3NF_3^+$: 323.0763; Found: 323.0758.

IR (KBr): 3079, 3007, 2939, 2843, 2326, 2092, 1925, 1733, 1648, 1599, 1520, 1491, 1463, 1384, 1346, 1257, 1152, 1112, 1037, 963, 920, 851, 760, 707 cm^{-1}.

(*E*)-1-(3-(3,3,3-trifluoro-2-(4-nitrophenyl)prop-1-en-1-yl)phenyl)ethan-1-one (12j)

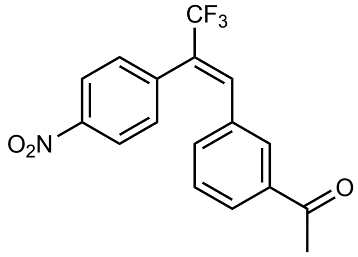

The title compound **12j** was synthesized according to the general procedure (GP-1), and was obtained after silica column chromatography (*n*-hexane : ethyl acetate 10:1 → 40:1) as a colorless oil (64%, 42.9 mg). crude *E/Z* = 6.2 : 1. *m/p* = 2.4 : 1.

^{1}H NMR (600 MHz, Chloroform-*d*): δ = 8.34 – 8.24 (m, 7.68H), 7.86 (d, *J* = 7.8 Hz, 2.31H), 7.81 (d, *J* = 8.4 Hz, 2.08H), 7.67 (s, 3.33H), 7.57 – 7.48 (m, 6.40H), 7.48 – 7.41 (m, 4.24H), 7.32 (t, *J* = 7.8 Hz, 2.47H), 7.15 (d, *J* = 7.8 Hz, 2.41H), 7.11 (d, *J* = 8.2 Hz, 2.07H), 2.57 (s, 3.00H), 2.46 (s, 7.06H) ppm.

^{19}F NMR (565 MHz, Chloroform-*d*): δ= -65.47 (s, 7.45F, major product), -65.50 (s, 3.00F, minor product) ppm.

^{13}C NMR{^{1}H, ^{19}F} (151 MHz, Chloroform-*d*): δ = 134.1, 134.0, 133.8, 133.1, 131.1, 131.09, 130.03, 129.9, 129.1, 128.9, 128.4, 124.2, 123.8, 26.5, 26.3 ppm.

HRMS (ESI): *m/z*: $[M]^+$ Calcd. for $C_{17}H_{12}O_3NF_3^+$: 335.0763; Found: 335.0757.

IR (KBr): 3078, 2927, 2857, 2343, 2185, 2077, 1937, 1727, 1686, 1599, 1522, 1464, 1429, 1348, 1268, 1212, 1157, 1115, 957, 920, 852, 801, 755, 699 cm^{-1}.

Methyl (*E*)-3-(3,3,3-trifluoro-2-(4-nitrophenyl)prop-1-en-1-yl)benzoate (12k)

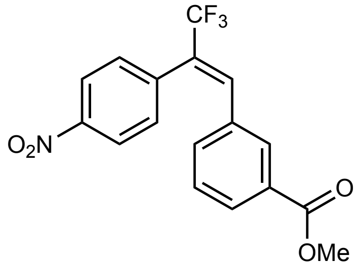

The title compound **12k** was synthesized according to the general procedure (GP-1), and was obtained after silica column chromatography (*n*-hexane : ethyl acetate 10:1 → 40:1) as a colorless oil (77%, 54.2 mg). crude *E/Z* = 12.5 : 1. *m/p* = 4.6 : 1.8 : 1.

^{1}H NMR (600 MHz, Chloroform-*d*): δ = 8.36 – 8.31 (m, 1.02H), 8.27 (t, *J* = 8.7 Hz, 6.55H), 8.11 (d, *J* = 8.5 Hz, 1.30H), 7.94 (d, *J* = 7.8 Hz, 2.79H), 7.89 (d, *J* = 8.1 Hz, 2.10H), 7.75 (s, 0.71H), 7.68 (d, *J* = 8.3 Hz, 7.87H), 7.50 (t, *J* = 8.7 Hz, 3.81H), 7.42 (s, 0.69H), 7.39 (d, *J* = 8.4 Hz, 2H), 7.28 (d, *J* = 7.3 Hz, 2.54H), 7.13 (d, *J* = 7.8 Hz, 2.16H), 7.09 (d, *J* = 8.1 Hz, 3.00H), 3.90 (s, 1H), 3.86 (s, 7.12H) ppm.

^{19}F NMR (565 MHz, Chloroform-*d*): δ= -64.58 (s, 3F, minor product), -65.48 (s, 8.63, major product) ppm.

^{13}C NMR{^{1}H, ^{19}F} (151 MHz, Chloroform-*d*): δ = 134.18, 134.13, 133.6, 131.2, 131.1, 131.0, 130.4, 129.77, 129.75, 128.7, 124.2, 124.1 ppm.

HRMS (ESI): *m/z*: $[M]^+$ Calcd. for $C_{17}H_{12}O_4NF_3^+$: 351.0712; Found: 351.0700.

IR (KBr): 3079, 2954, 2857, 2332, 2168, 2075, 1930, 1723, 1651, 1600, 1522, 1438, 1347, 1277, 1216, 1158, 1112, 1017, 959, 926, 853, 810, 749, 713 cm^{-1}.

7. Spectra

(*E*)-2-(3,3,3-Trifluoro-2-phenylprop-1-en-1-yl)naphthalene (9a)

^{1}H NMR (600 MHz, Chloroform-*d*)

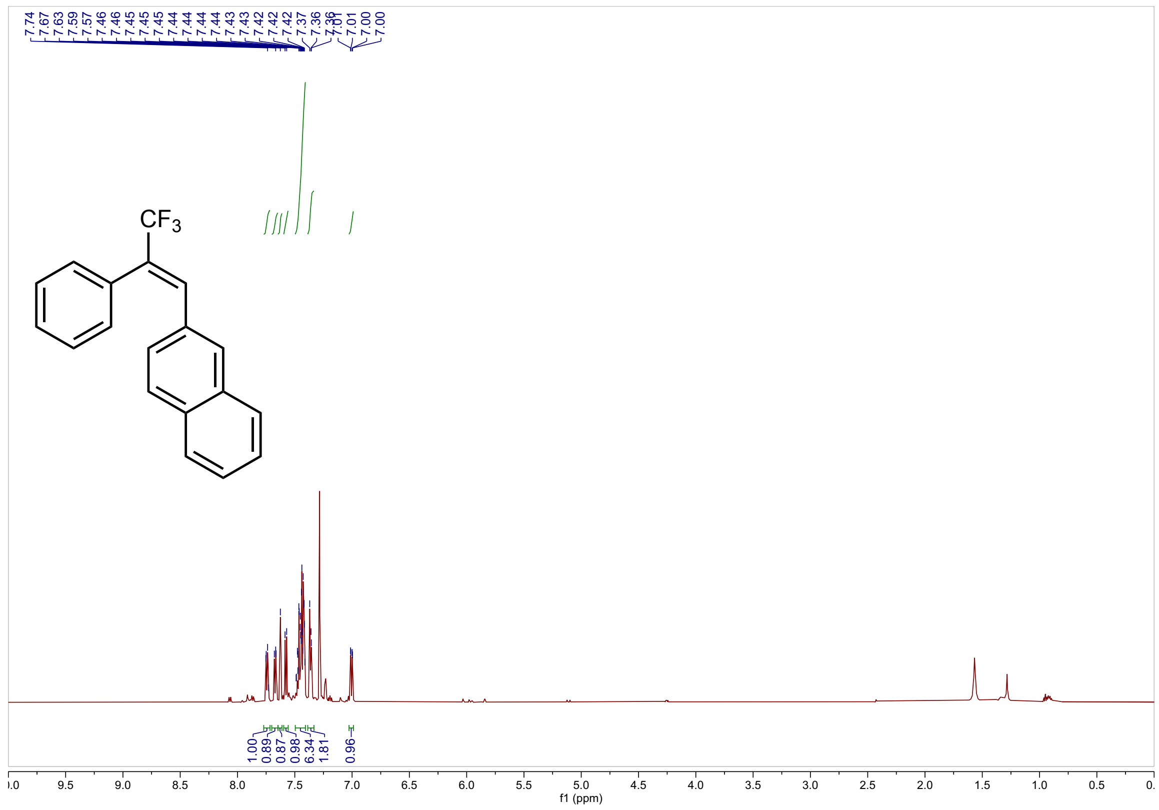

^{19}F NMR (565 MHz, Chloroform-*d*)

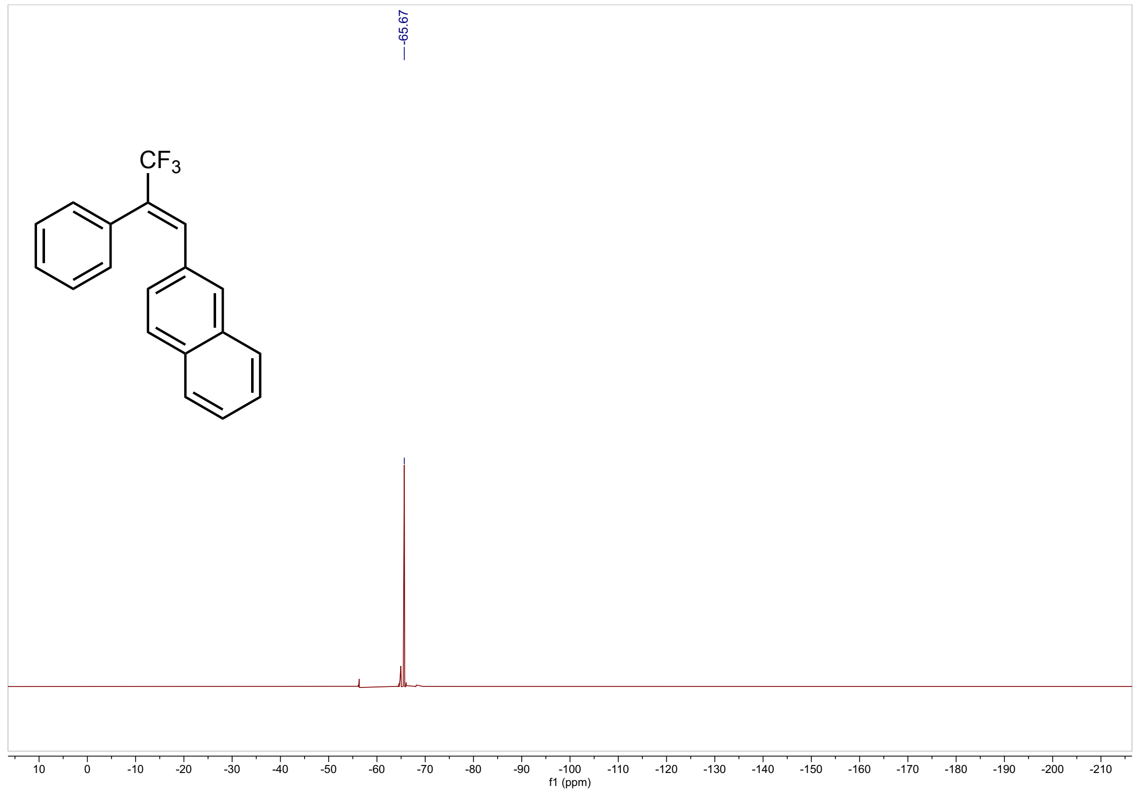

^{13}C NMR{^{1}H, ^{19}F} (151 MHz, Chloroform-*d*)

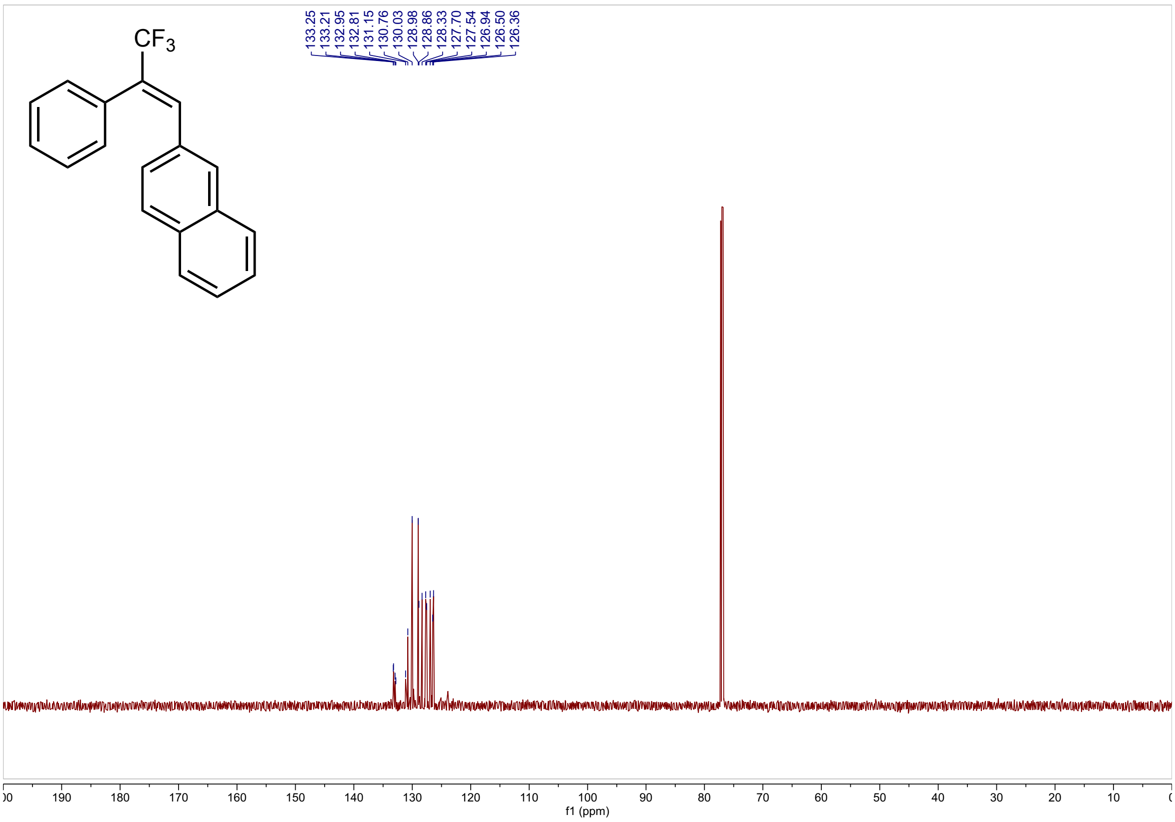

(*E*)-(3,3,3-Trifluoroprop-1-ene-1,2-diyl)dibenzene (9b)

^{1}H NMR (600 MHz, Chloroform-*d*)

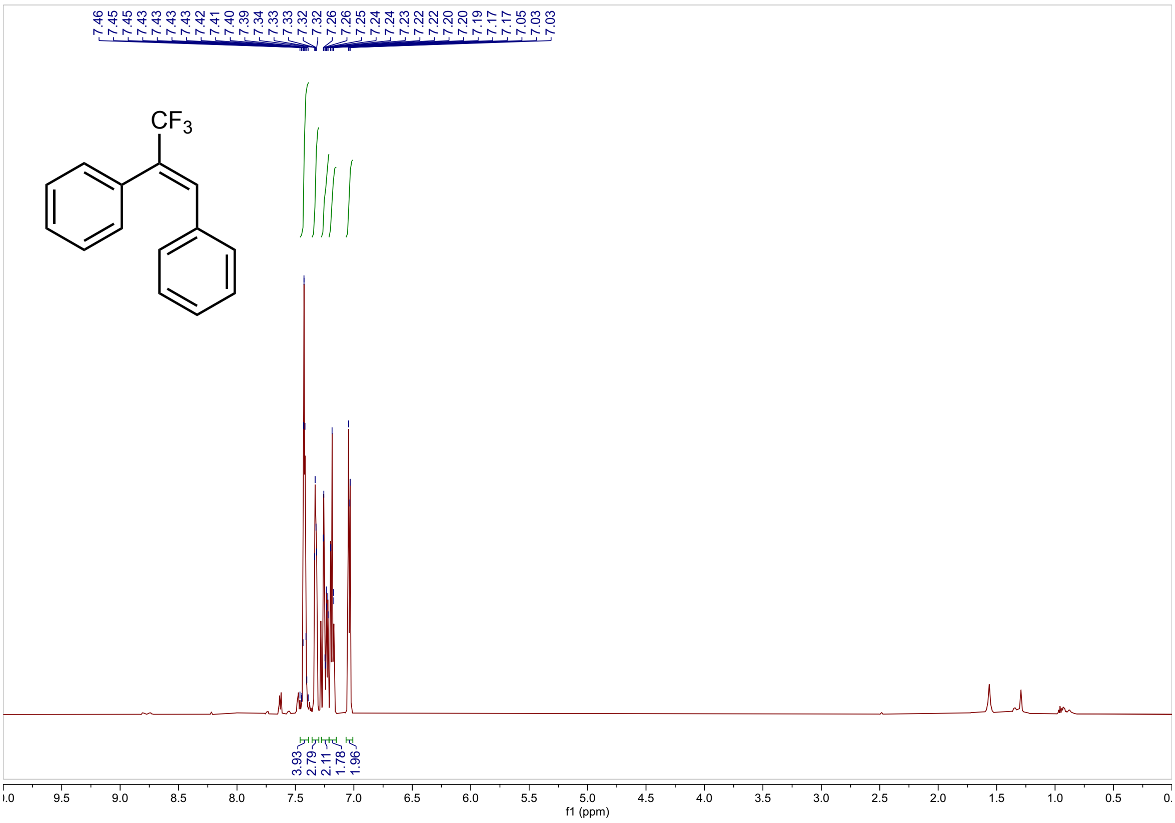

^{19}F NMR (565 MHz, Chloroform-*d*)

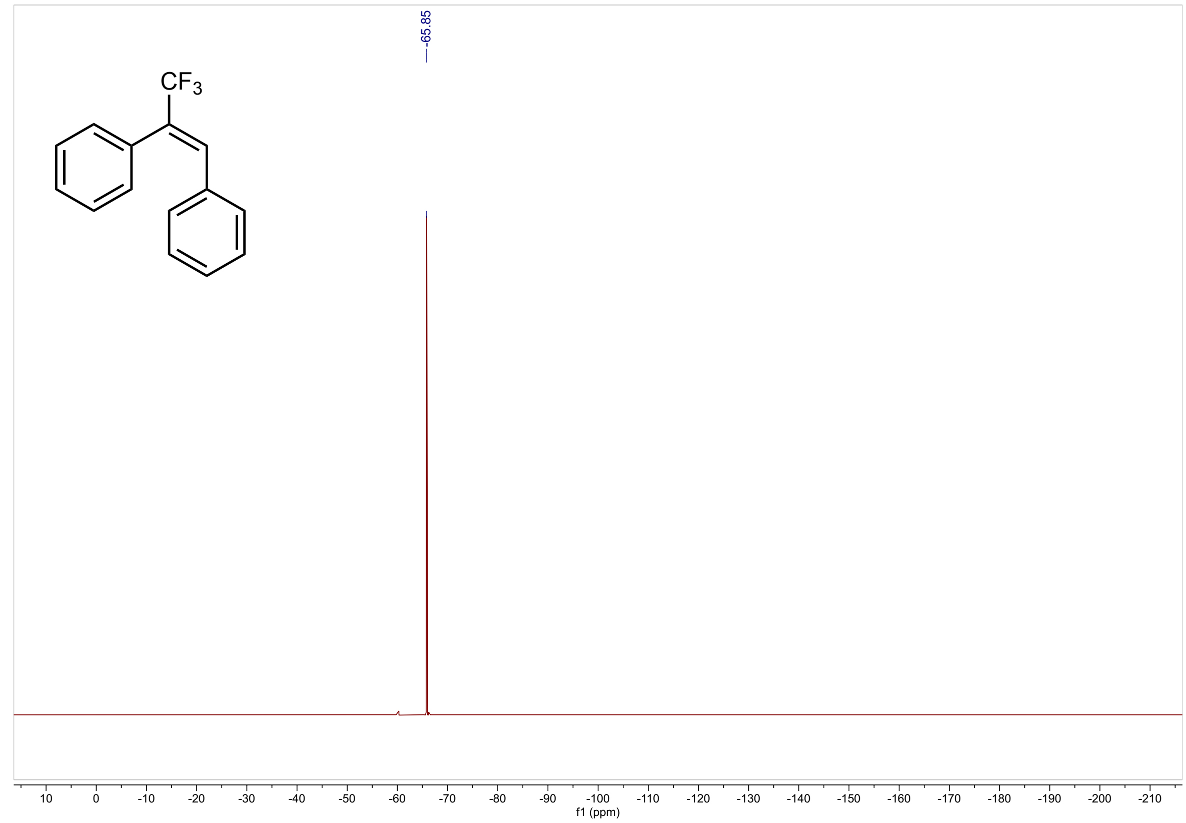

^{13}C NMR{^{1}H, ^{19}F} (151 MHz, Chloroform-*d*)

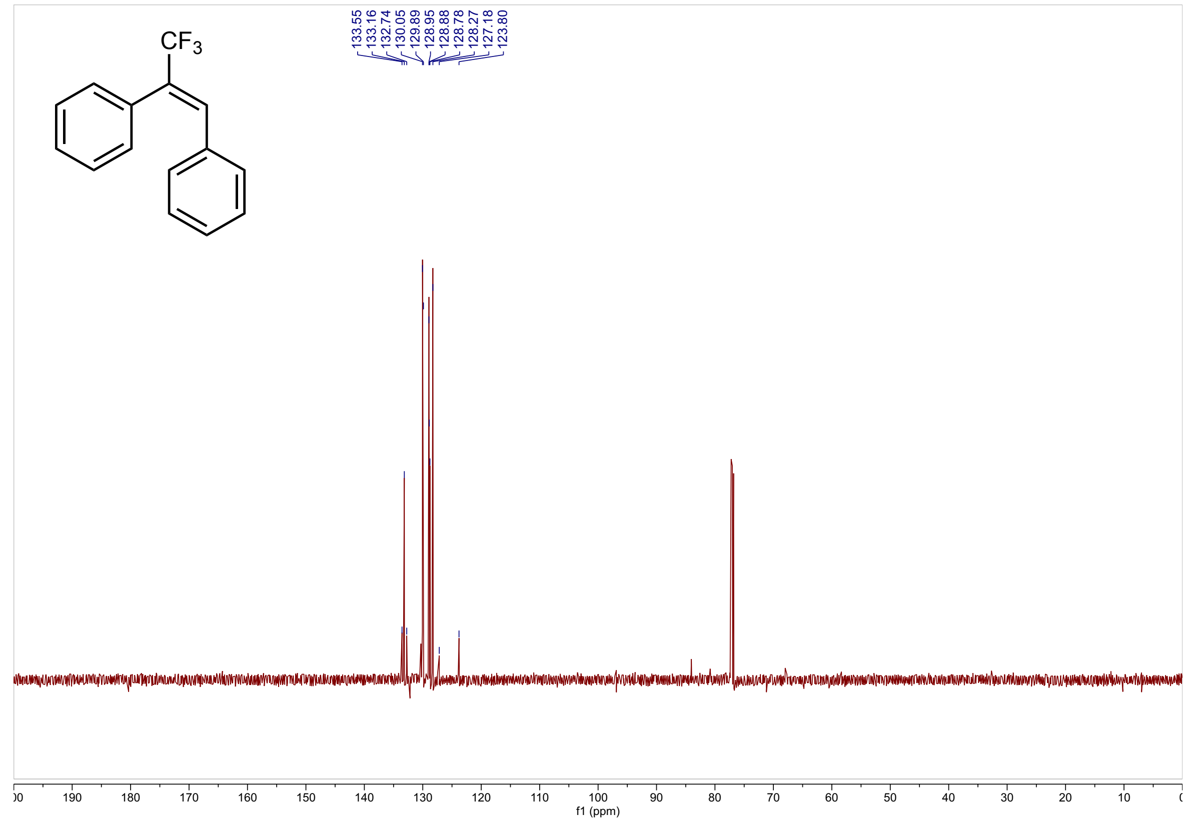

^{13}C NMR{^{1}H} (126 MHz, Chloroform-*d*)

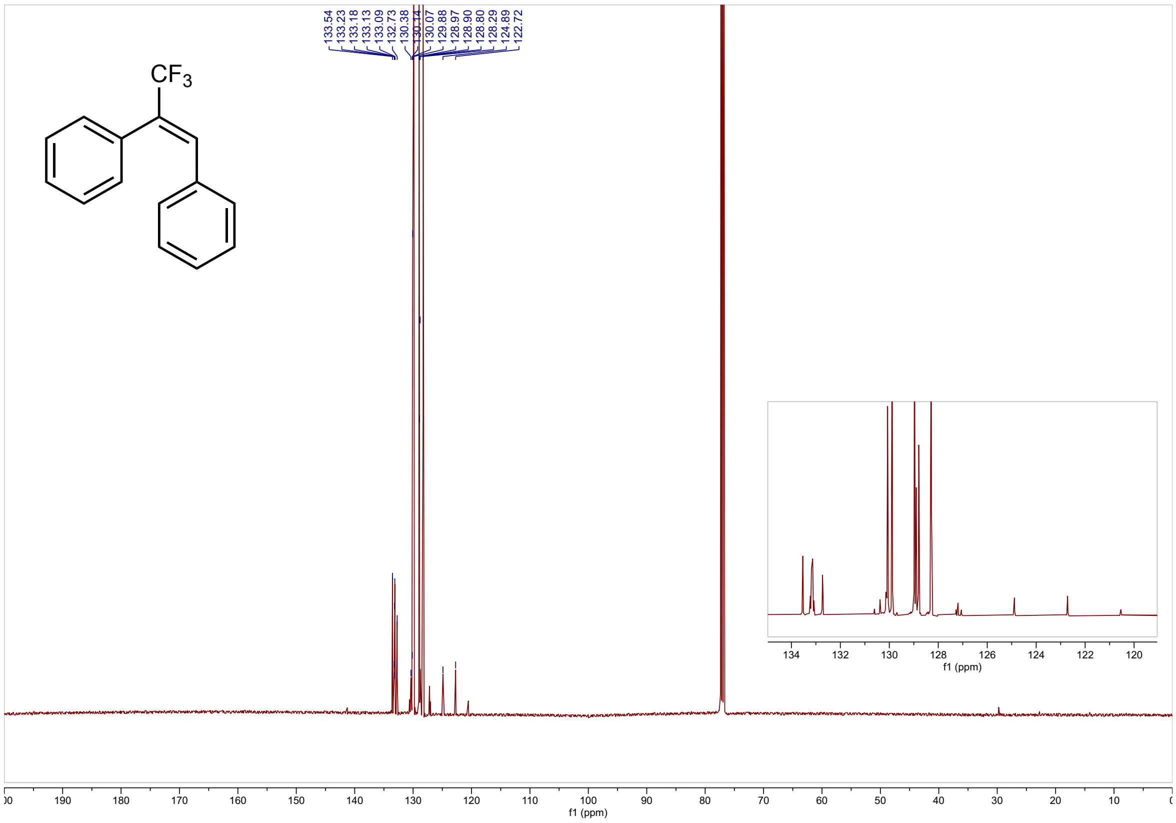

(*E*)-1-Methyl-4-(3,3,3-trifluoro-2-phenylprop-1-en-1-yl)benzene (9c)

^{1}H NMR (600 MHz, Chloroform-*d*)

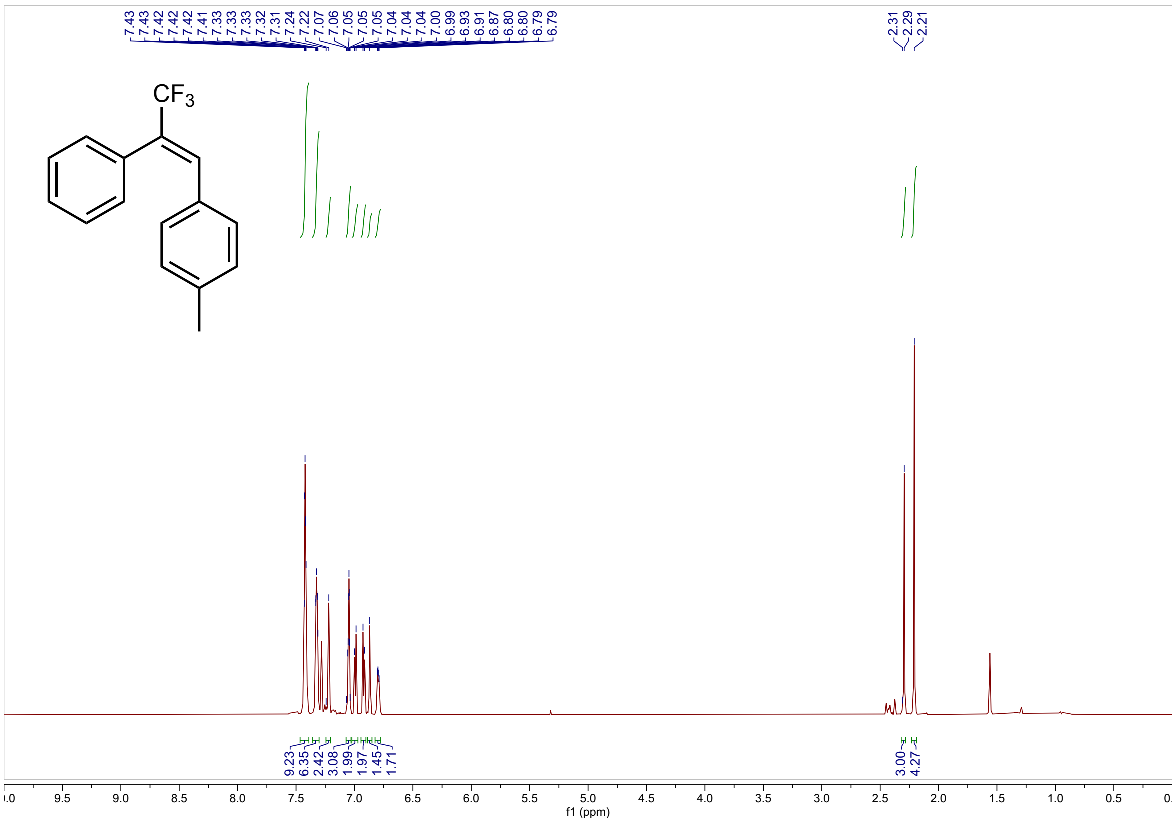

^{19}F NMR (565 MHz, Chloroform-*d*)

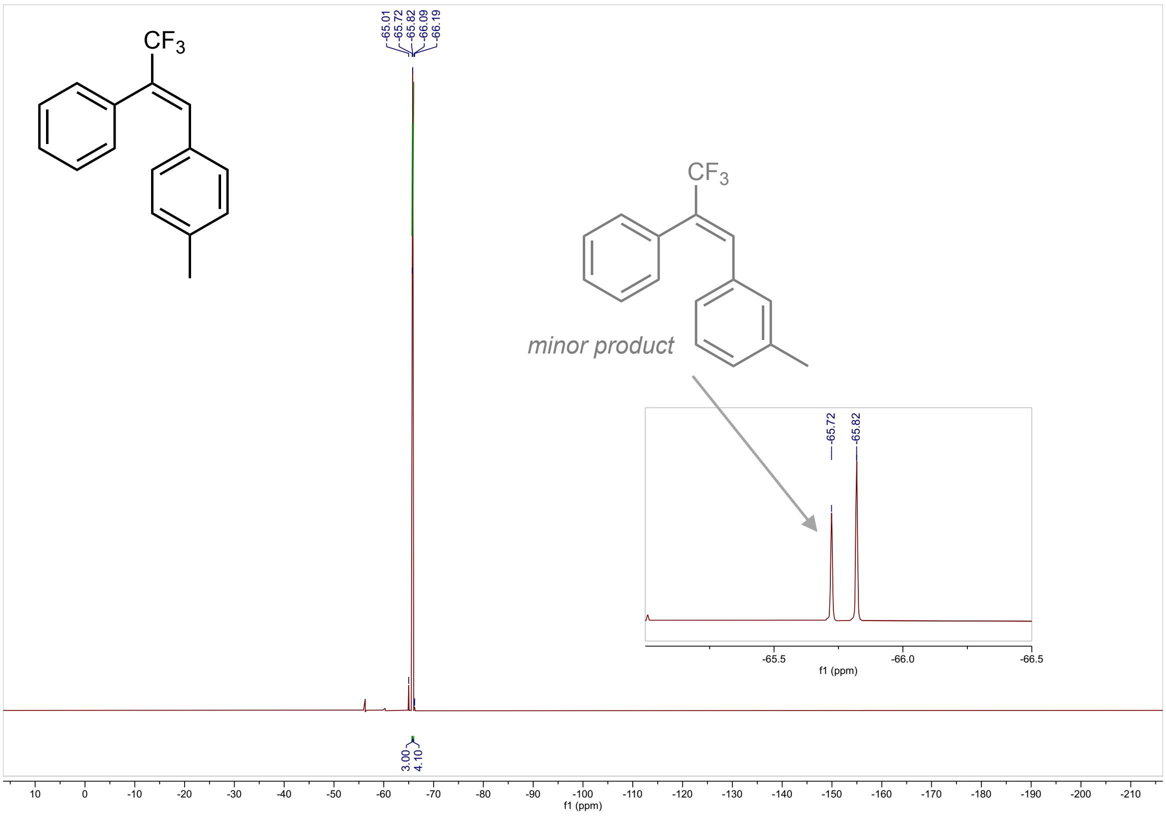

^{13}C NMR{^{1}H, ^{19}F} (151 MHz, Chloroform-*d*)

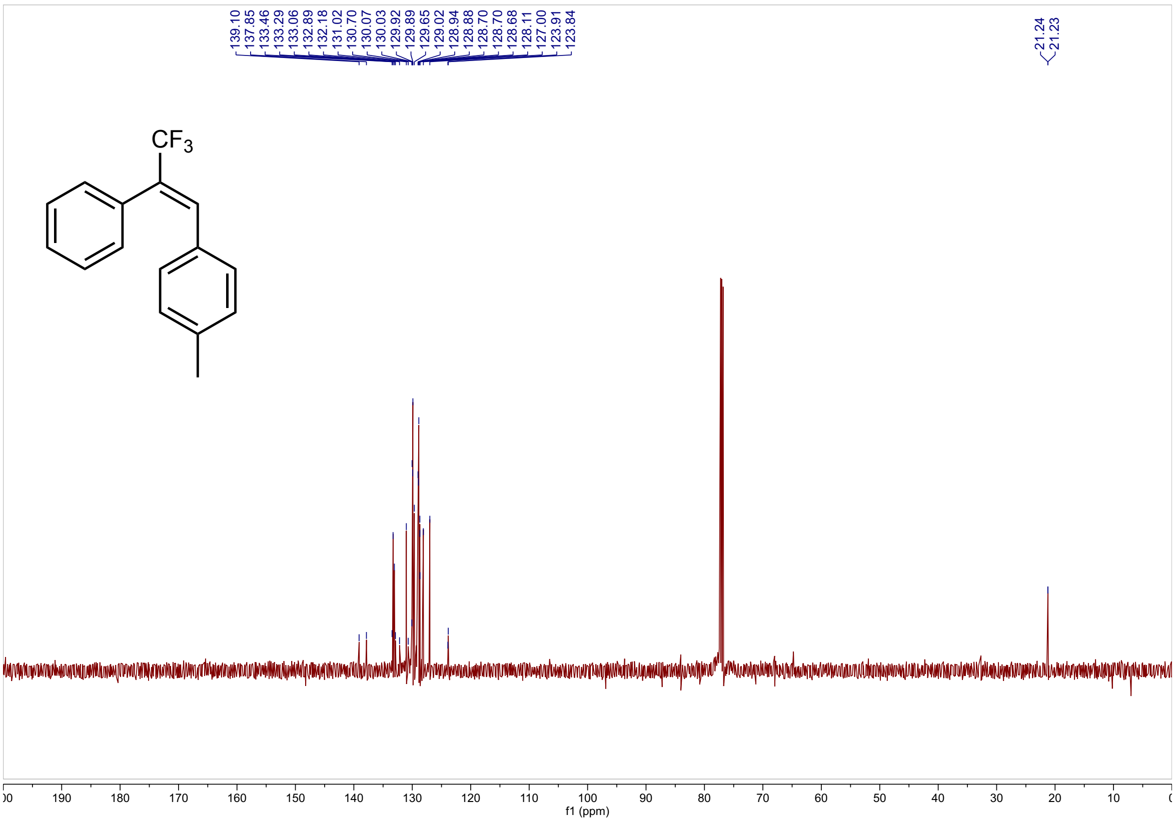

^{13}C NMR{^{1}H} (126 MHz, Chloroform-*d*)

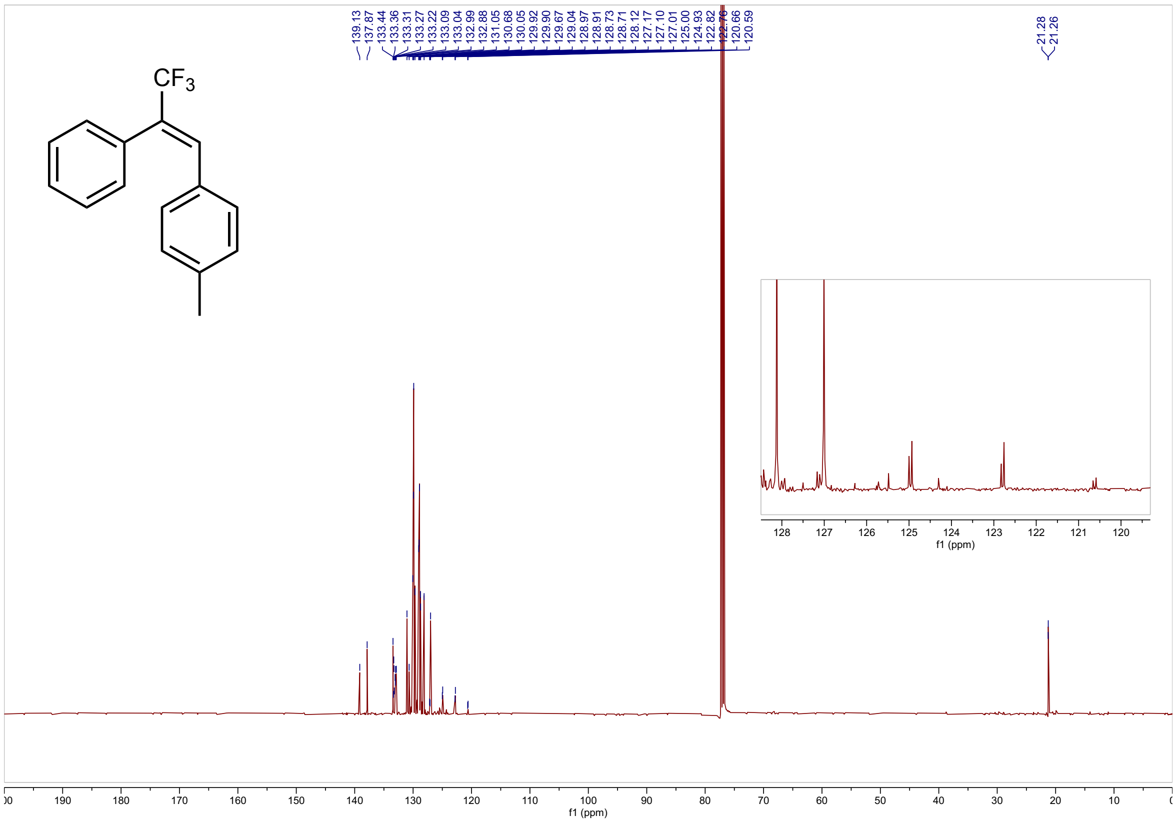

(*E*)-1,2-Dimethyl-4-(3,3,3-trifluoro-2-phenylprop-1-en-1-yl)benzene (9d)

^{1}H NMR (600 MHz, Chloroform-*d*)

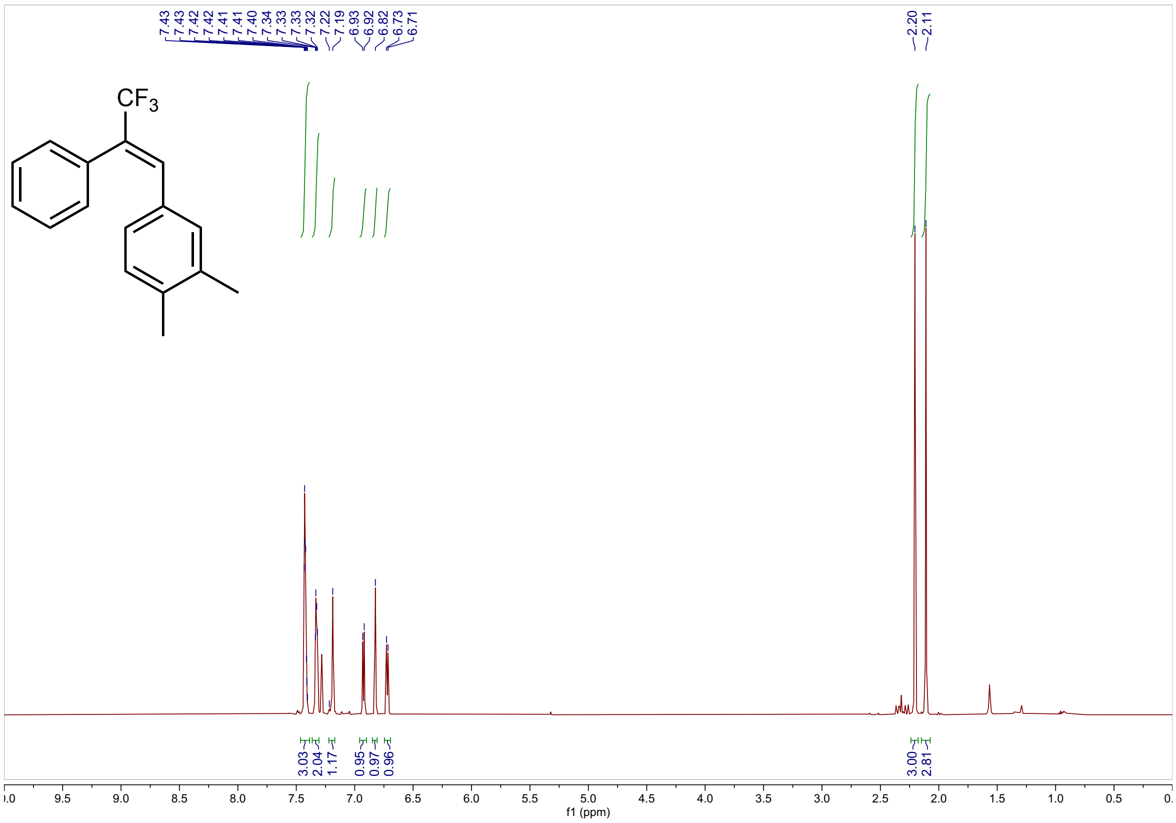

^{19}F NMR (565 MHz, Chloroform-*d*)

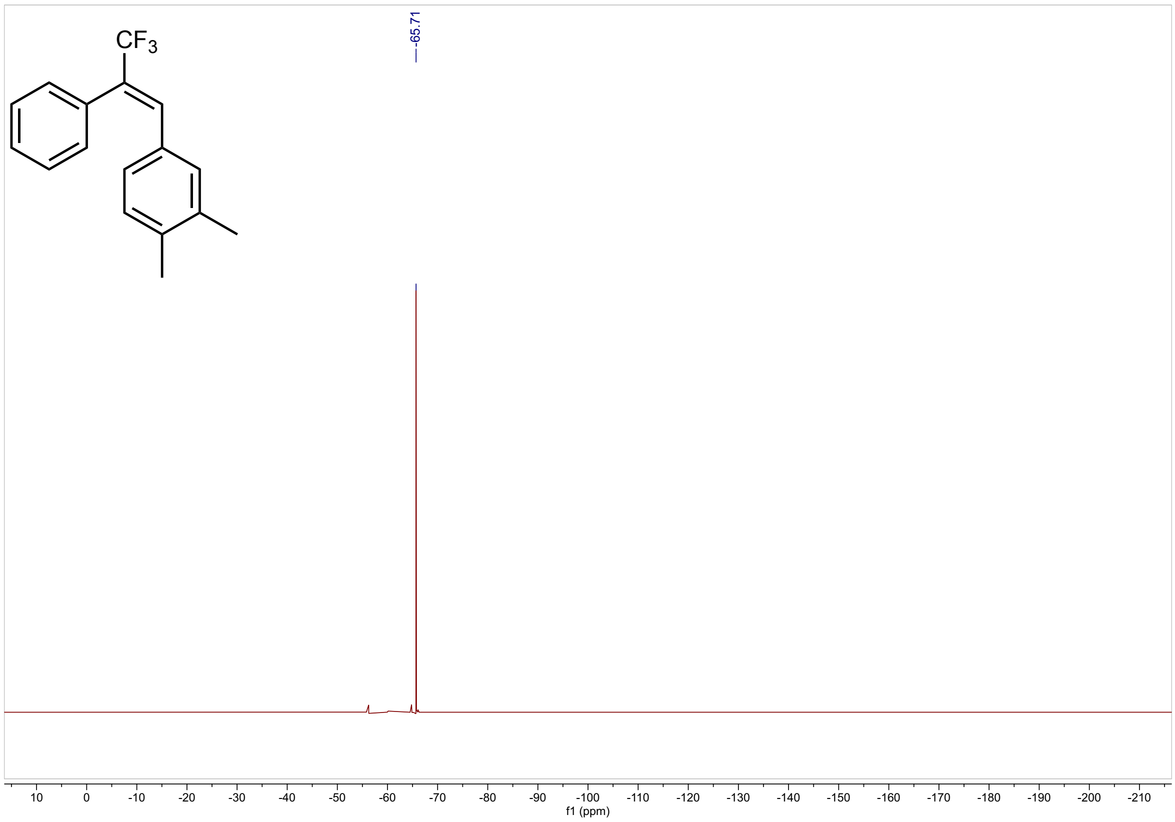

^{13}C NMR{^{1}H, ^{19}F} (151 MHz, Chloroform-*d*)

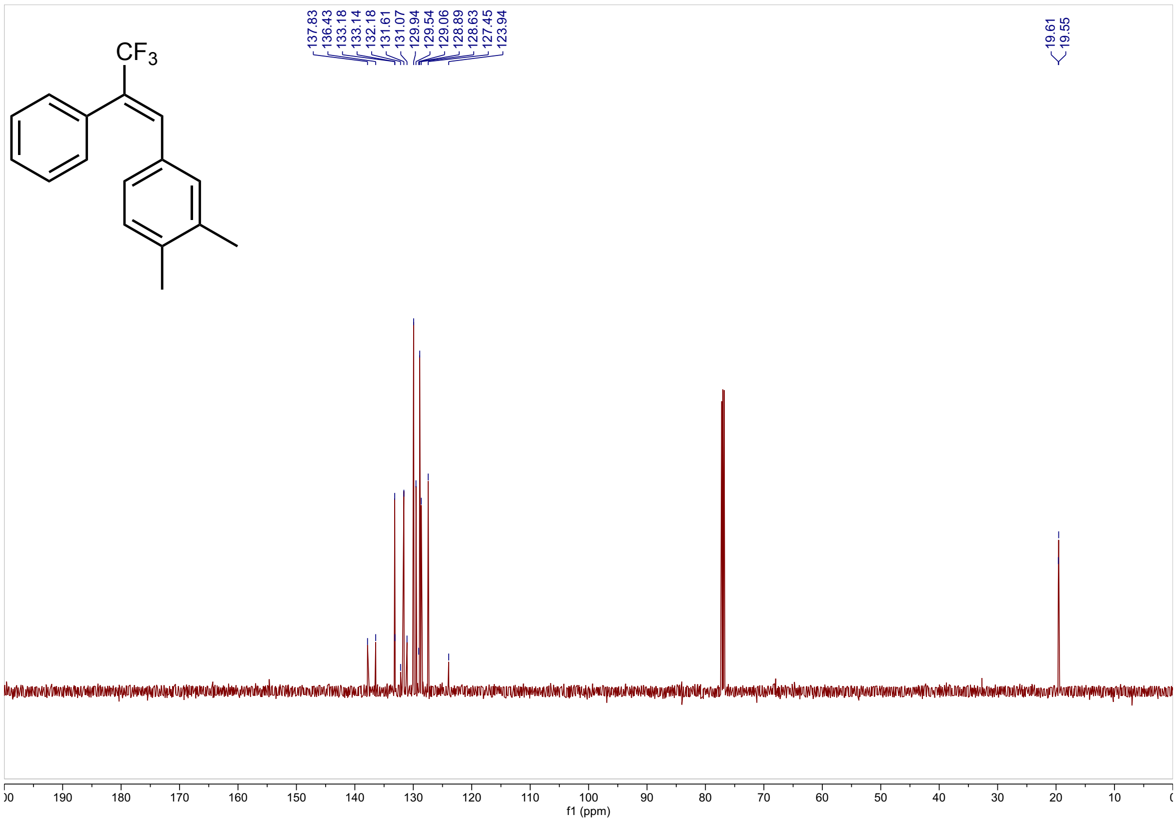

^{13}C NMR{^{1}H} (126 MHz, Chloroform-*d*)

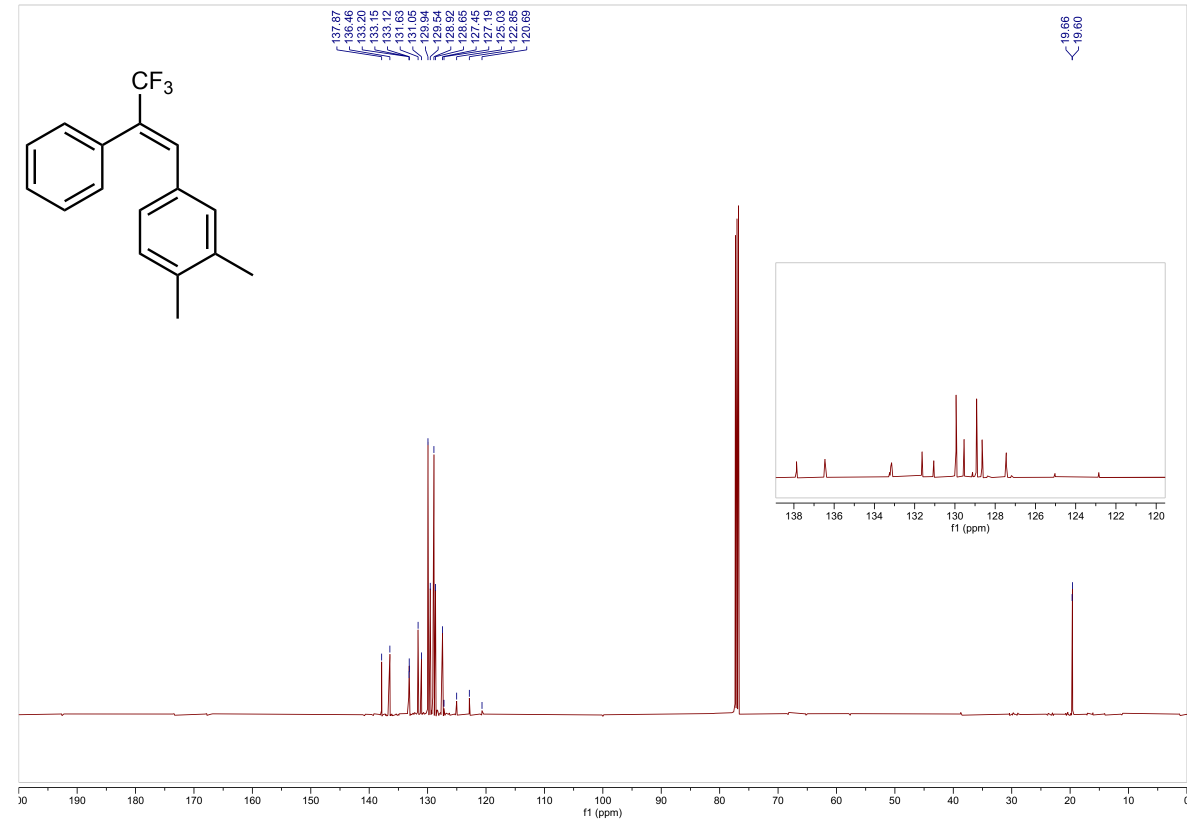

(*E*)-1,3-Dimethyl-5-(3,3,3-trifluoro-2-phenylprop-1-en-1-yl)benzene (9e)

^{1}H NMR (600 MHz, Chloroform-*d*)

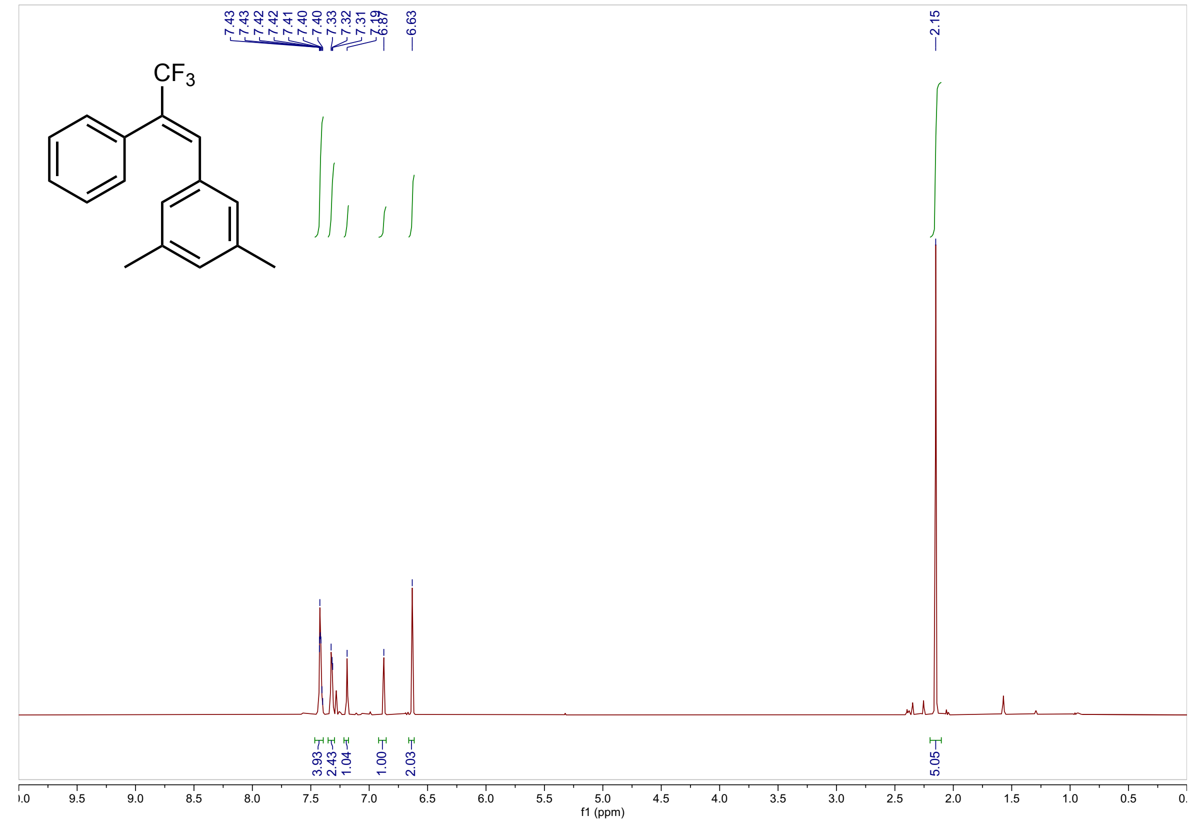

^{19}F NMR (565 MHz, Chloroform-*d*)

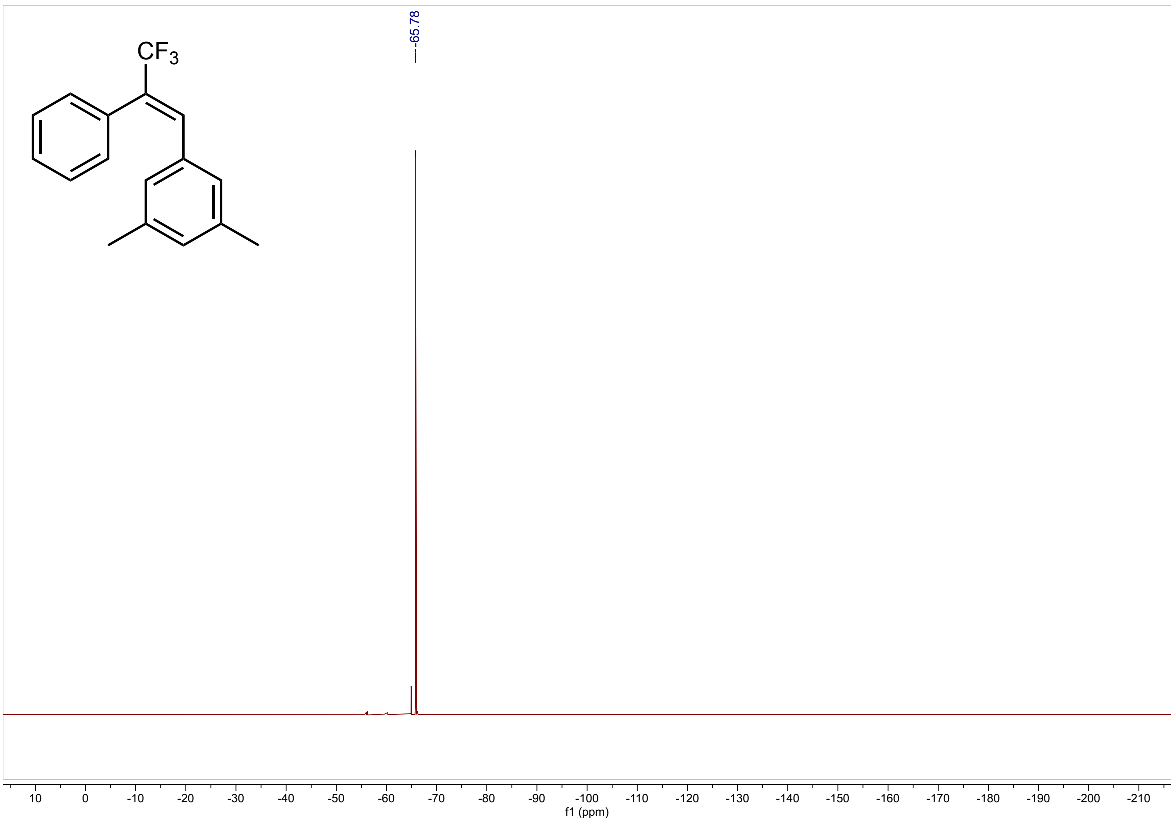

^{13}C NMR{^{1}H, ^{19}F} (151 MHz, Chloroform-*d*)

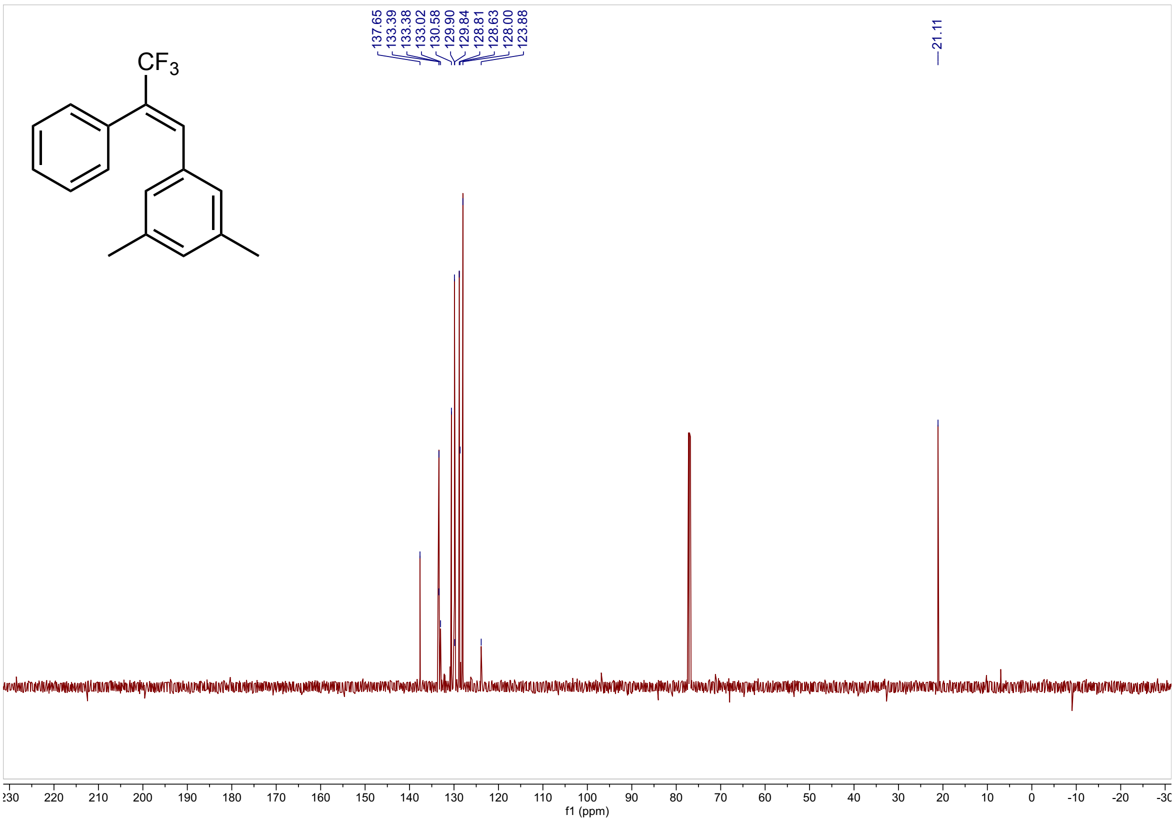

(*E*)-1,4-Dimethyl-2-(3,3,3-trifluoro-2-phenylprop-1-en-1-yl)benzene (9f)

^{1}H NMR (600 MHz, Chloroform-*d*)

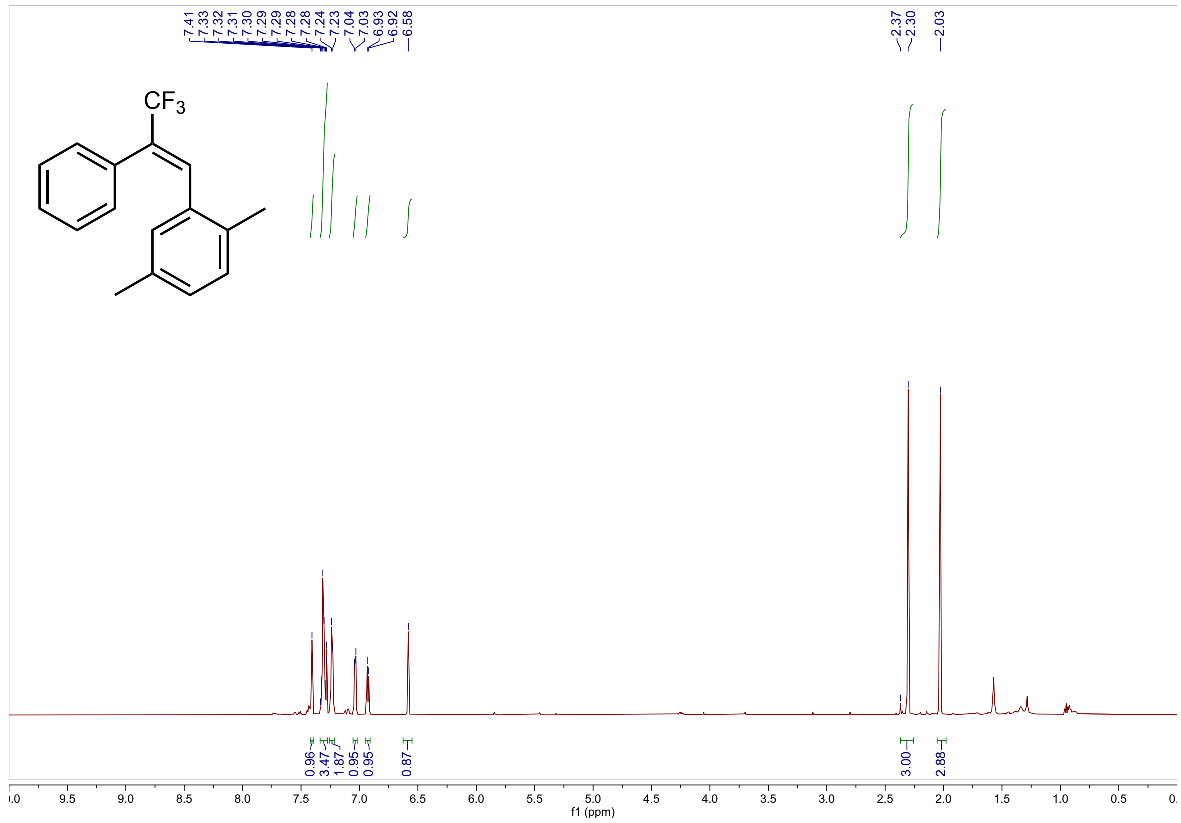

^{19}F NMR (565 MHz, Chloroform-*d*)

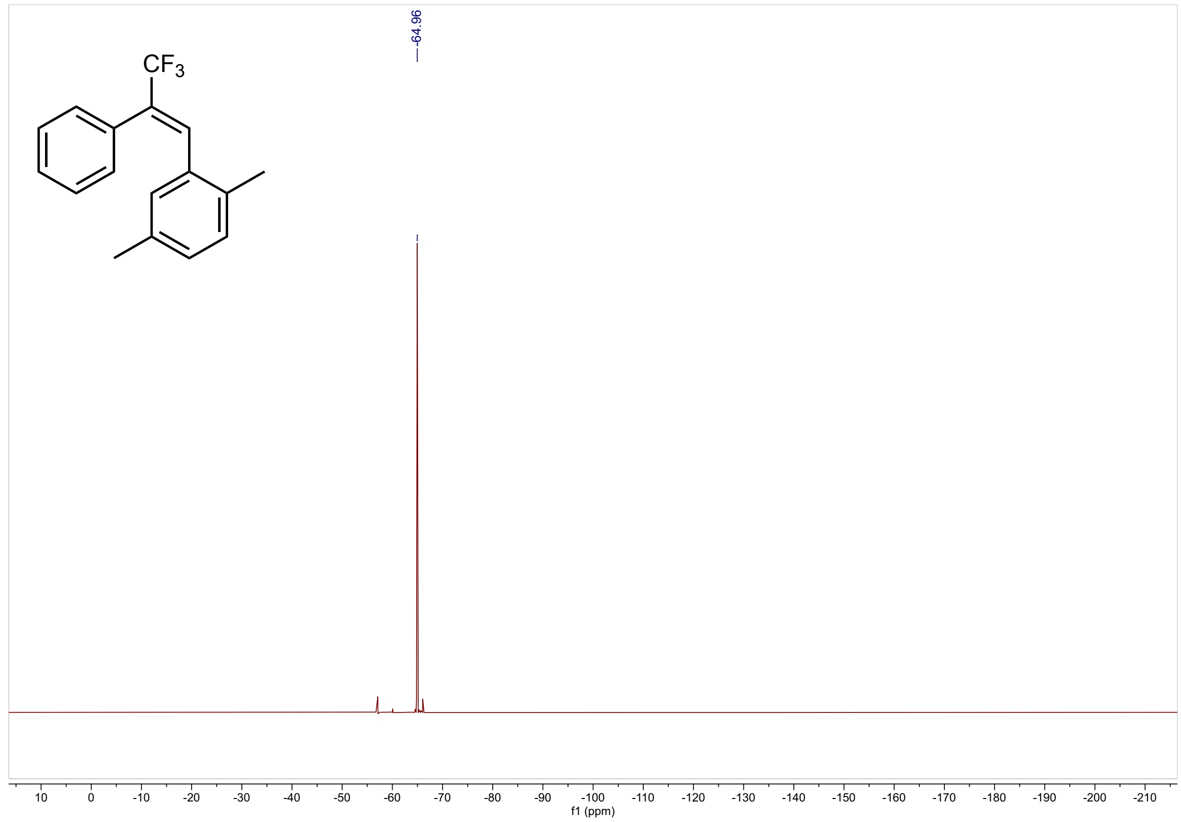

^{13}C NMR{^{1}H, ^{19}F} (151 MHz, Chloroform-*d*)

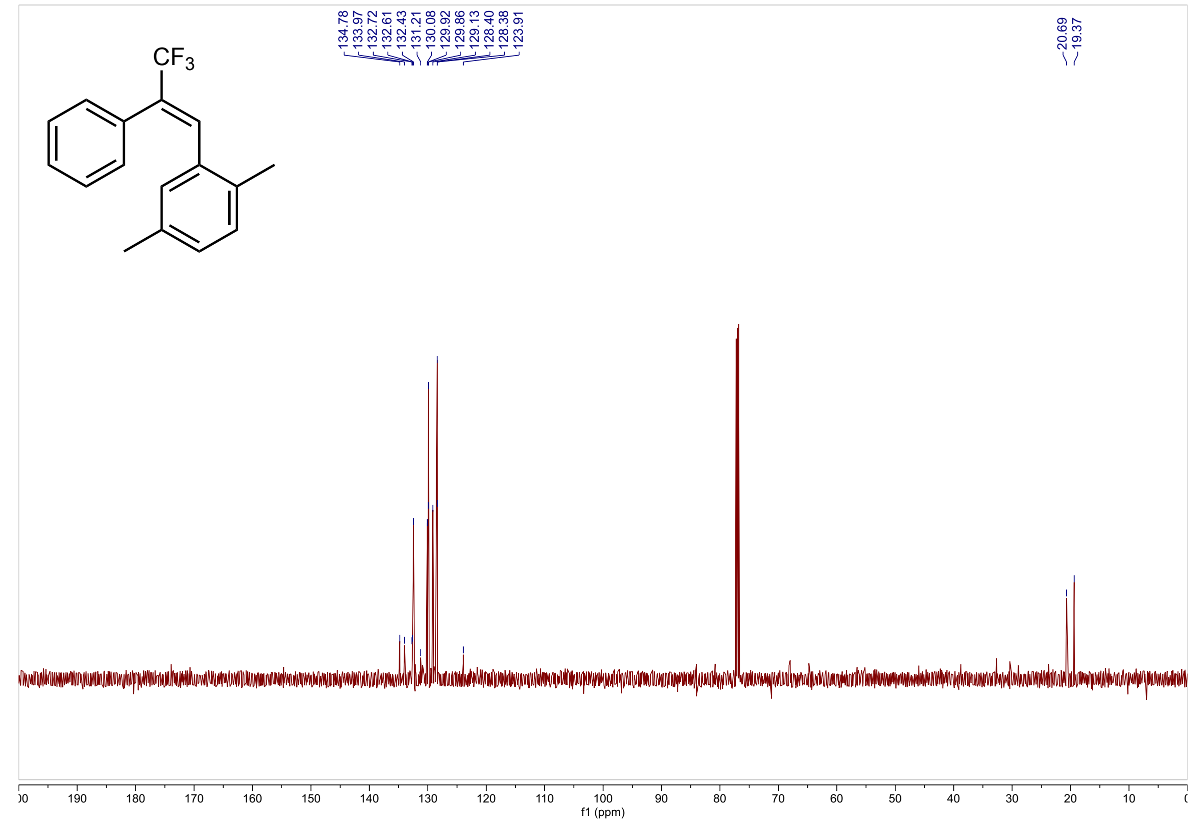

(*E*)-1-Methoxy-4-(3,3,3-trifluoro-1-phenylprop-1-en-2-yl)benzene (11a)

^{1}H NMR (600 MHz, Chloroform-*d*)

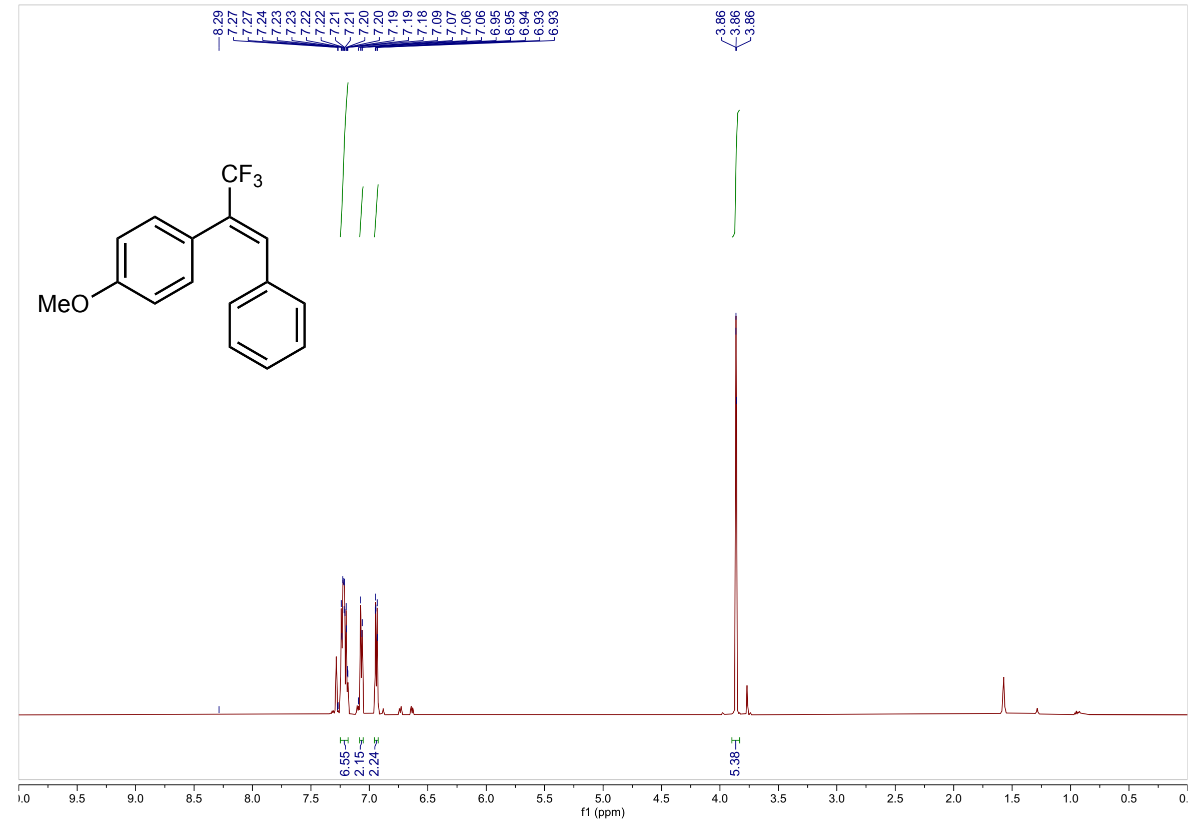

^{19}F NMR (565 MHz, Chloroform-*d*)

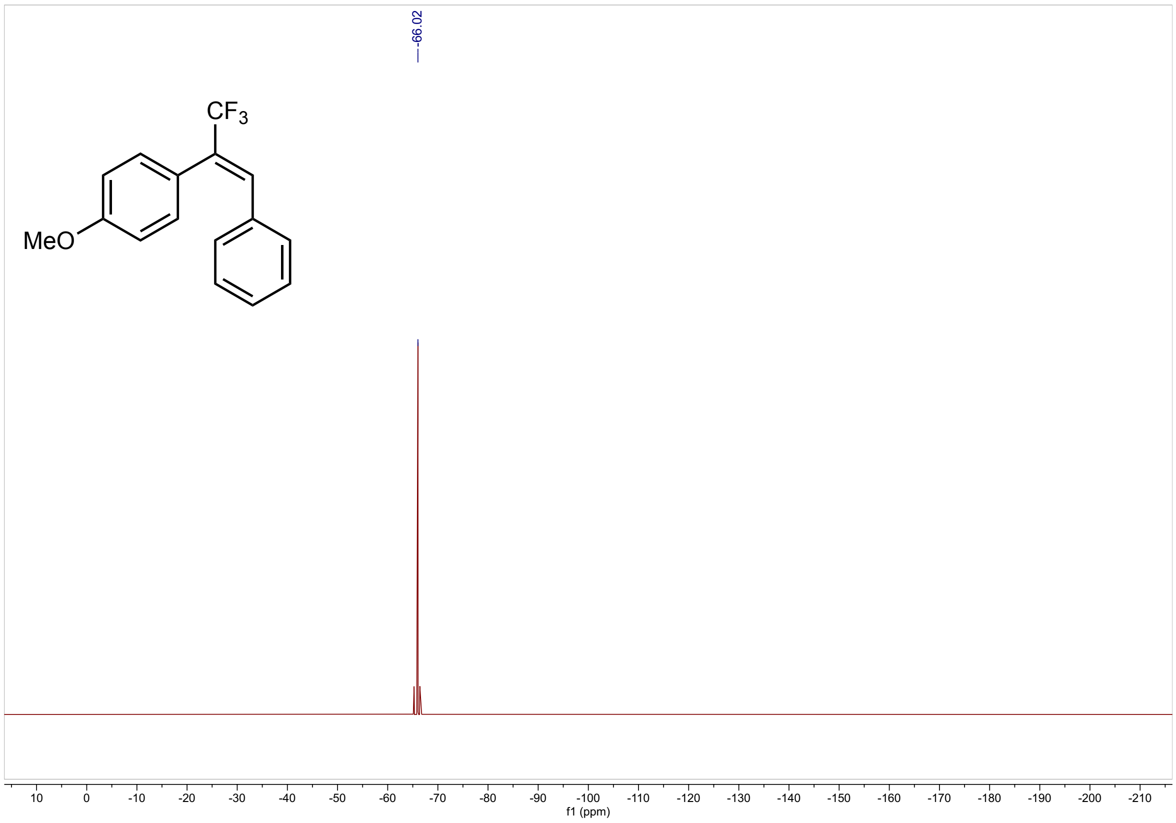

^{13}C NMR{^{1}H, ^{19}F} (151 MHz, Chloroform-*d*)

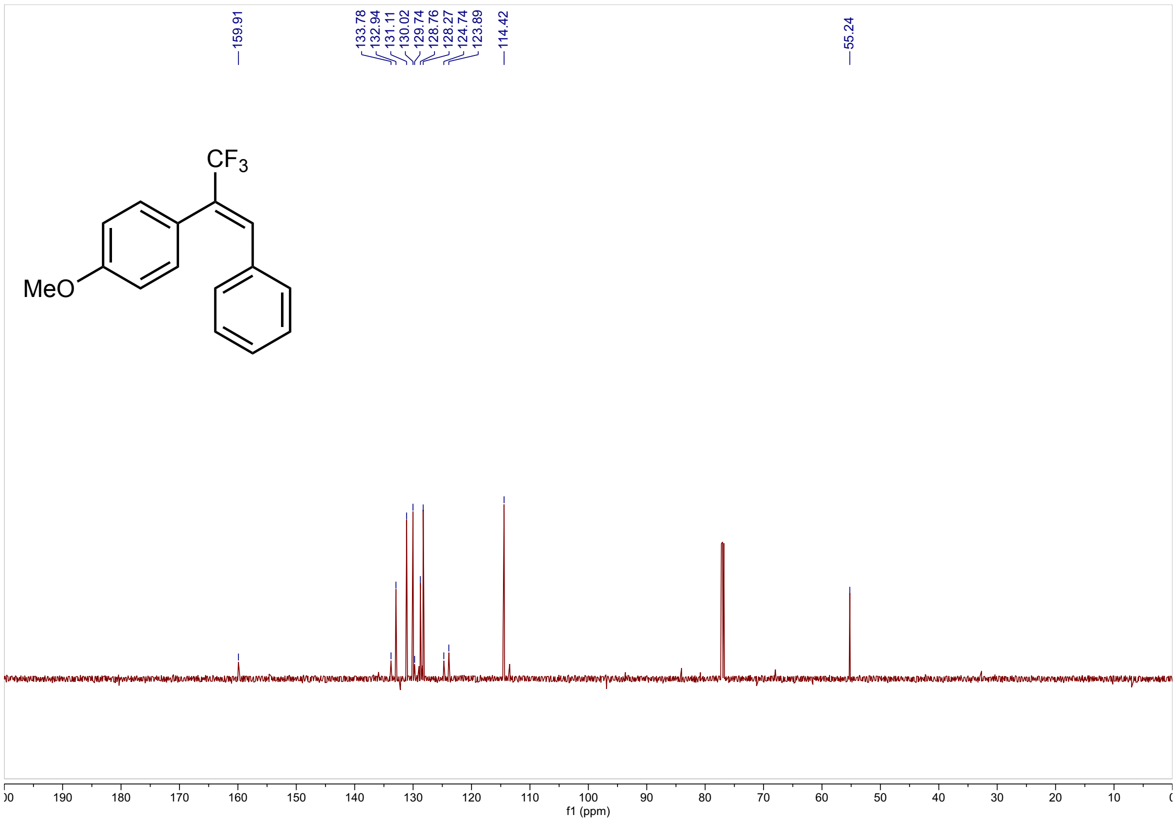

(*E*)-1-chloro-4-(3,3,3-trifluoro-1-phenylprop-1-en-2-yl)benzene (11b)

^{1}H NMR (600 MHz, Chloroform-*d*)

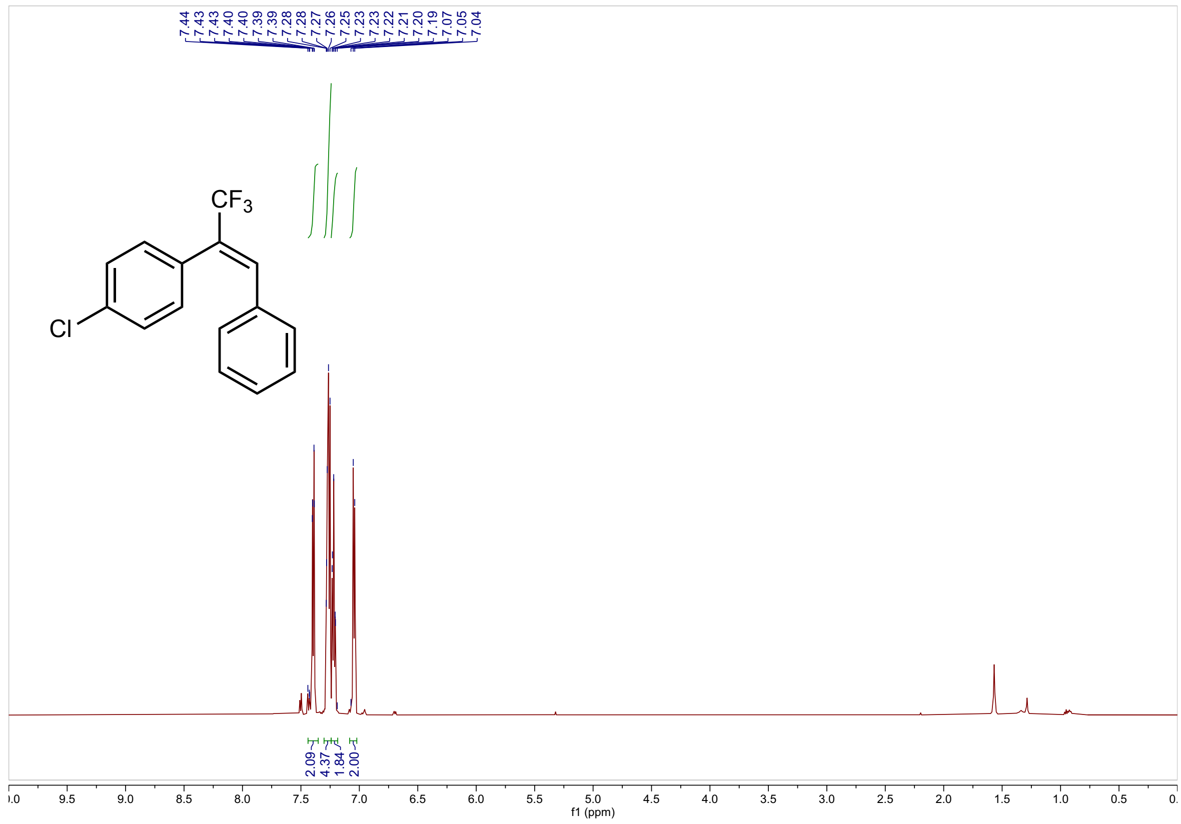

^{19}F NMR (565 MHz, Chloroform-*d*)

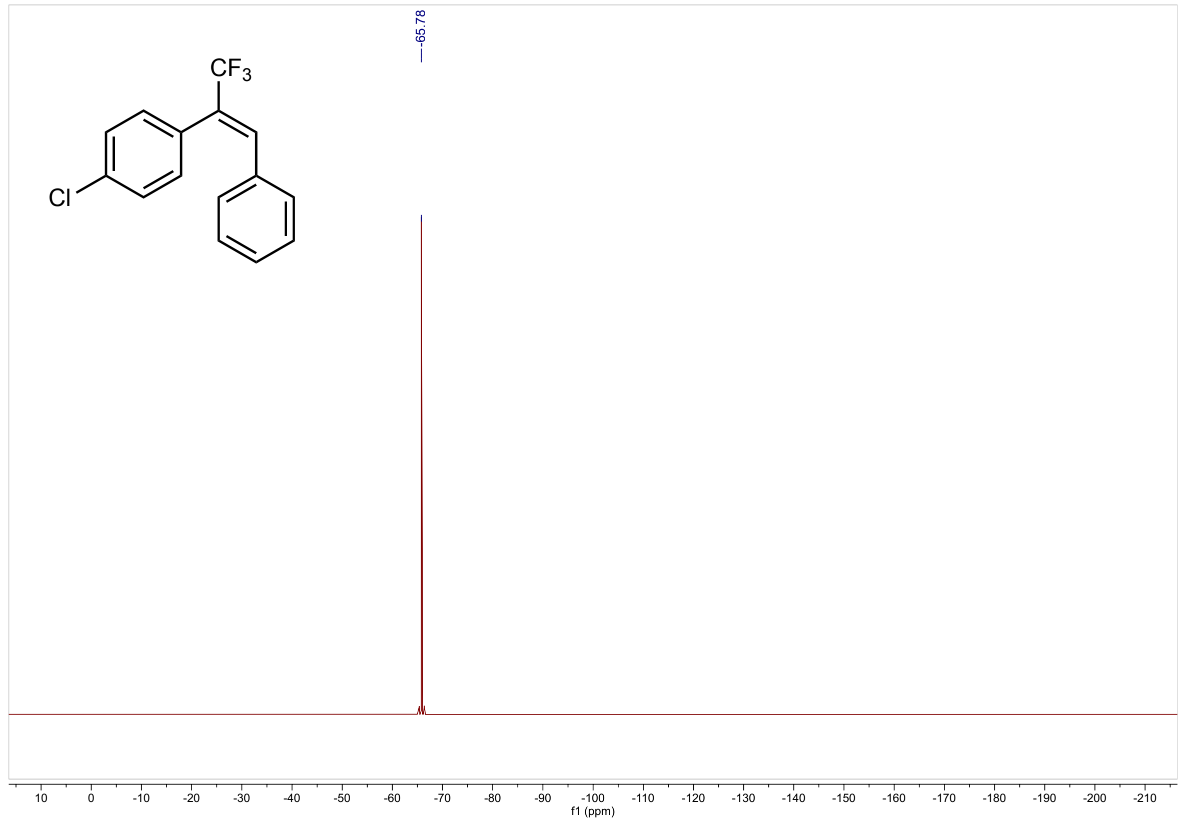

^{13}C NMR{^{1}H, ^{19}F} (151 MHz, Chloroform-*d*)

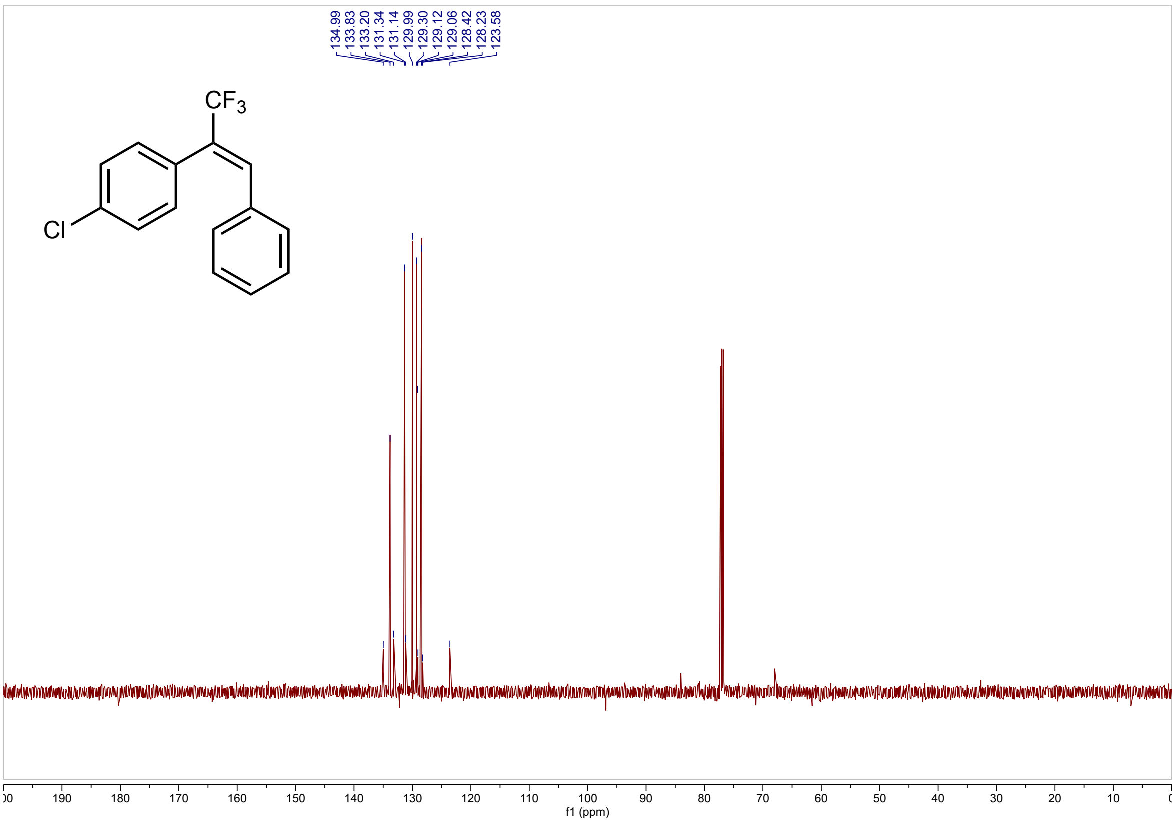

(*E*)-1-Nitro-4-(3,3,3-trifluoro-1-phenylprop-1-en-2-yl)benzene (11c)

^{1}H NMR (600 MHz, Chloroform-*d*)

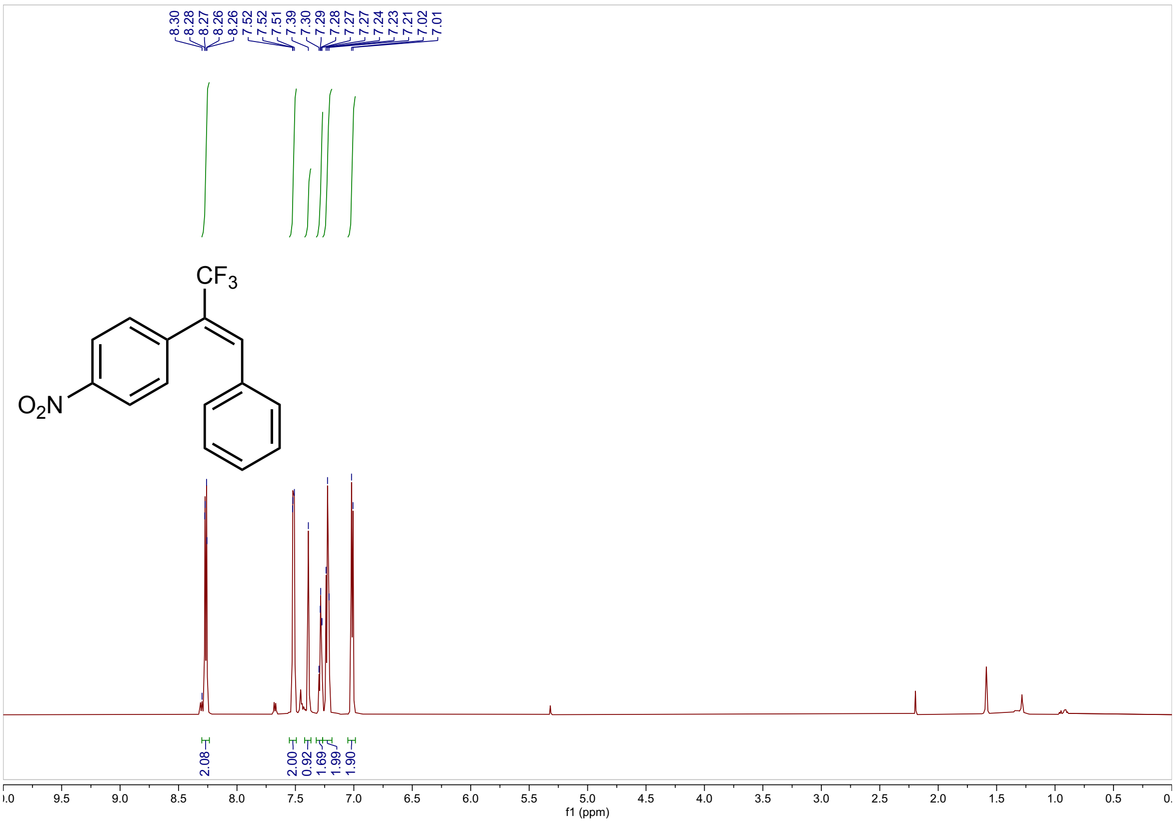

^{19}F NMR (565 MHz, Chloroform-*d*)

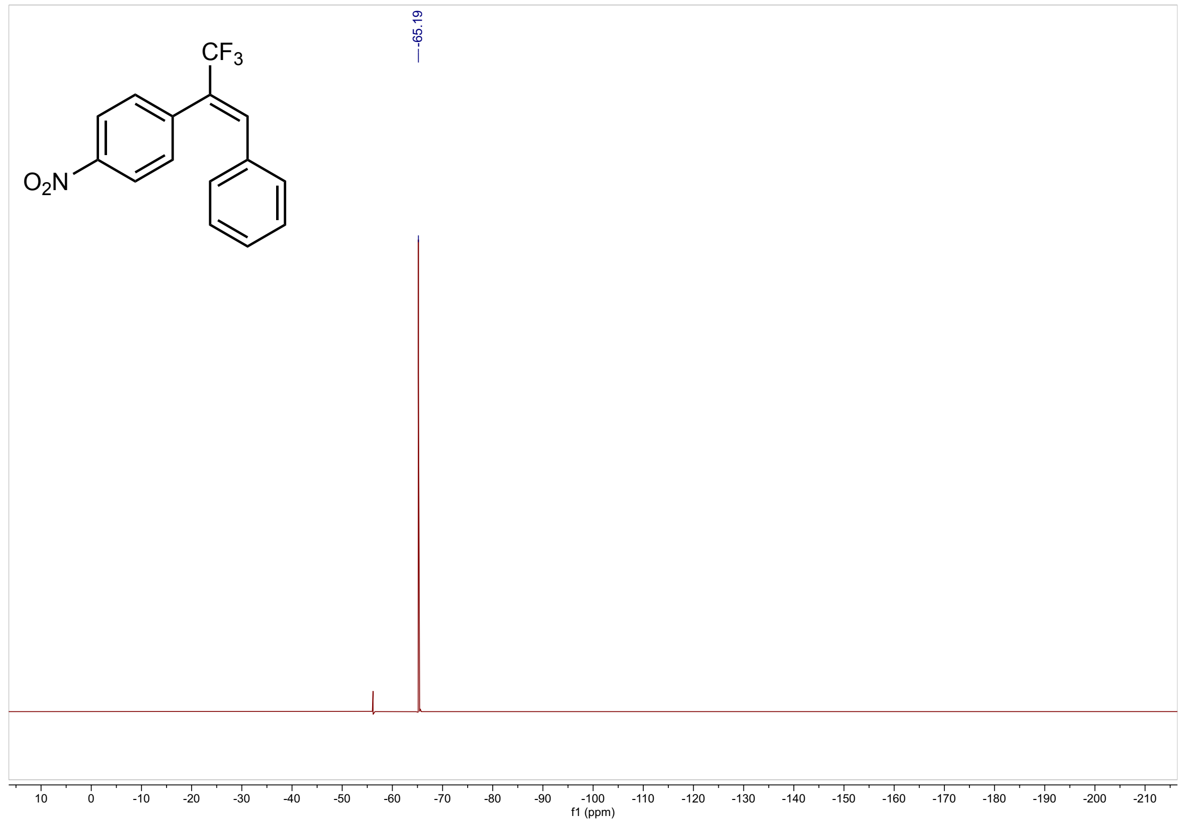

^{13}C NMR{^{1}H, ^{19}F} (151 MHz, Chloroform-*d*)

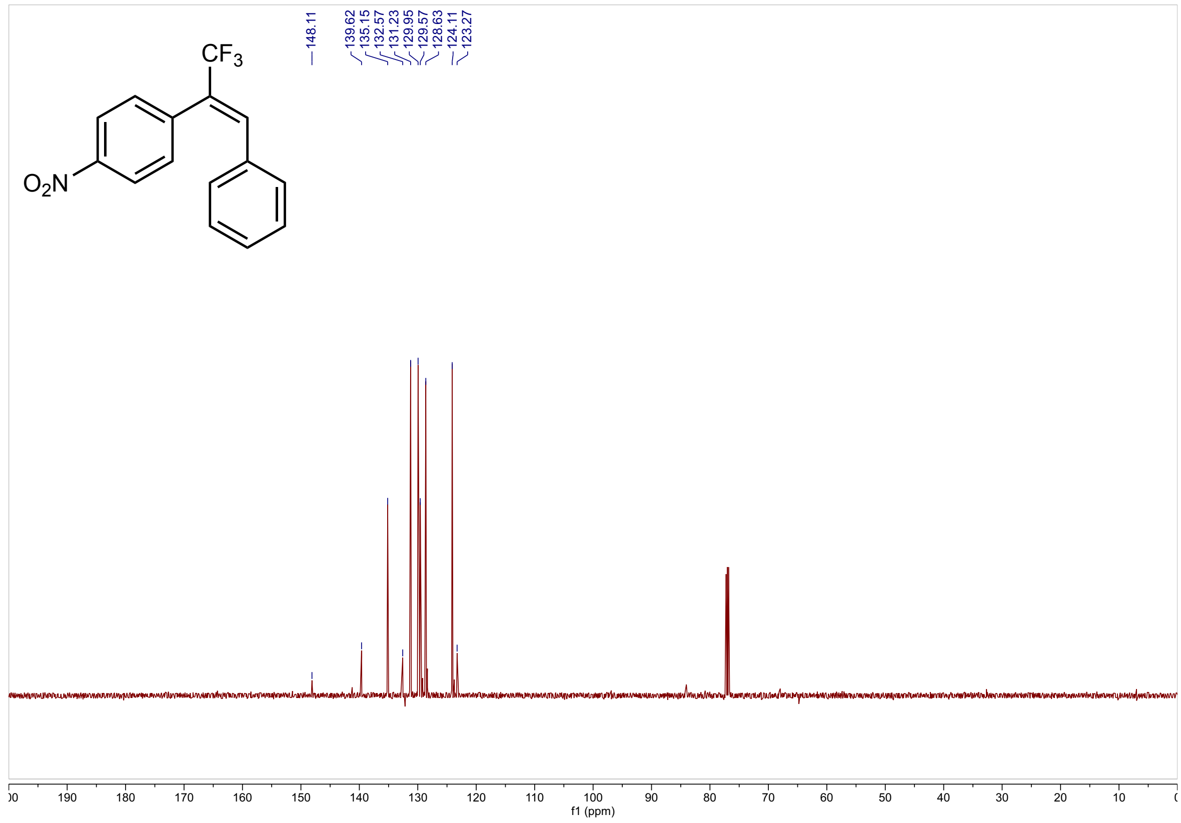

^{13}C NMR{^{1}H} (126 MHz, Chloroform-*d*)

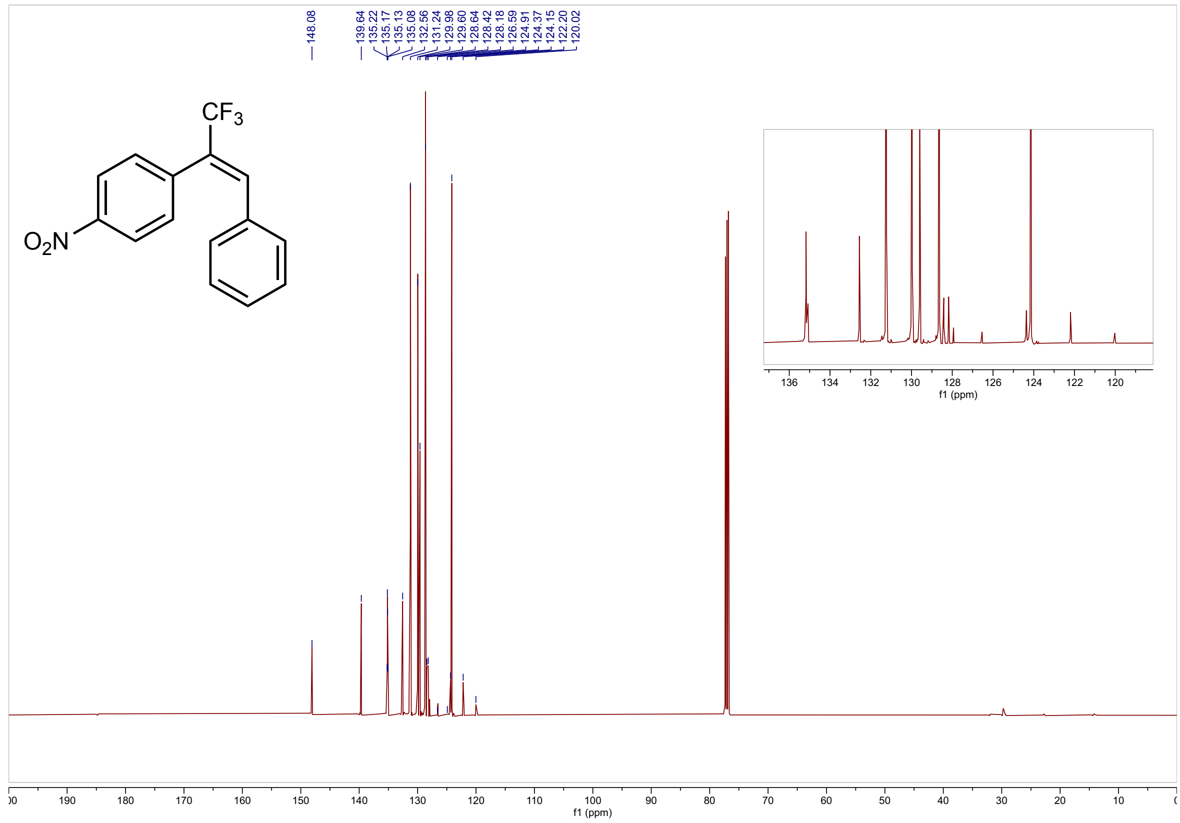

(*E*)-1-Chloro-3-(3,3,3-trifluoro-1-phenylprop-1-en-2-yl)benzene (11d)

^{1}H NMR (600 MHz, Chloroform-*d*)

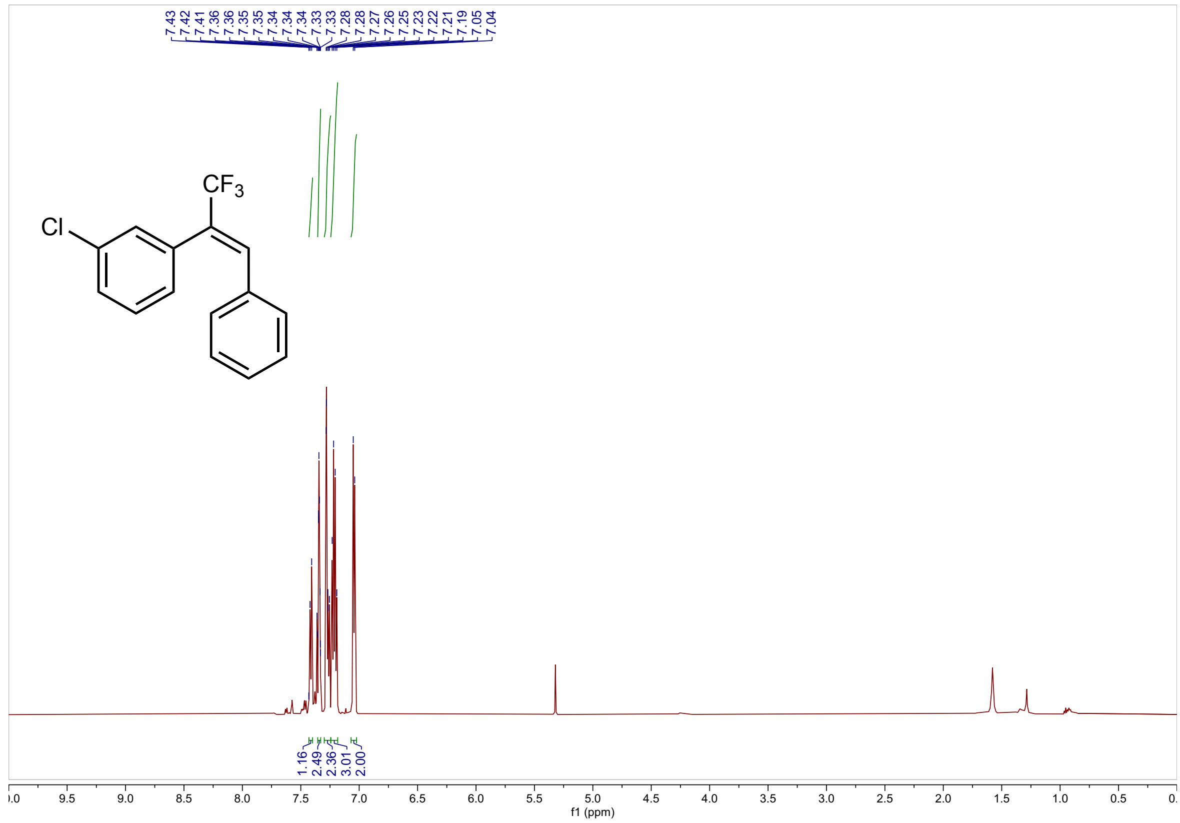

^{19}F NMR (565 MHz, Chloroform-*d*)

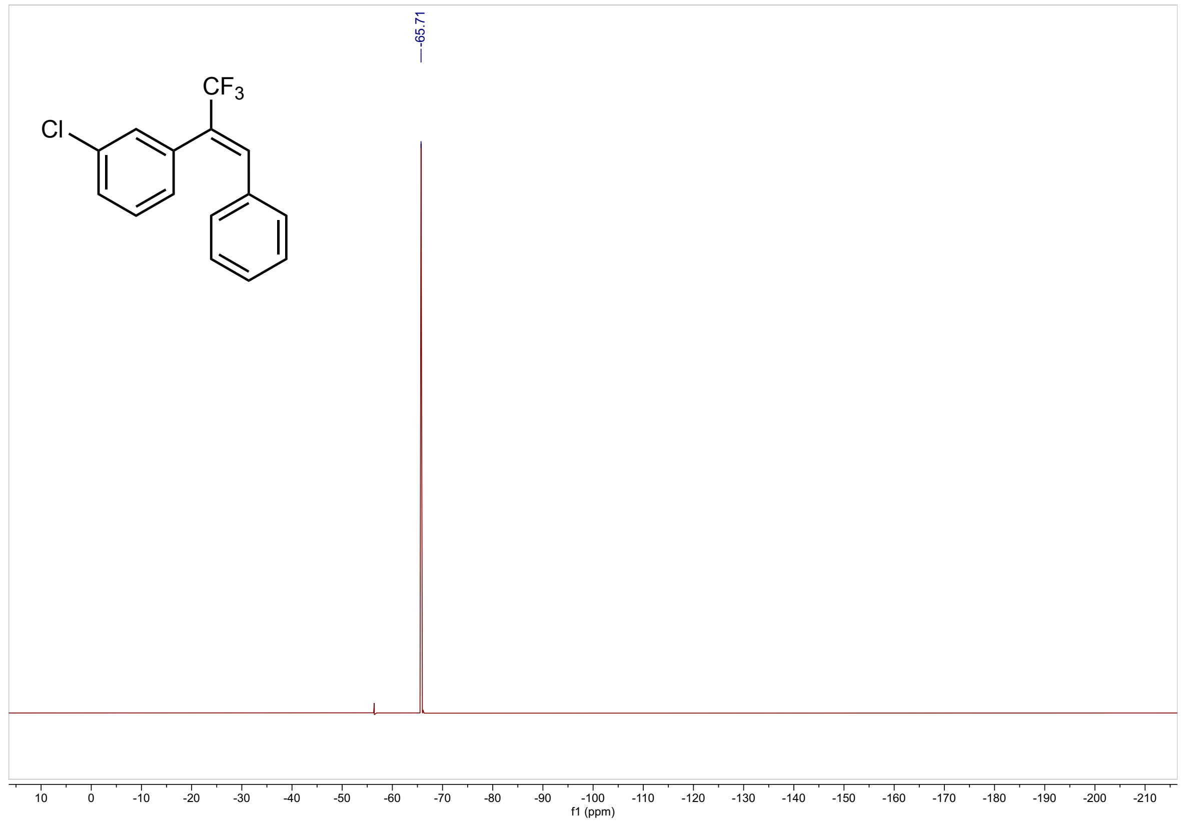

^{13}C NMR{^{1}H, ^{19}F} (151 MHz, Chloroform-*d*)

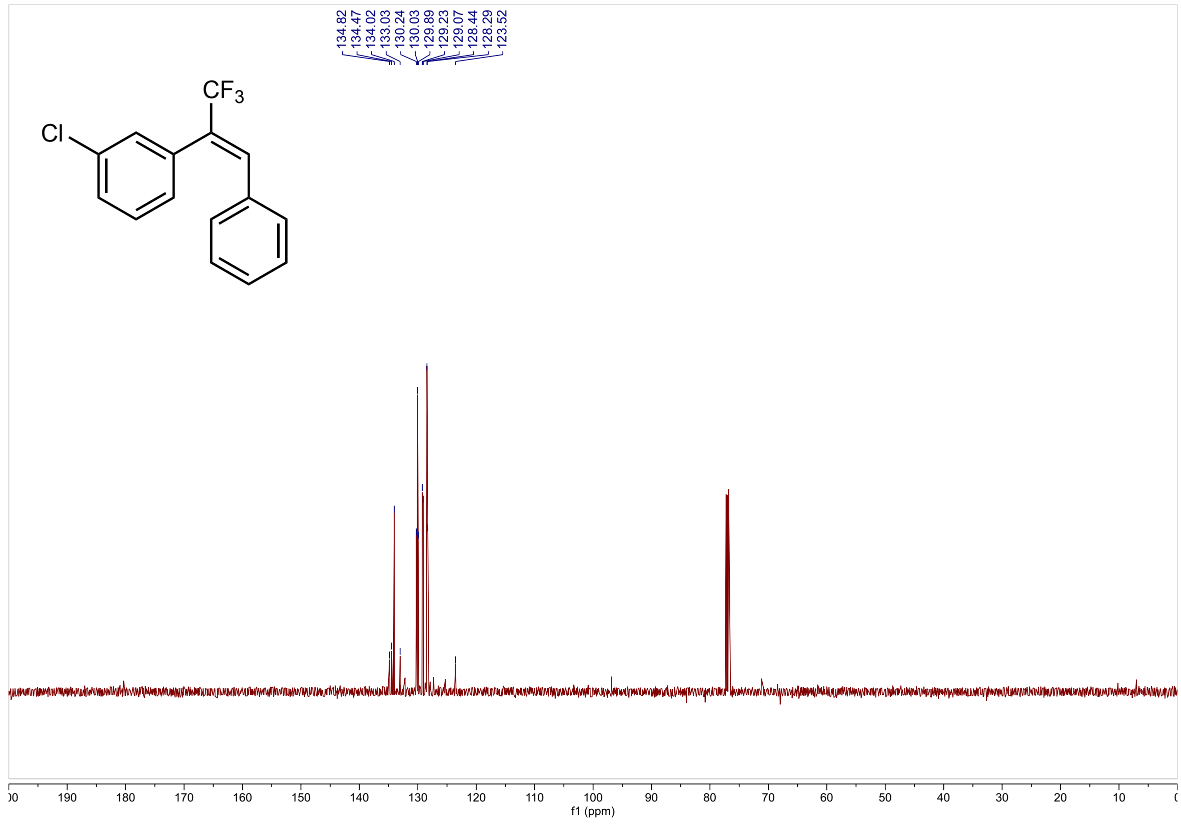

(*E*)-3-(3,3,3-Trifluoro-1-phenylprop-1-en-2-yl)benzonitrile (11e)

^{1}H NMR (600 MHz, Chloroform-*d*)

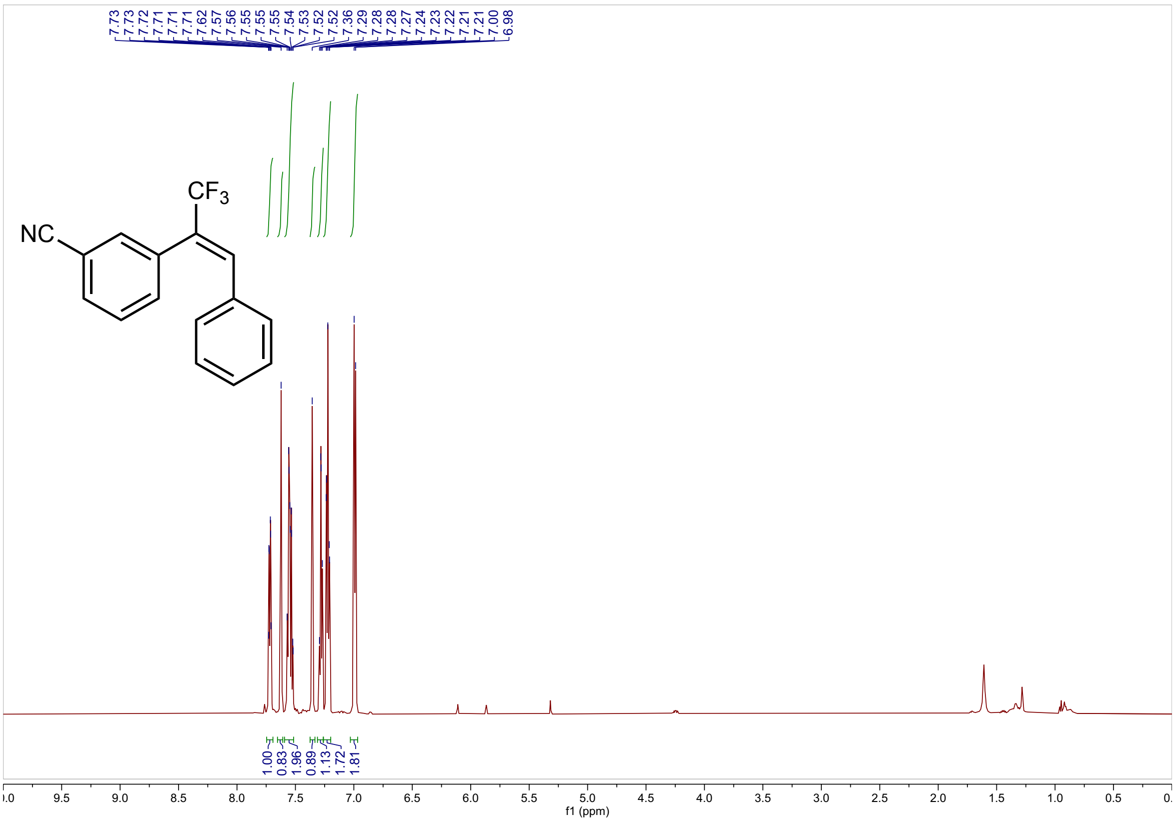

^{19}F NMR (565 MHz, Chloroform-*d*)

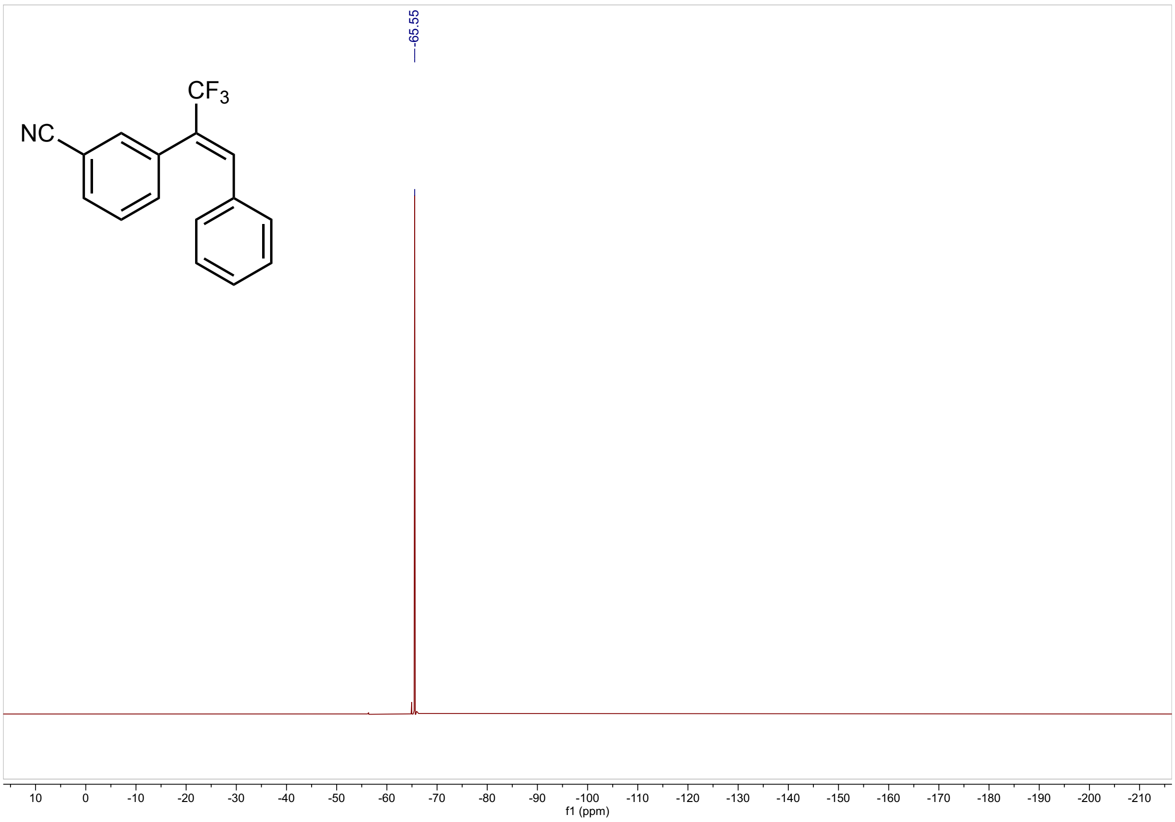

^{13}C NMR{^{1}H, ^{19}F} (151 MHz, Chloroform-*d*)

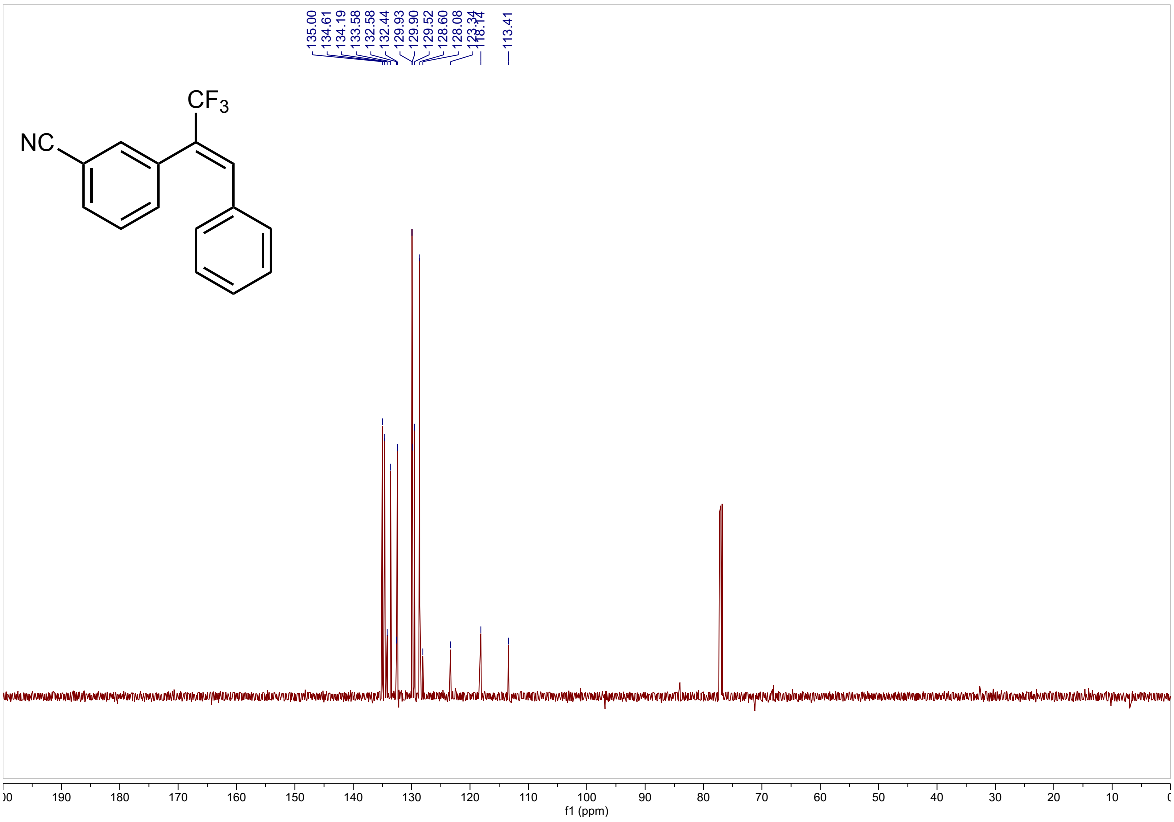

(*E*)-1-Methyl-2-(3,3,3-trifluoro-1-phenylprop-1-en-2-yl)benzene (11f)

^{1}H NMR (600 MHz, Chloroform-*d*)

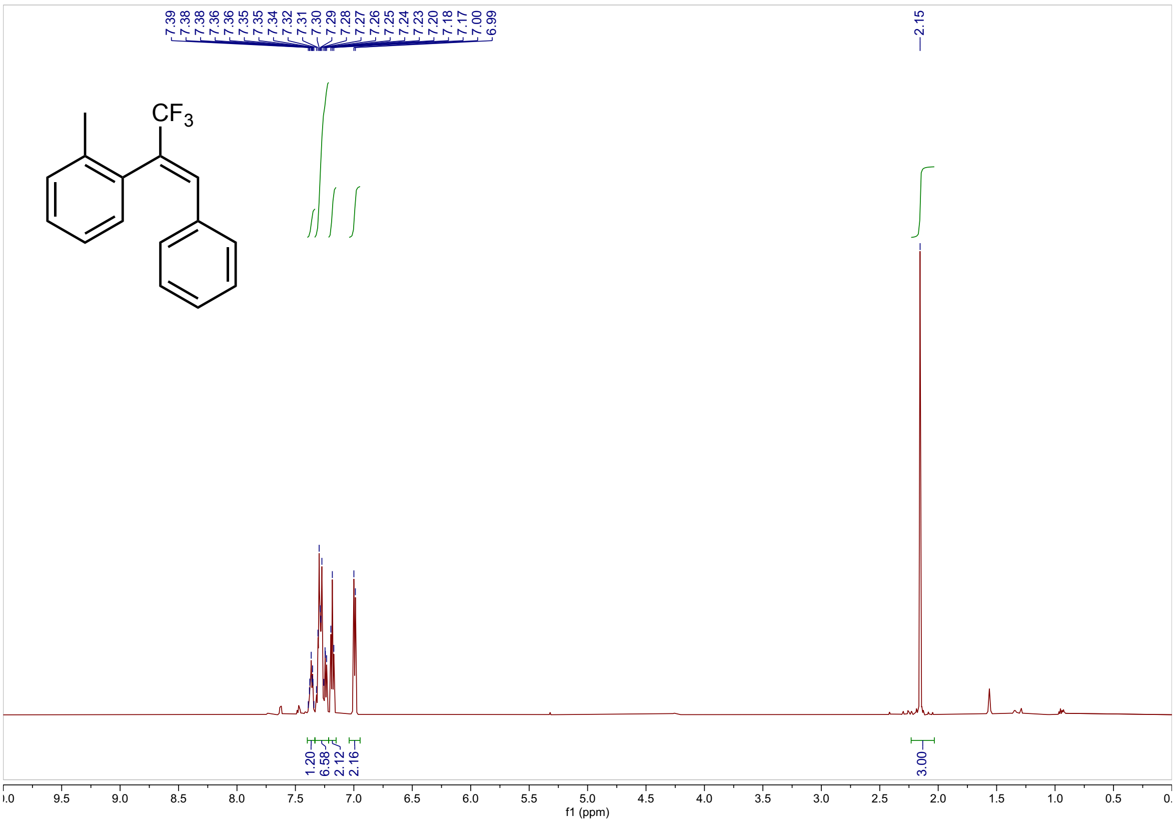

^{19}F NMR (565 MHz, Chloroform-*d*)

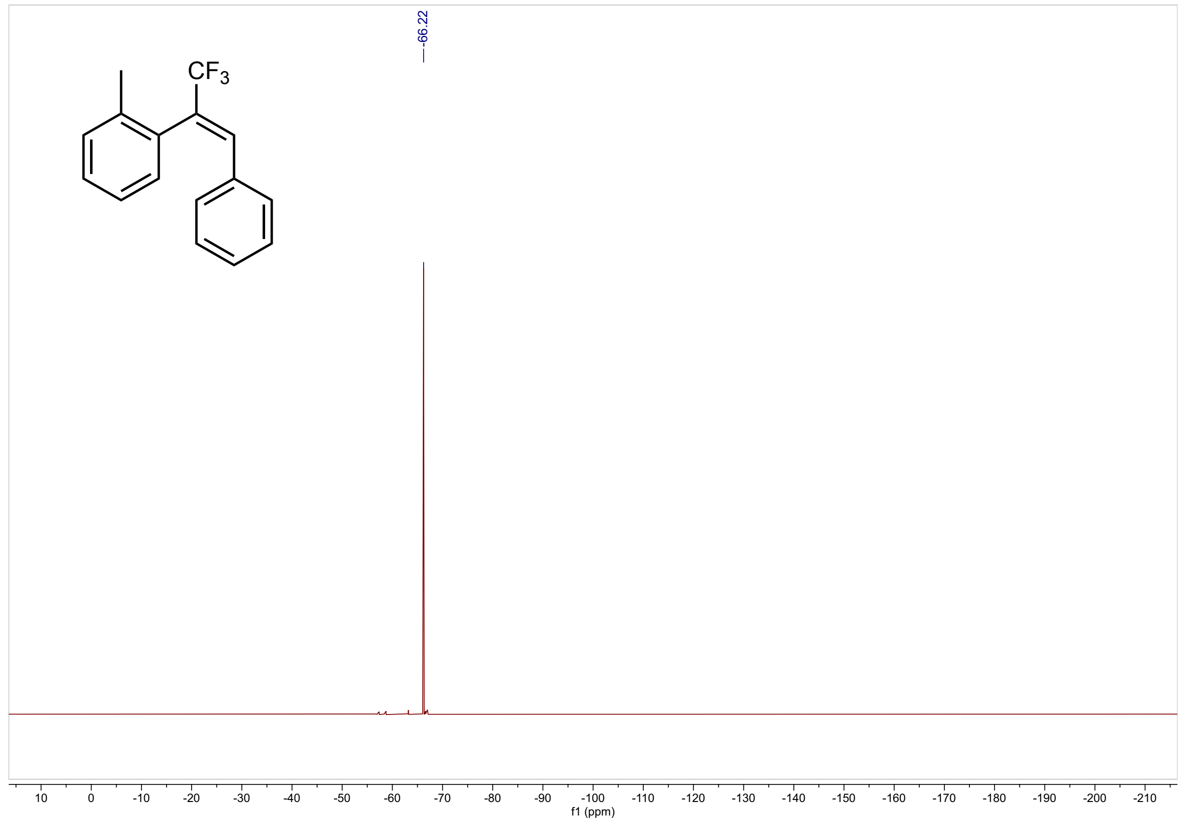

^{13}C NMR{^{1}H, ^{19}F} (151 MHz, Chloroform-*d*)

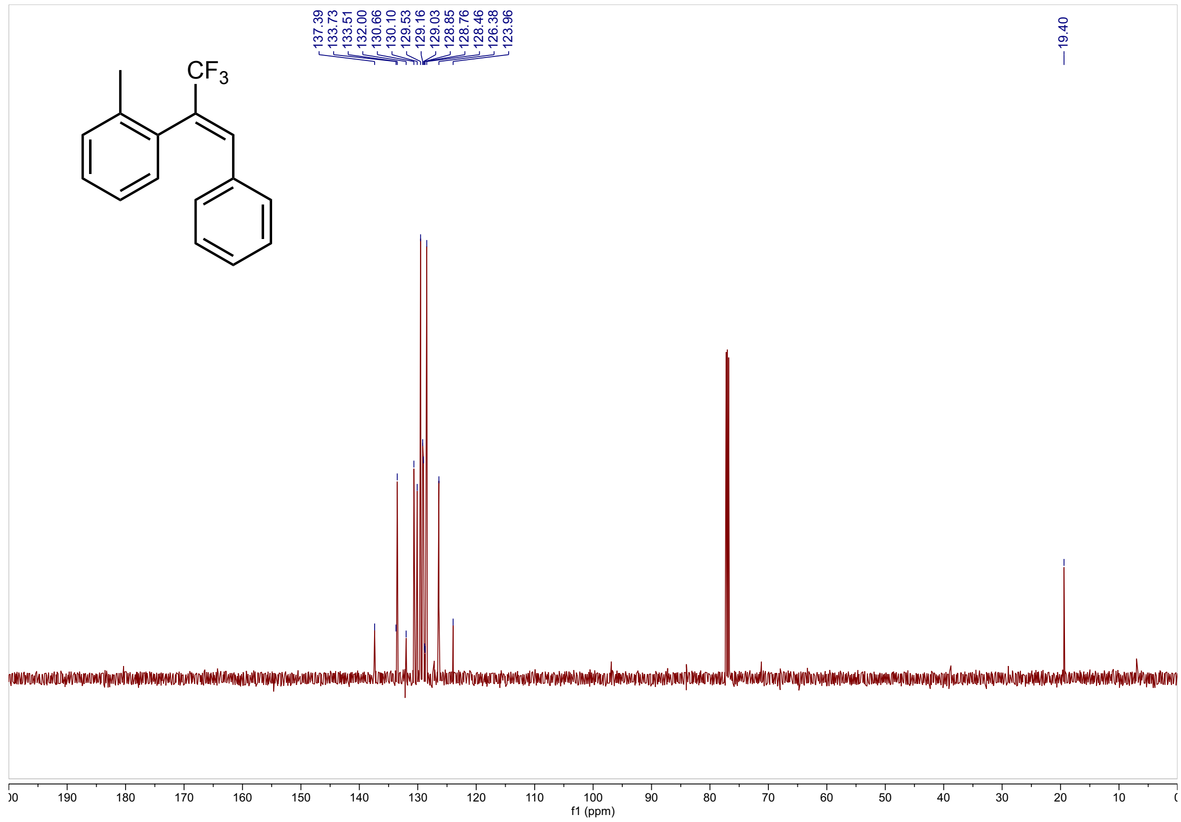

(*E*)-2-(3,3,3-Trifluoro-1-phenylprop-1-en-2-yl)naphthalene (11g)

^{1}H NMR (600 MHz, Chloroform-*d*)

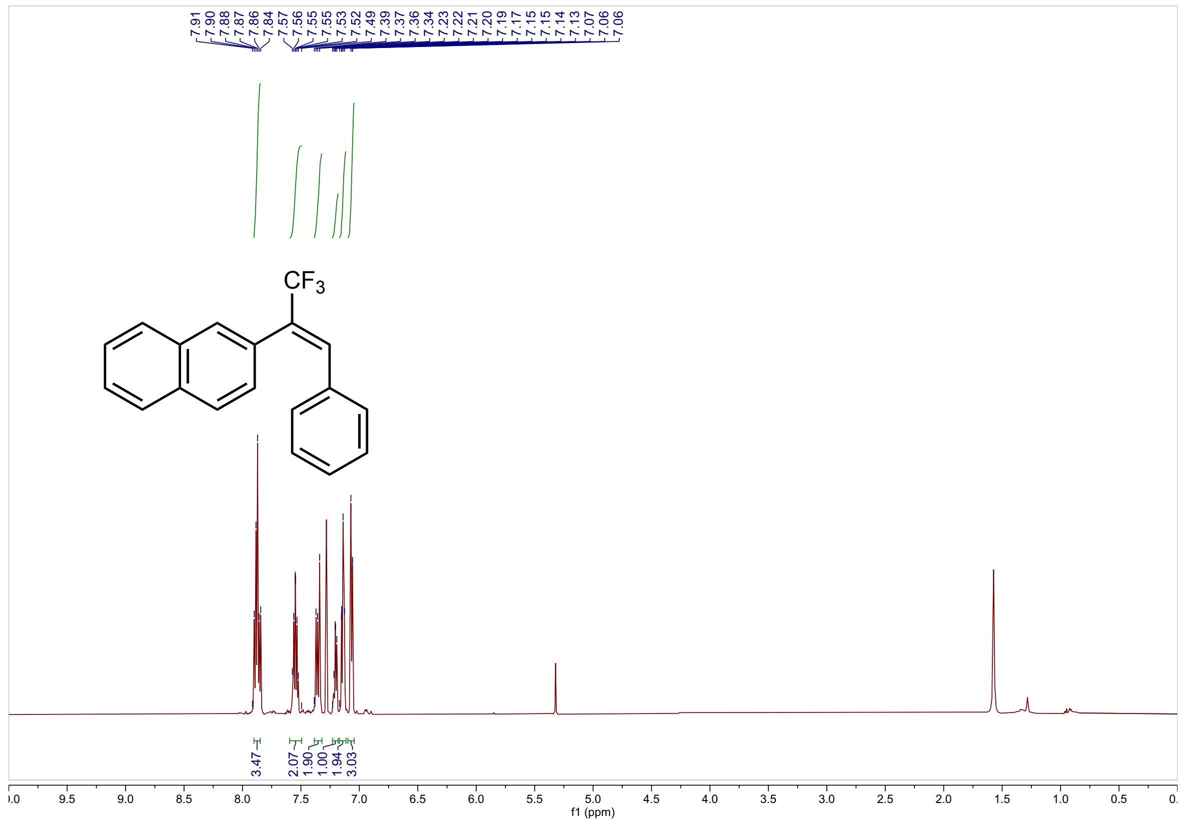

^{19}F NMR (565 MHz, Chloroform-*d*)

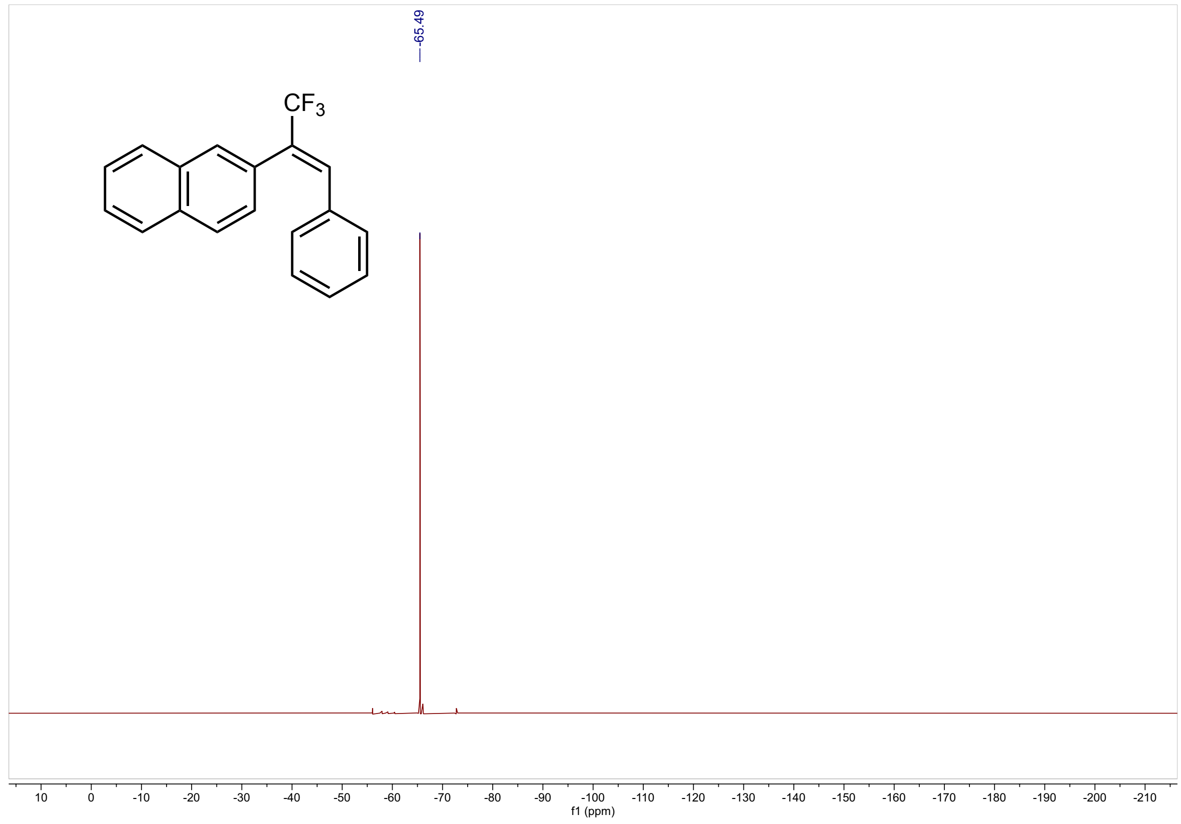

^{13}C NMR{^{1}H, ^{19}F} (151 MHz, Chloroform-*d*)

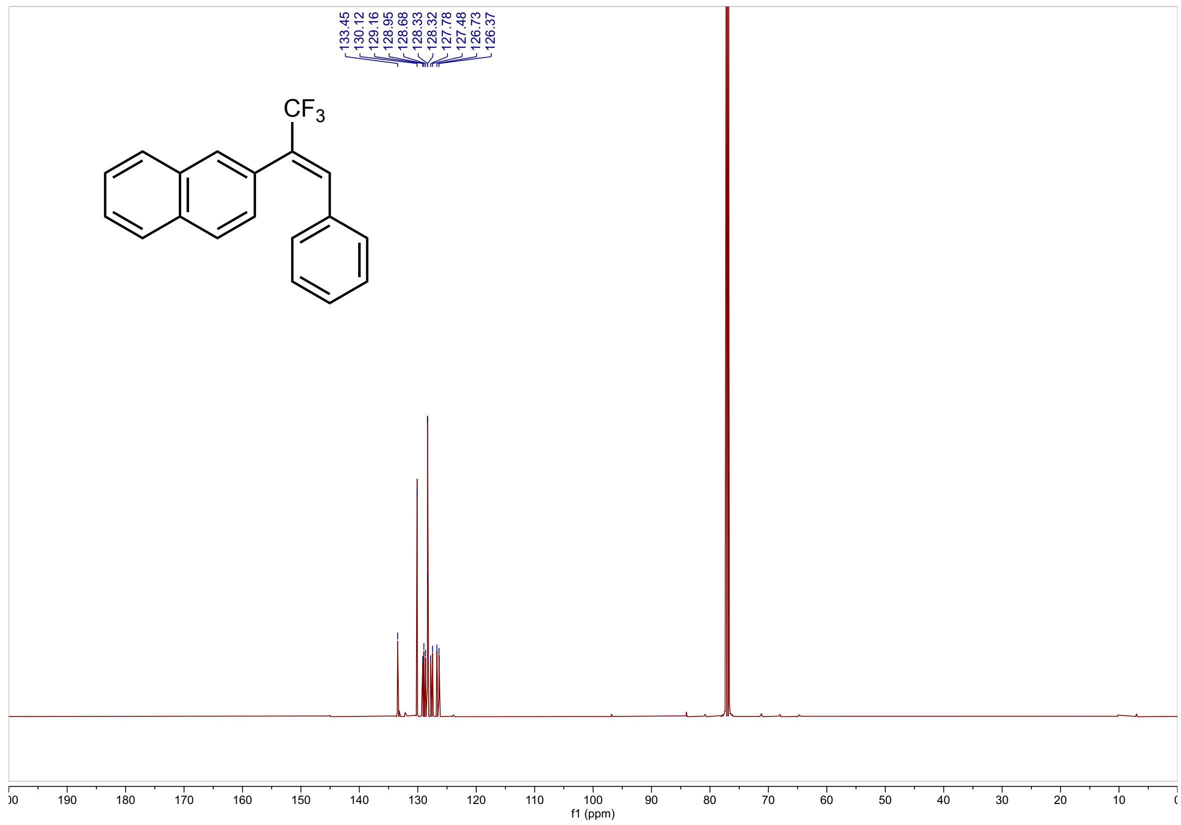

(*E*)-5-(3,3,3-Trifluoro-1-phenylprop-1-en-2-yl)benzo[*d*][1,3]dioxole (11h)

^{1}H NMR (600 MHz, Chloroform-*d*)

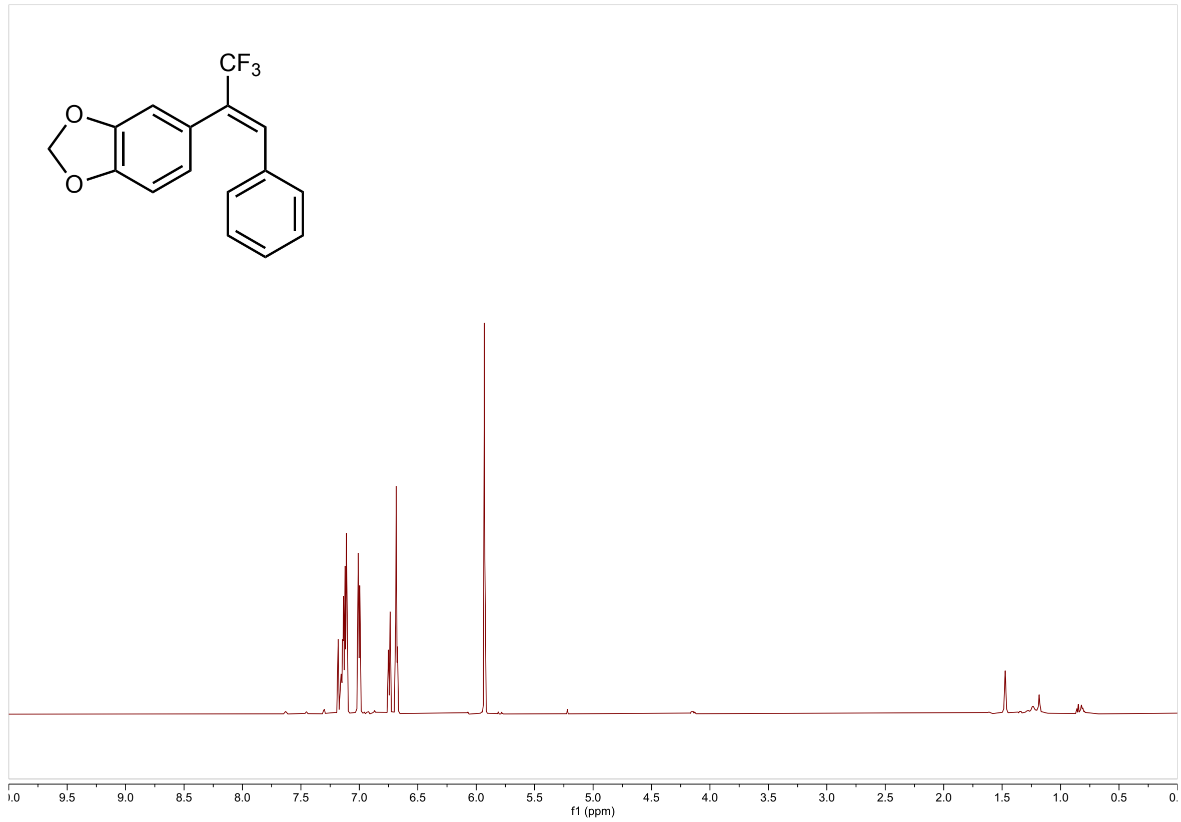

^{19}F NMR (565 MHz, Chloroform-*d*)

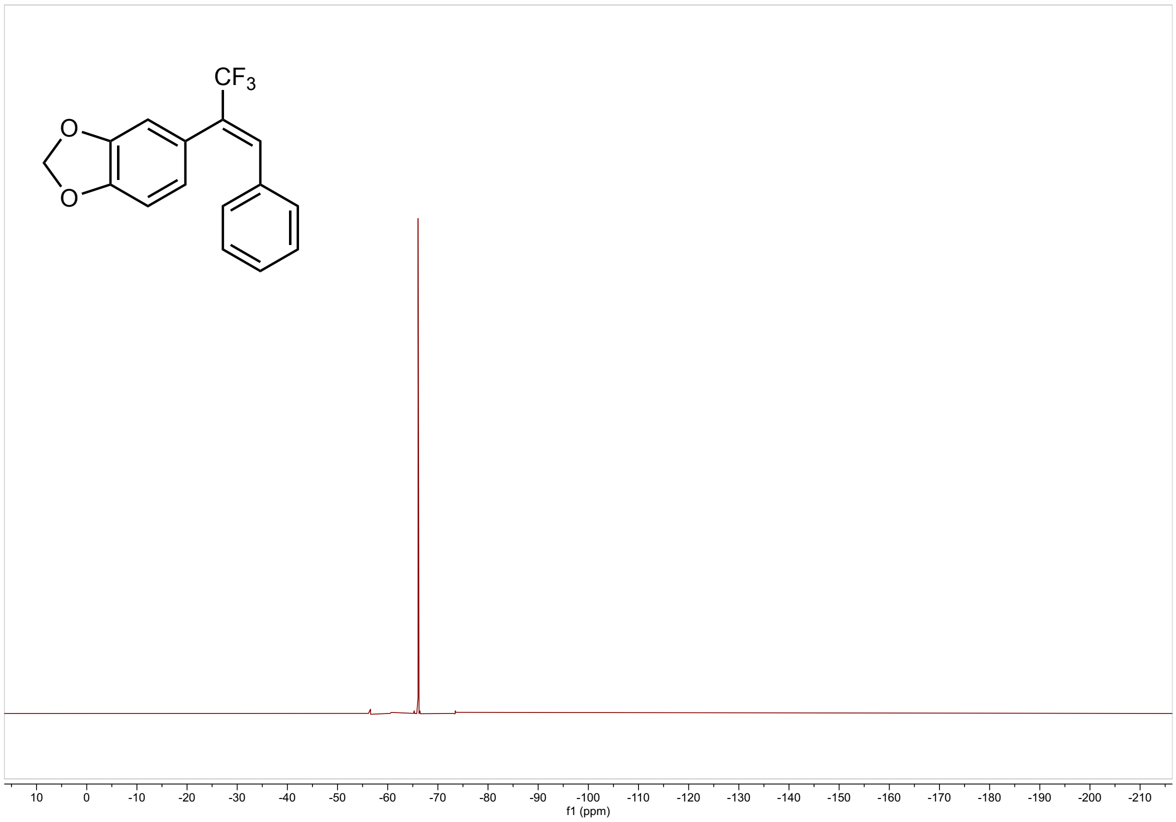

^{13}C NMR{^{1}H, ^{19}F} (151 MHz, Chloroform-*d*)

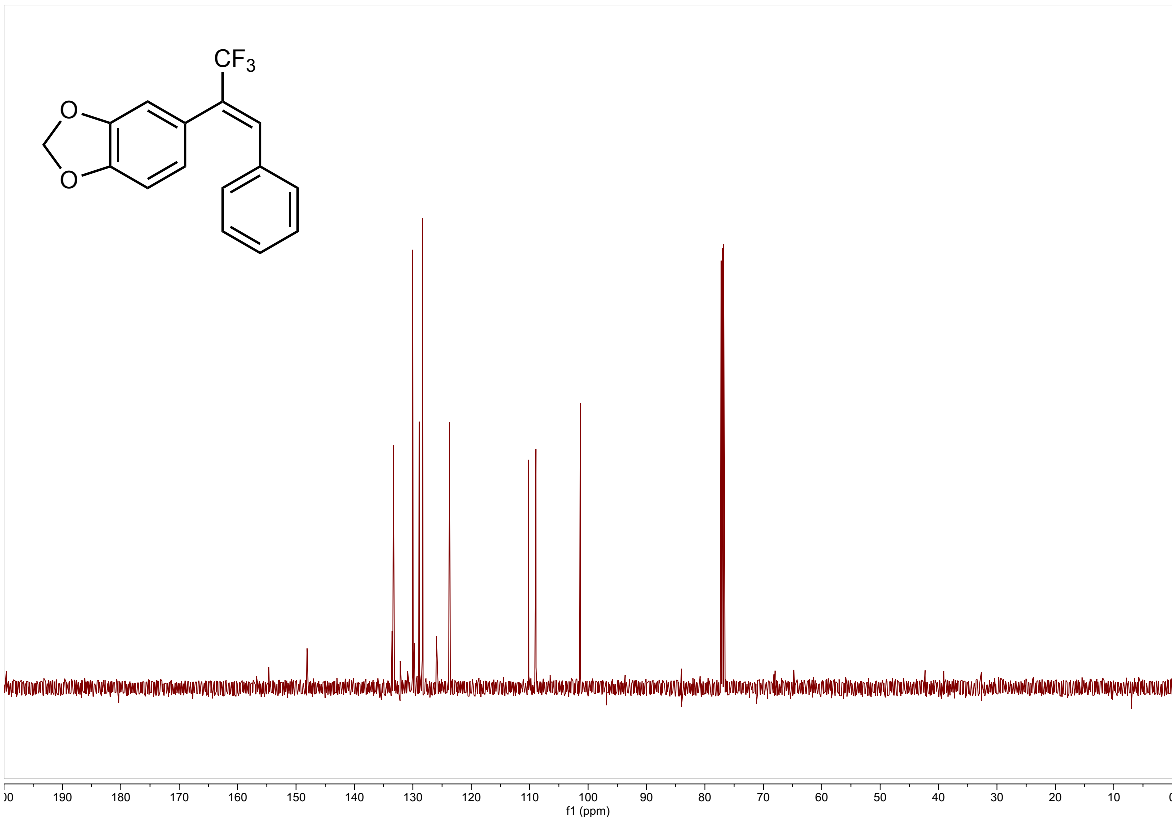

(*E*)-1-Methyl-4-(3,3,3-trifluoro-2-(4-nitrophenyl)prop-1-en-1-yl)benzene (12a)

^{1}H NMR (600 MHz, Chloroform-*d*)

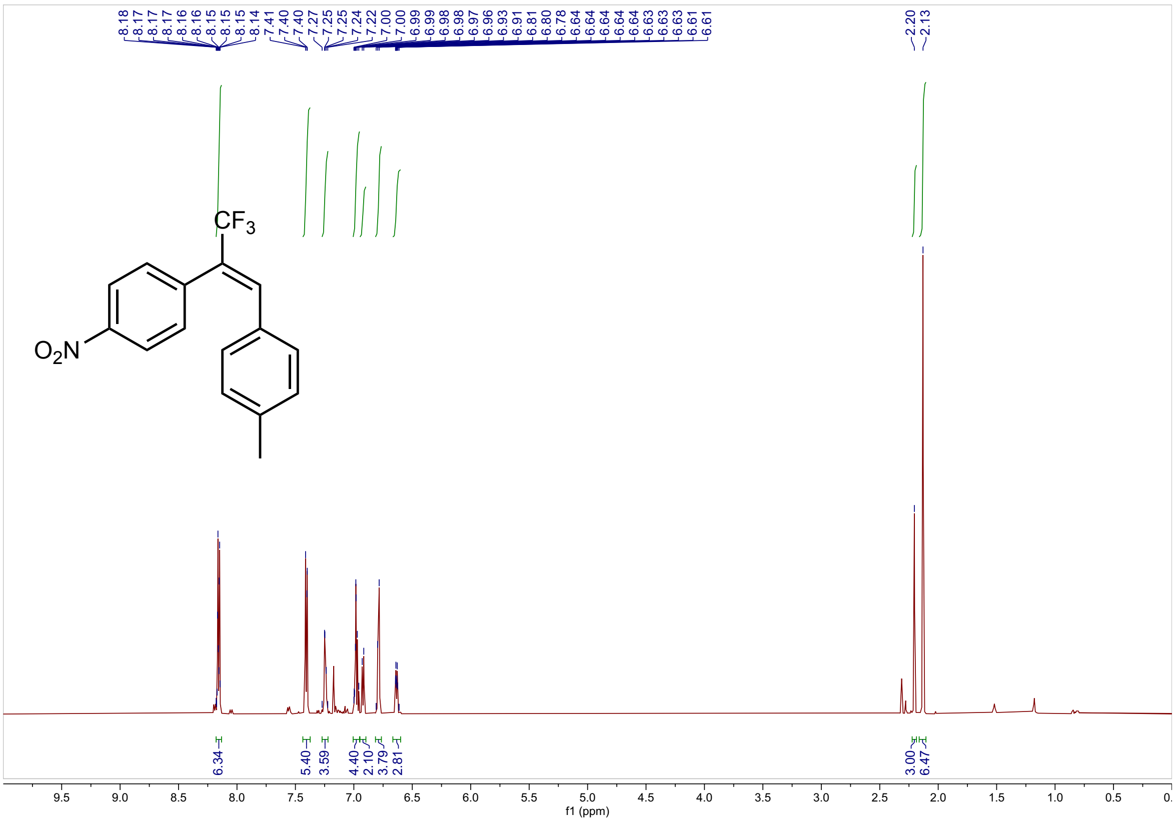

^{19}F NMR (565 MHz, Chloroform-*d*)

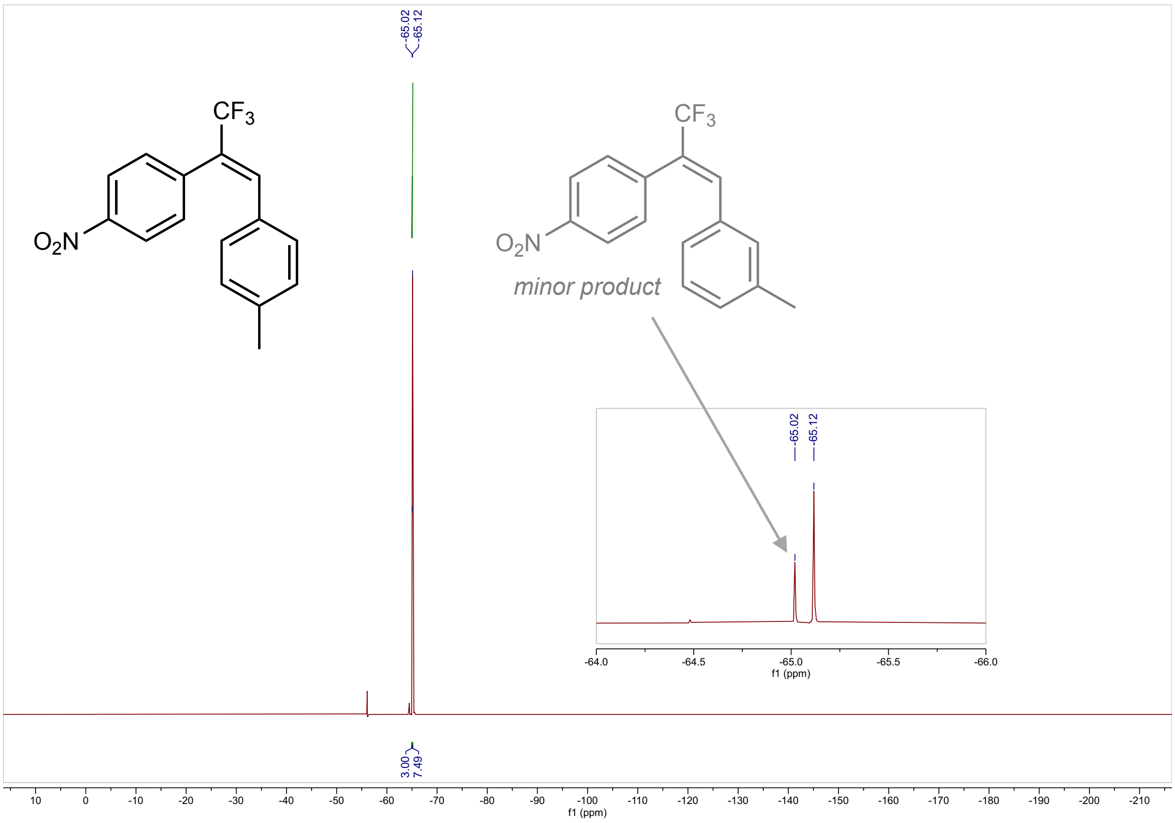

^{13}C NMR{^{1}H, ^{19}F} (151 MHz, Chloroform-*d*)

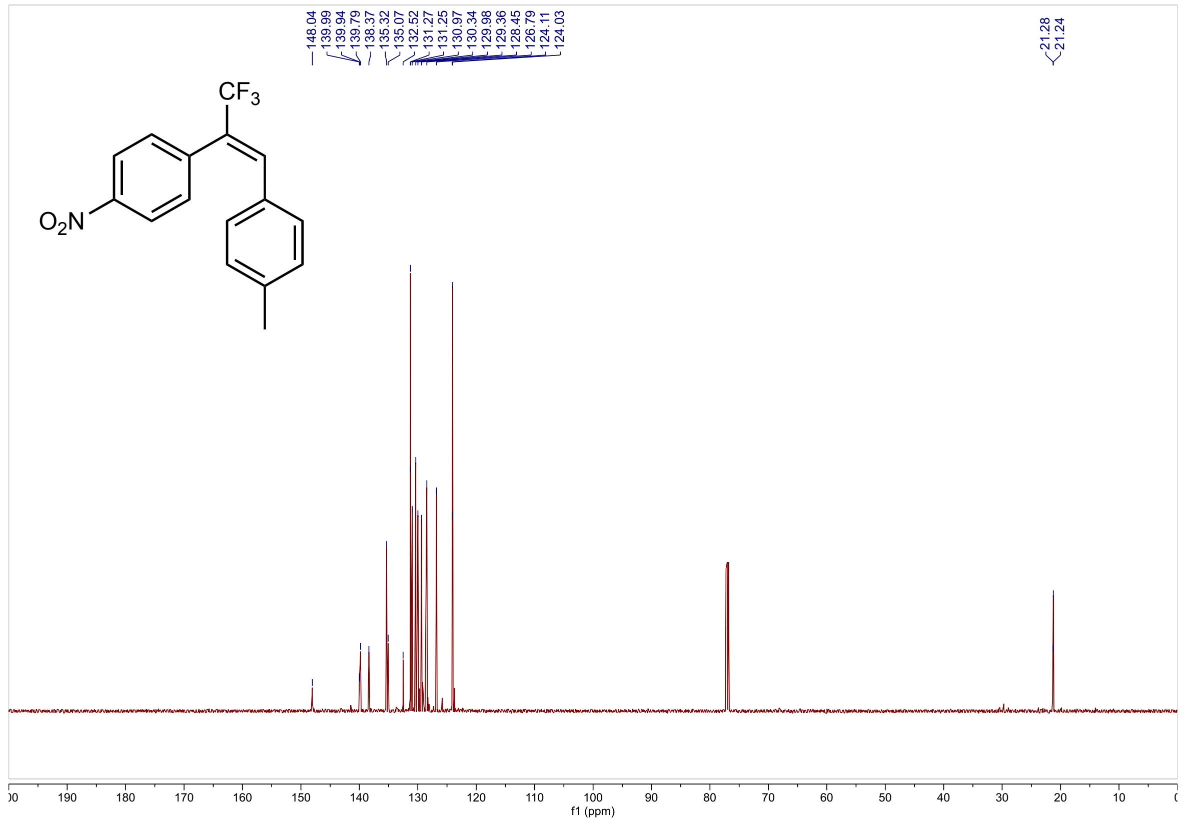

(*E*)-1,2-Dimethyl-4-(3,3,3-trifluoro-2-(4-nitrophenyl)prop-1-en-1-yl)benzene (12b)

^{1}H NMR (600 MHz, Chloroform-*d*)

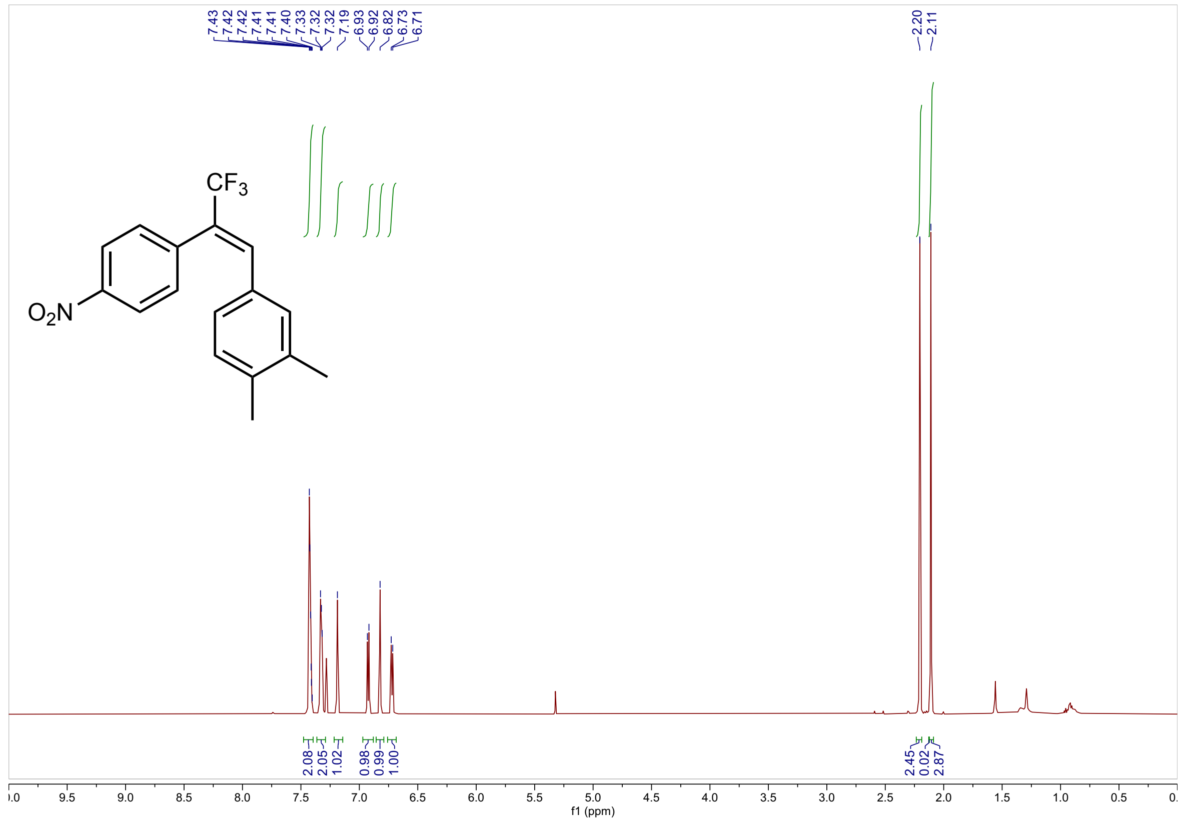

^{19}F NMR (565 MHz, Chloroform-*d*)

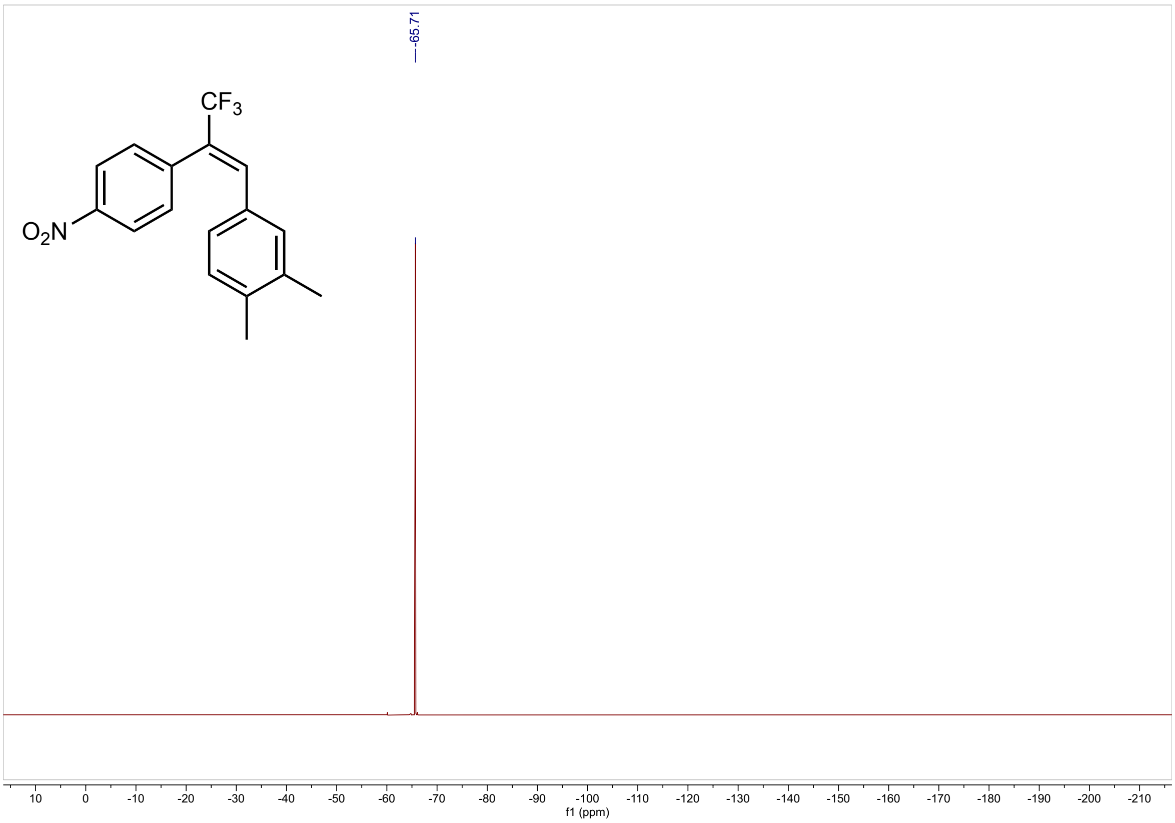

^{13}C NMR{^{1}H, ^{19}F} (151 MHz, Chloroform-*d*)

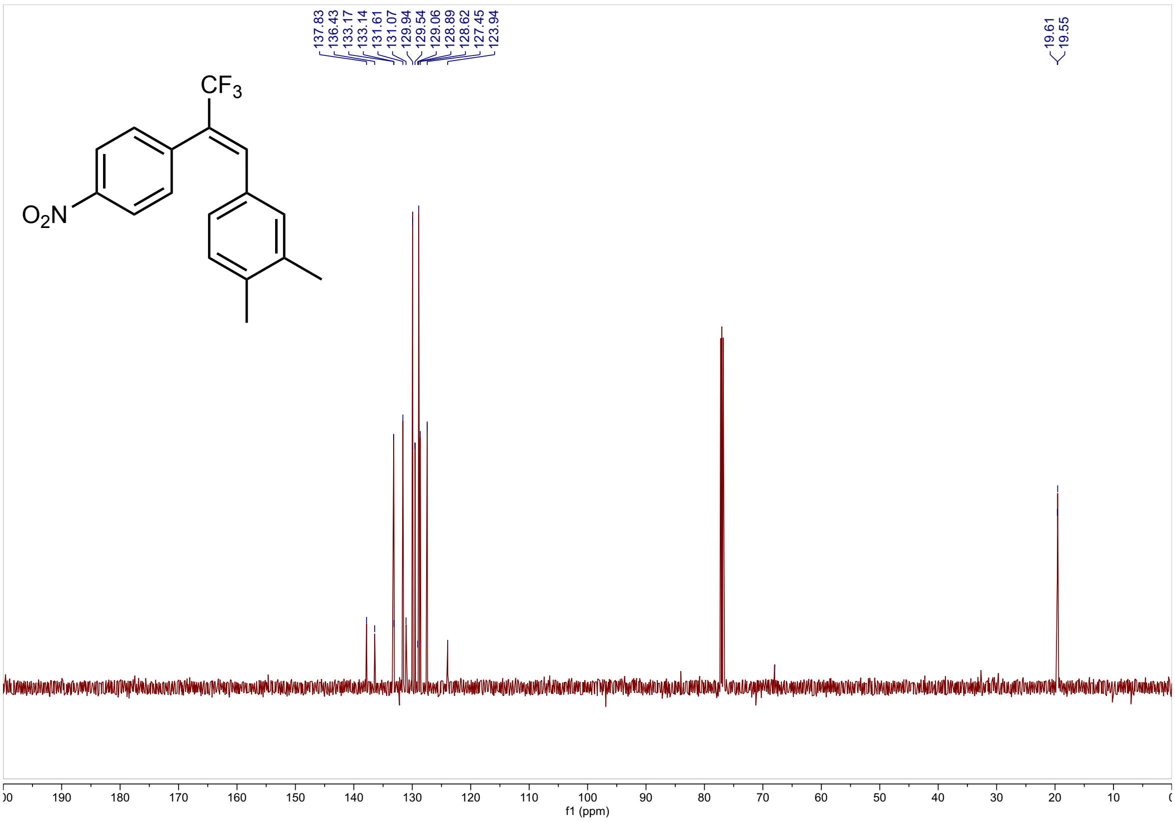

(*E*)-1,3-Dimethyl-5-(3,3,3-trifluoro-2-(4-nitrophenyl)prop-1-en-1-yl)benzene (12c)

^{1}H NMR (600 MHz, Chloroform-*d*)

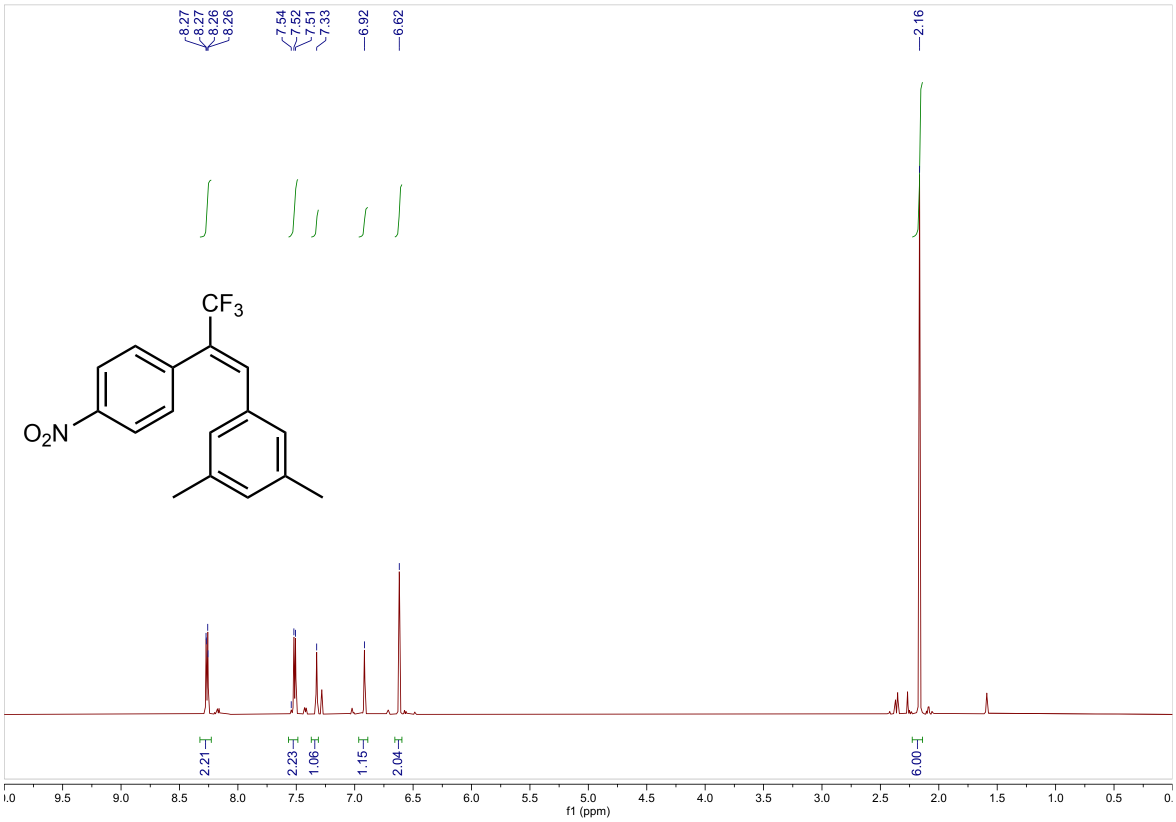

^{19}F NMR (565 MHz, Chloroform-*d*)

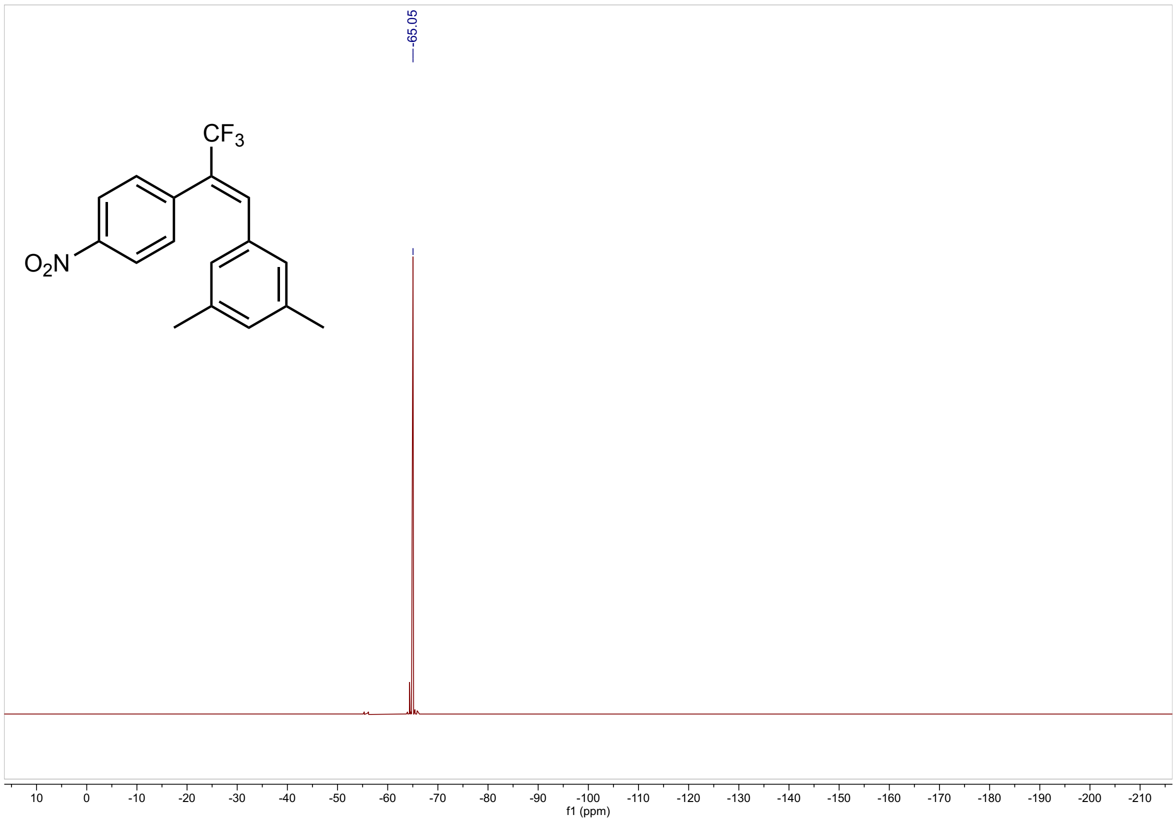

^{13}C NMR{^{1}H, ^{19}F} (151 MHz, Chloroform-*d*)

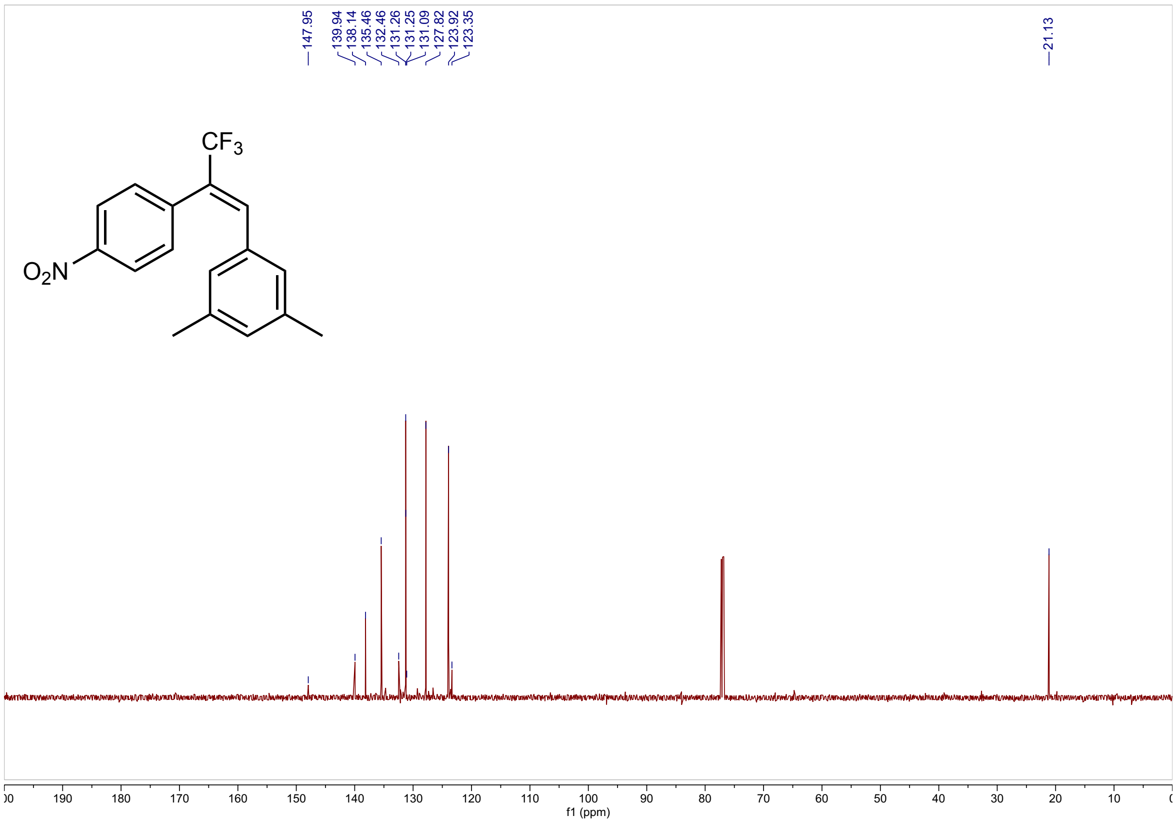

(*E*)-1,4-Dimethyl-2-(3,3,3-trifluoro-2-(4-nitrophenyl)prop-1-en-1-yl)benzene (12d)

^{1}H NMR (600 MHz, Chloroform-*d*)

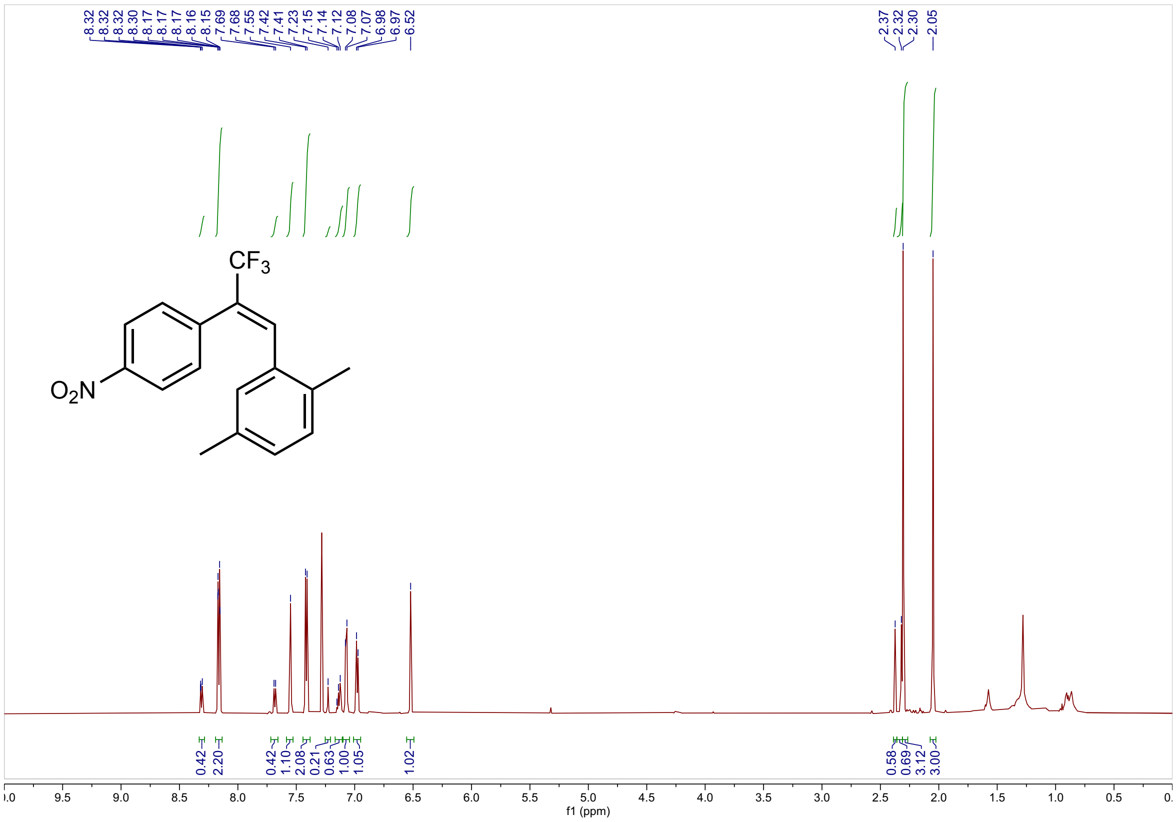

19F NMR (565 MHz, Chloroform-*d*)

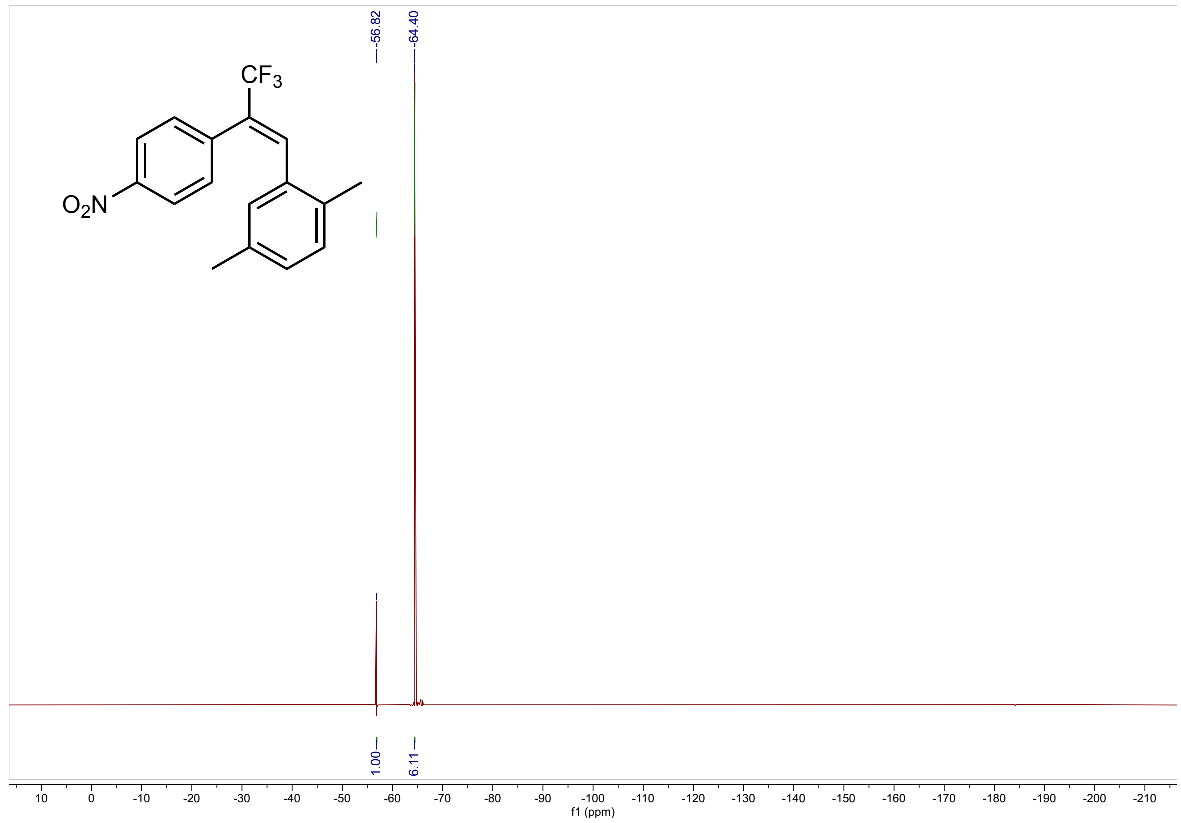

13C NMR{1H, 19F} (151 MHz, Chloroform-*d*):

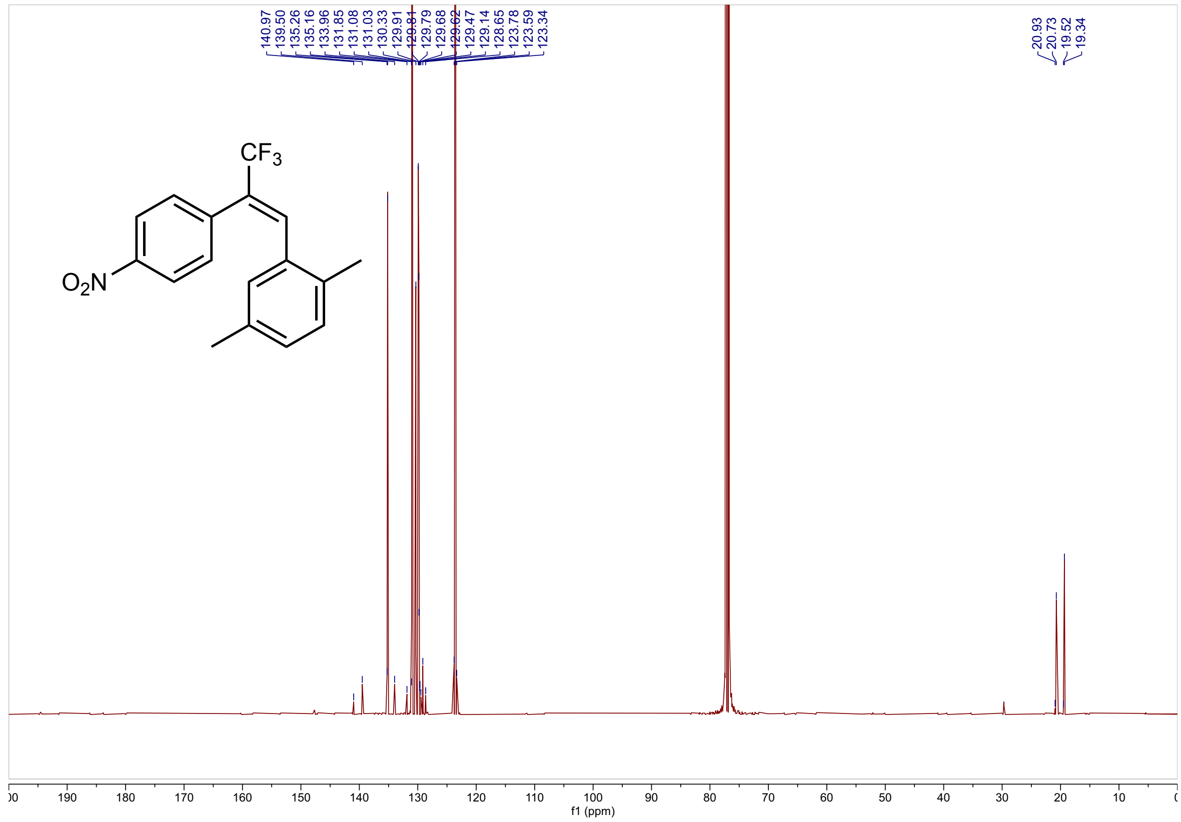

(*E*)-1-Isobutyl-4-(3,3,3-trifluoro-2-(4-nitrophenyl)prop-1-en-1-yl)benzene (12e)

^{1}H NMR (600 MHz, Chloroform-*d*)

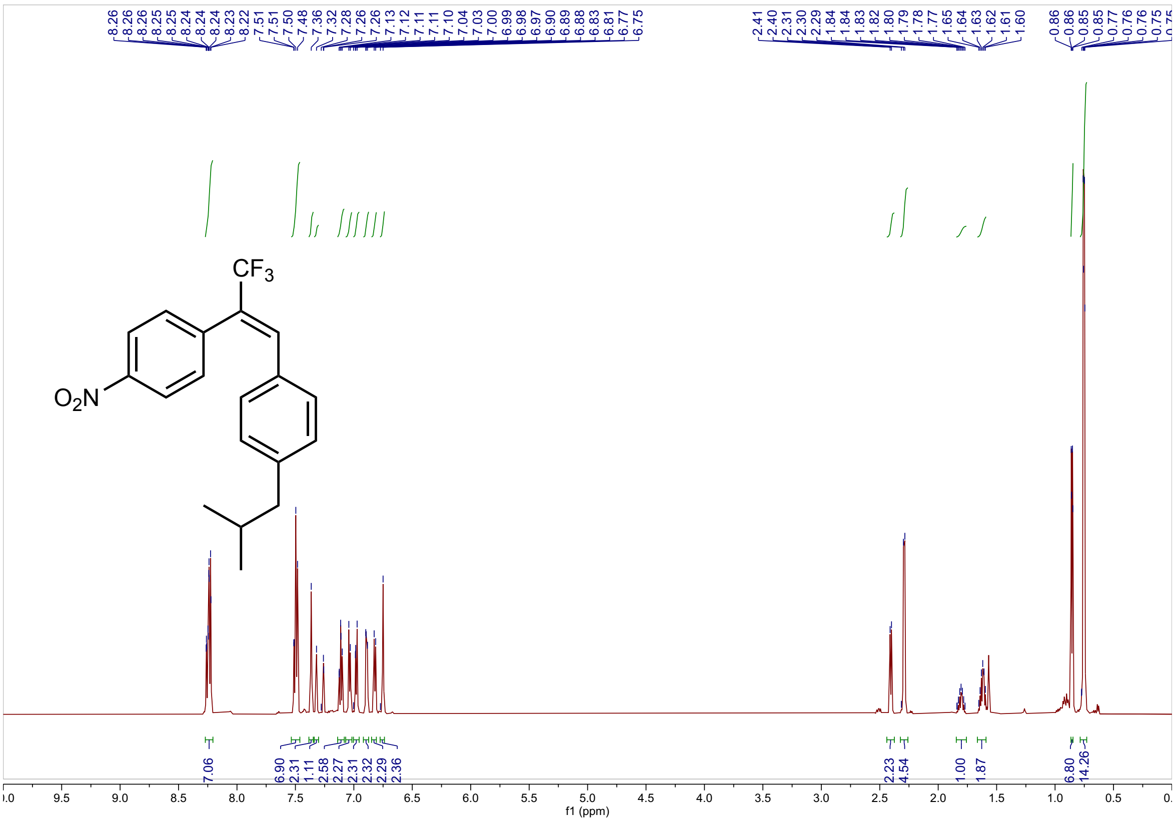

^{19}F NMR (565 MHz, Chloroform-*d*)

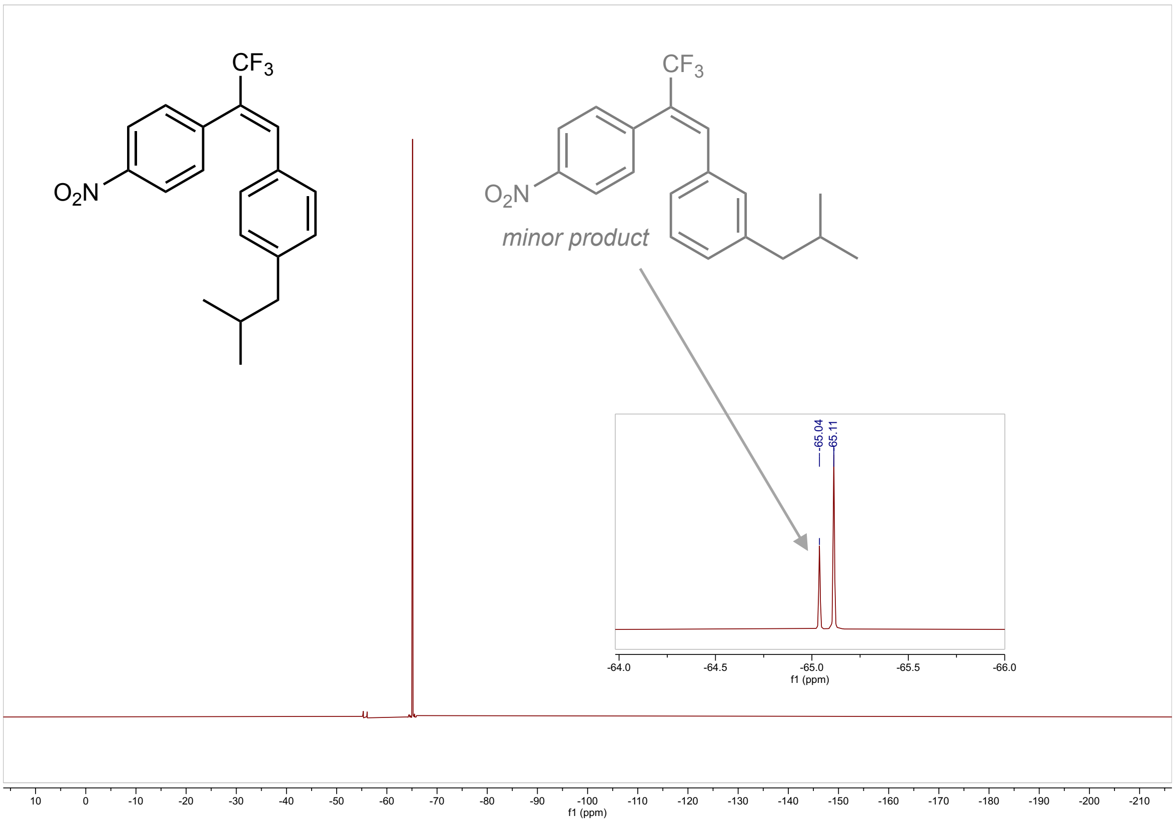

^{13}C NMR{^{1}H, ^{19}F} (151 MHz, Chloroform-*d*)

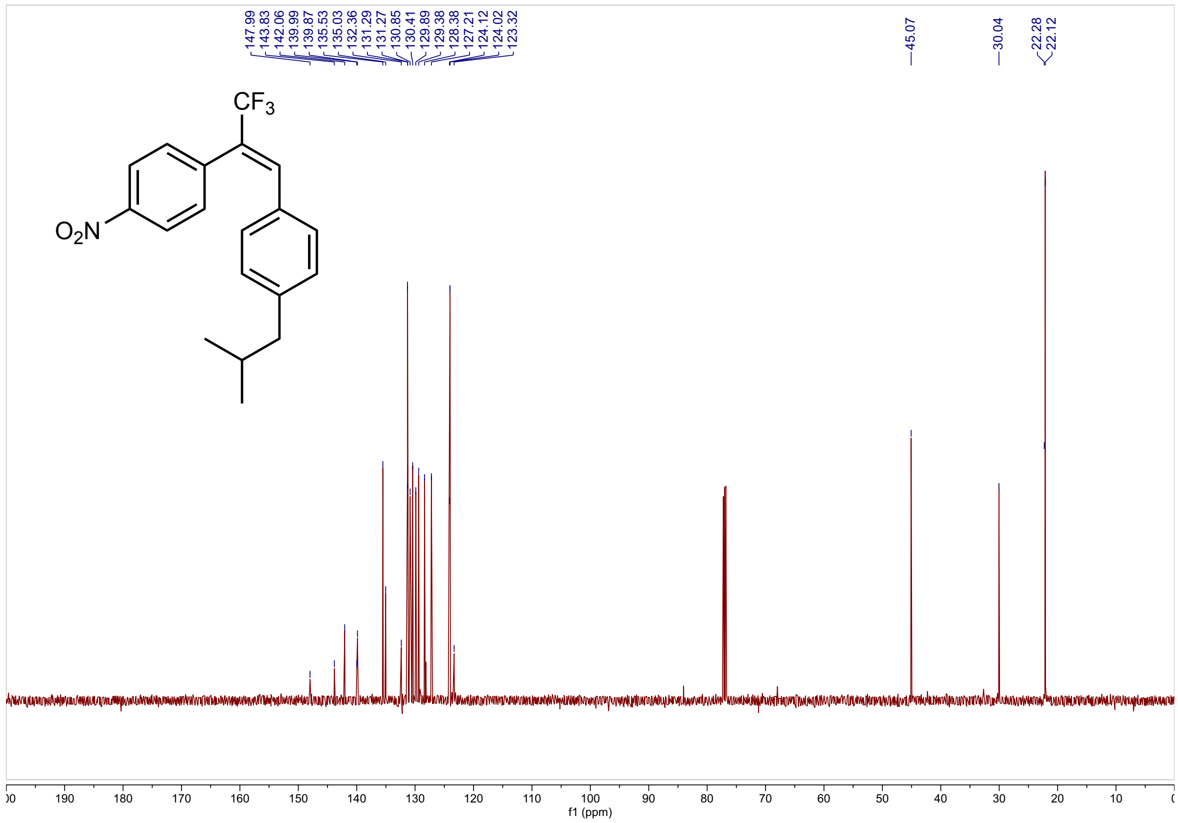

(*E*)-1-(*tert*-Butyl)-4-(3,3,3-trifluoro-2-(4-nitrophenyl)prop-1-en-1-yl)benzene (12f)

^{1}H NMR (600 MHz, Chloroform-*d*)

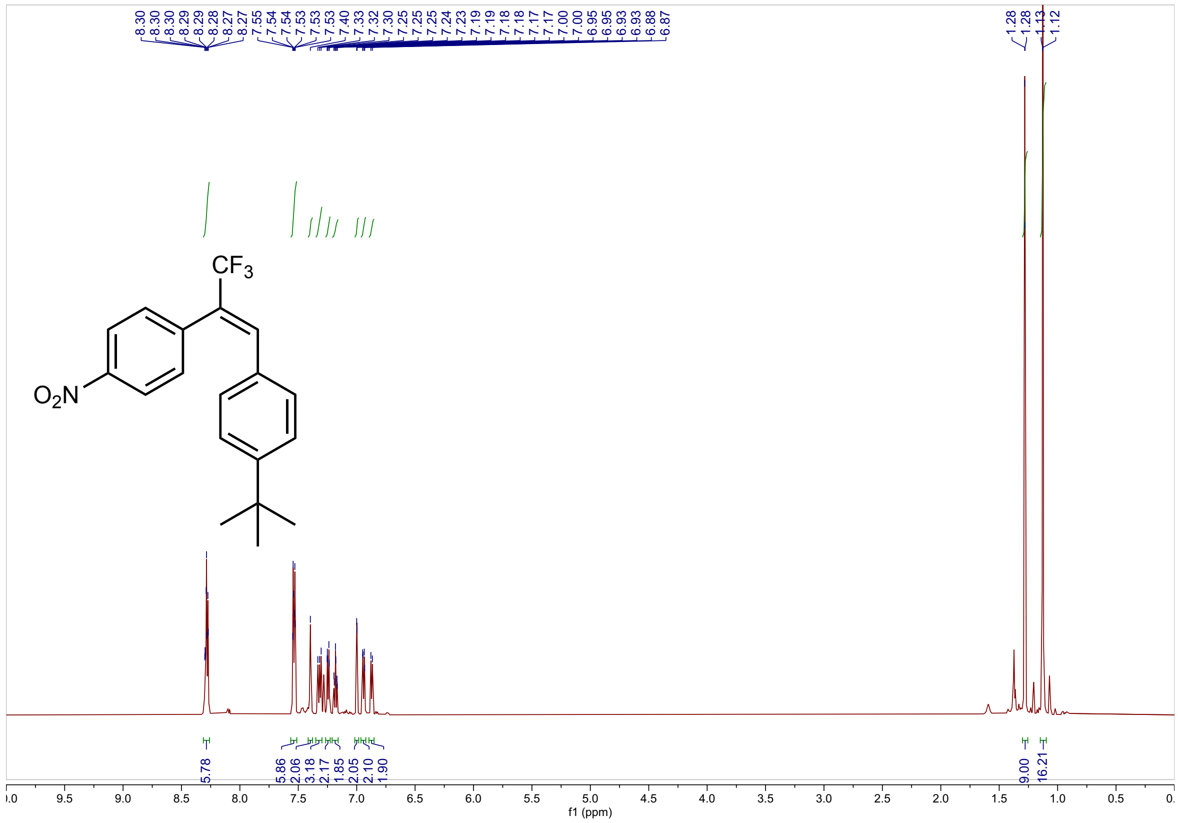

^{19}F NMR (565 MHz, Chloroform-*d*)

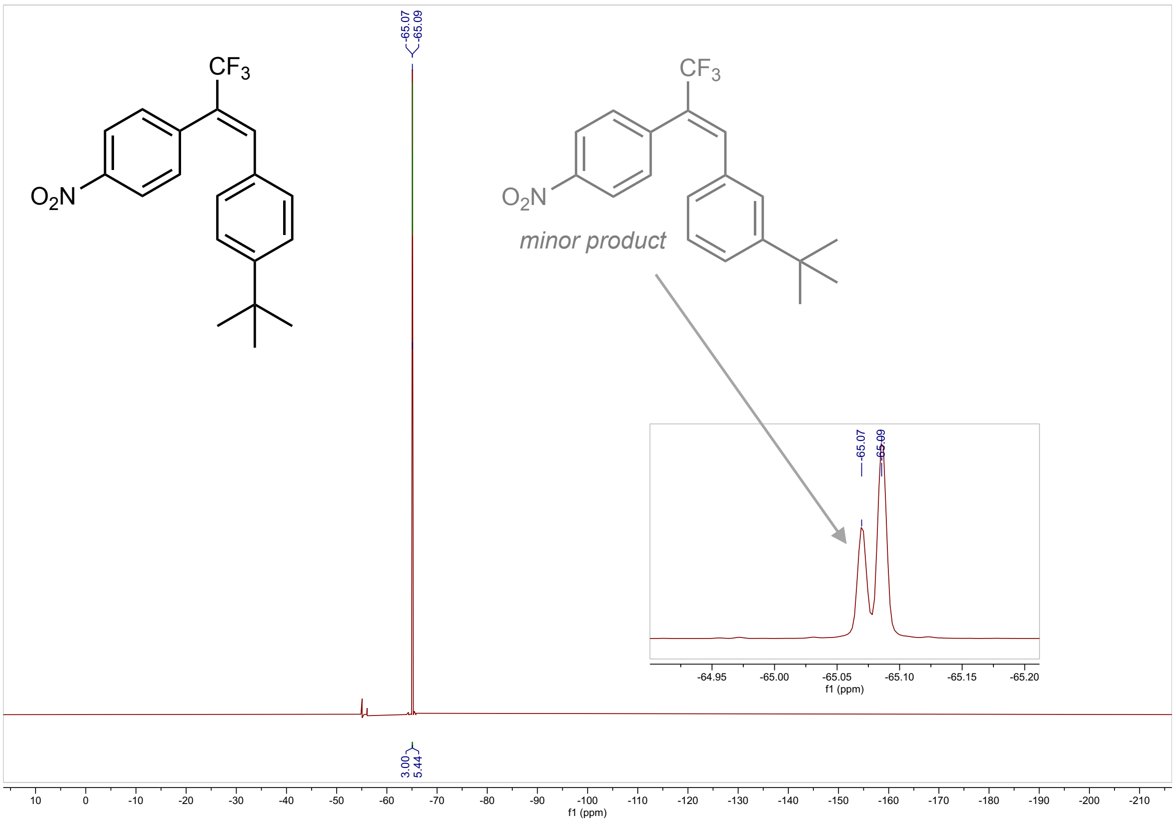

^{13}C NMR{^{1}H, ^{19}F} (151 MHz, Chloroform-*d*)

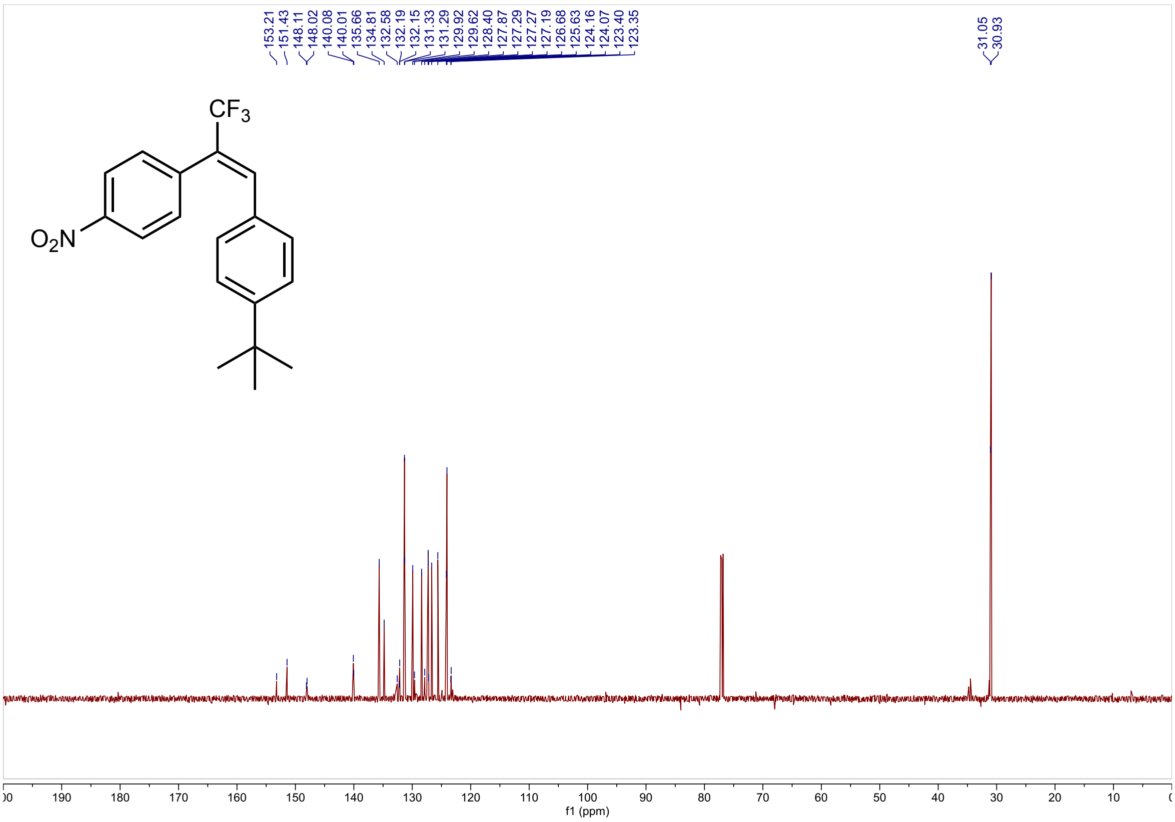

(*E*)-1-cyclohexyl-4-(3,3,3-trifluoro-2-(4-nitrophenyl)prop-1-en-1-yl)benzene (12g)

^{1}H NMR (600 MHz, Chloroform-*d*)

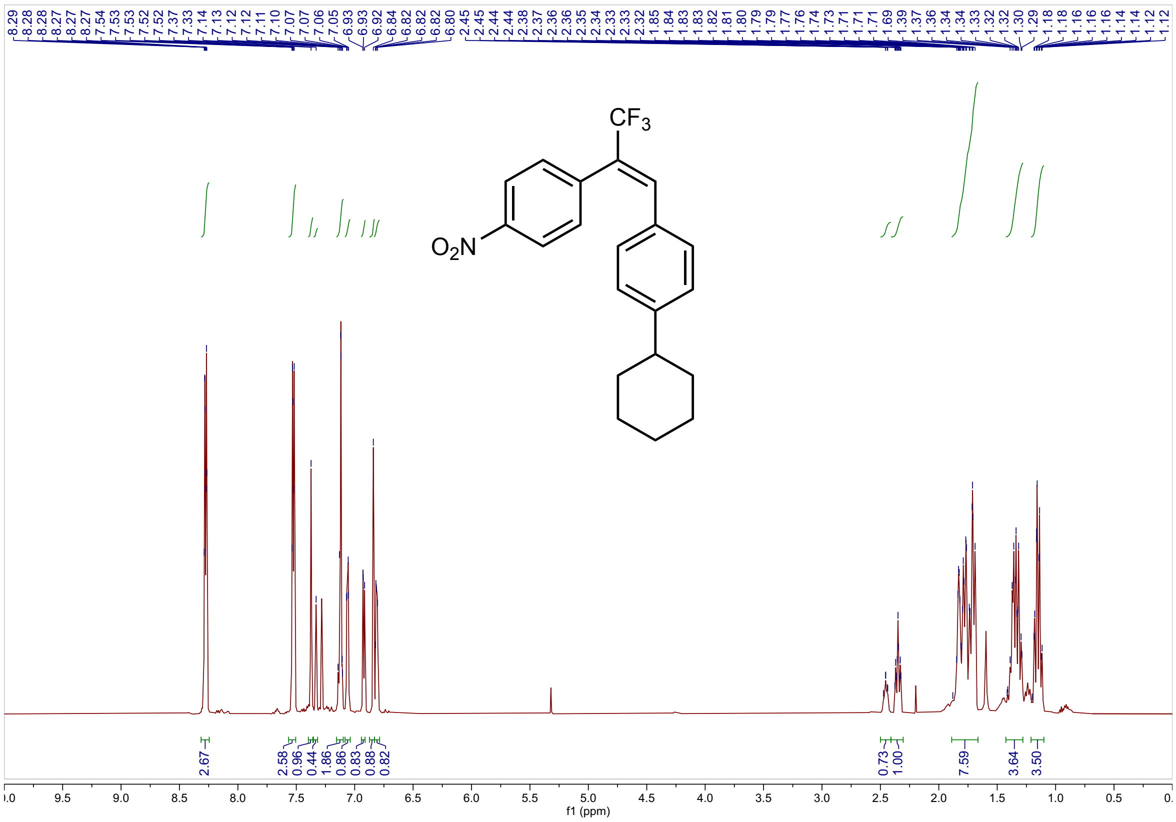

^{19}F NMR (565 MHz, Chloroform-*d*)

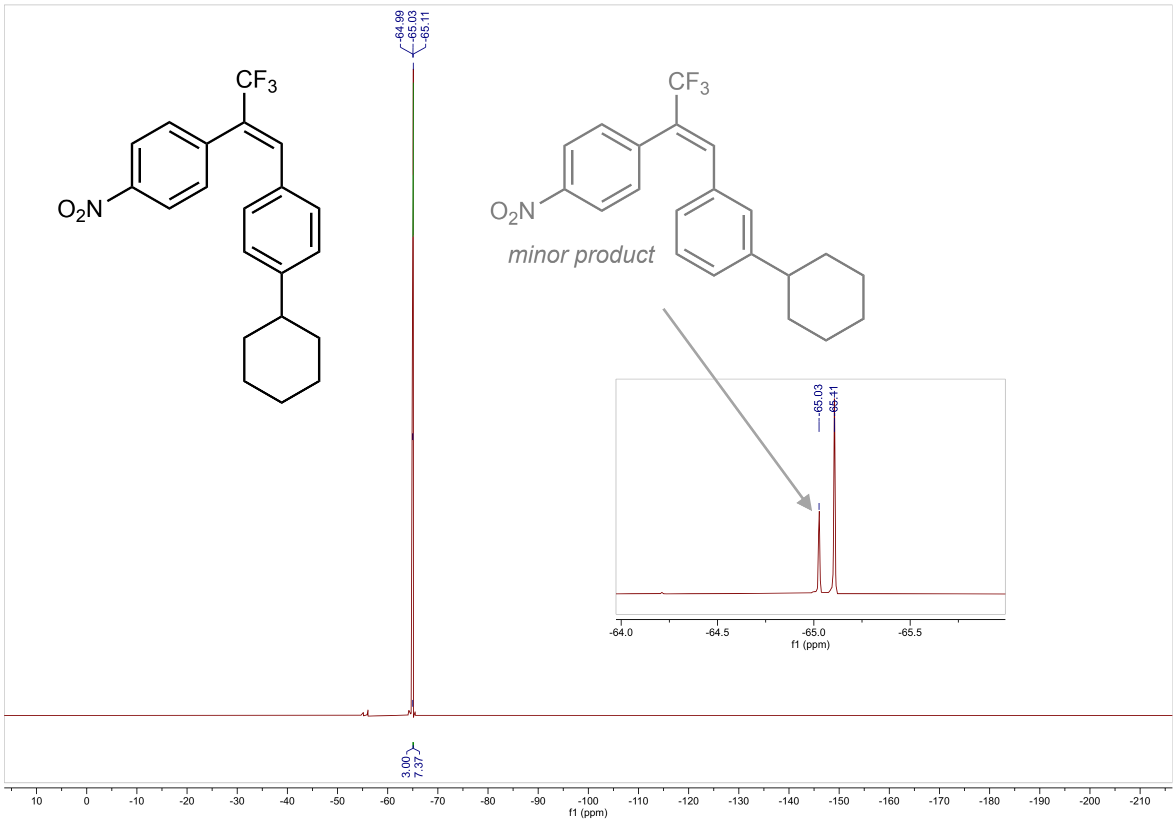

^{13}C NMR{^{1}H, ^{19}F} (151 MHz, Chloroform-*d*)

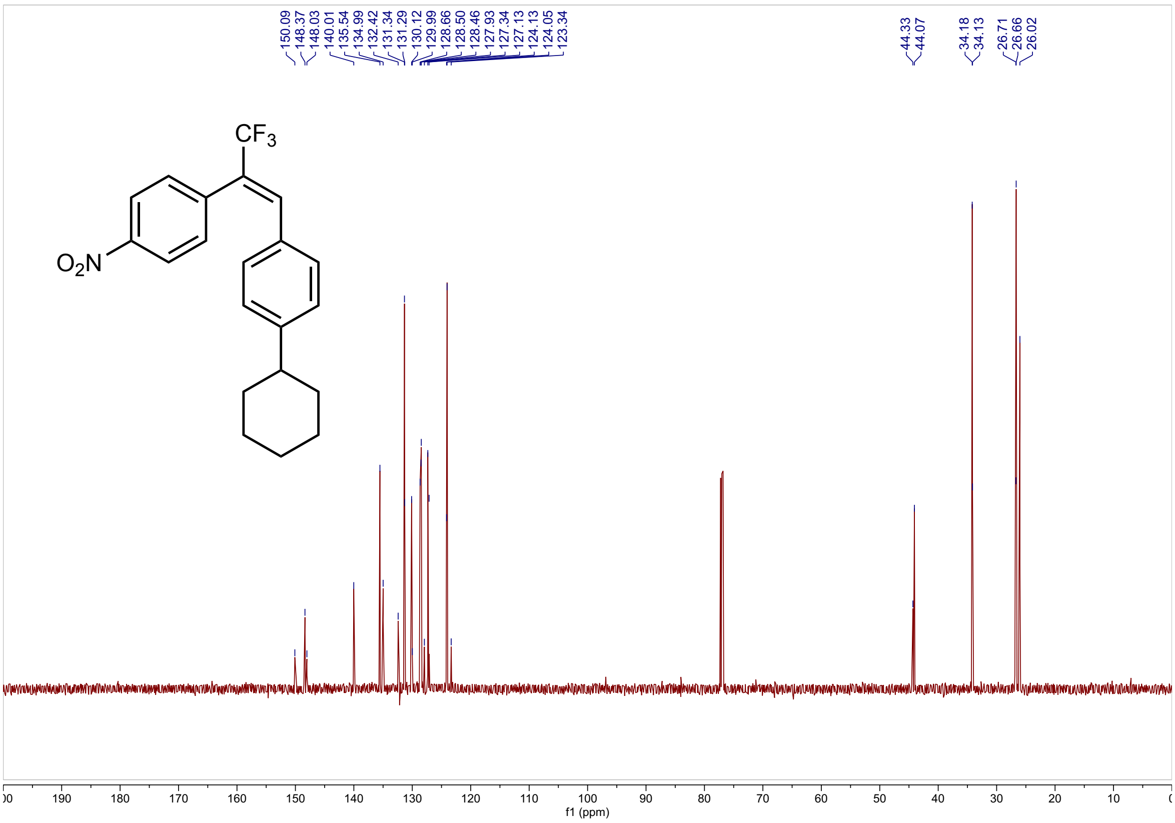

(*E*)-5-(3,3,3-Trifluoro-2-(4-nitrophenyl)prop-1-en-1-yl)-2,3-dihydro-1*H*-indene (12h)

^{1}H NMR (600 MHz, Chloroform-*d*)

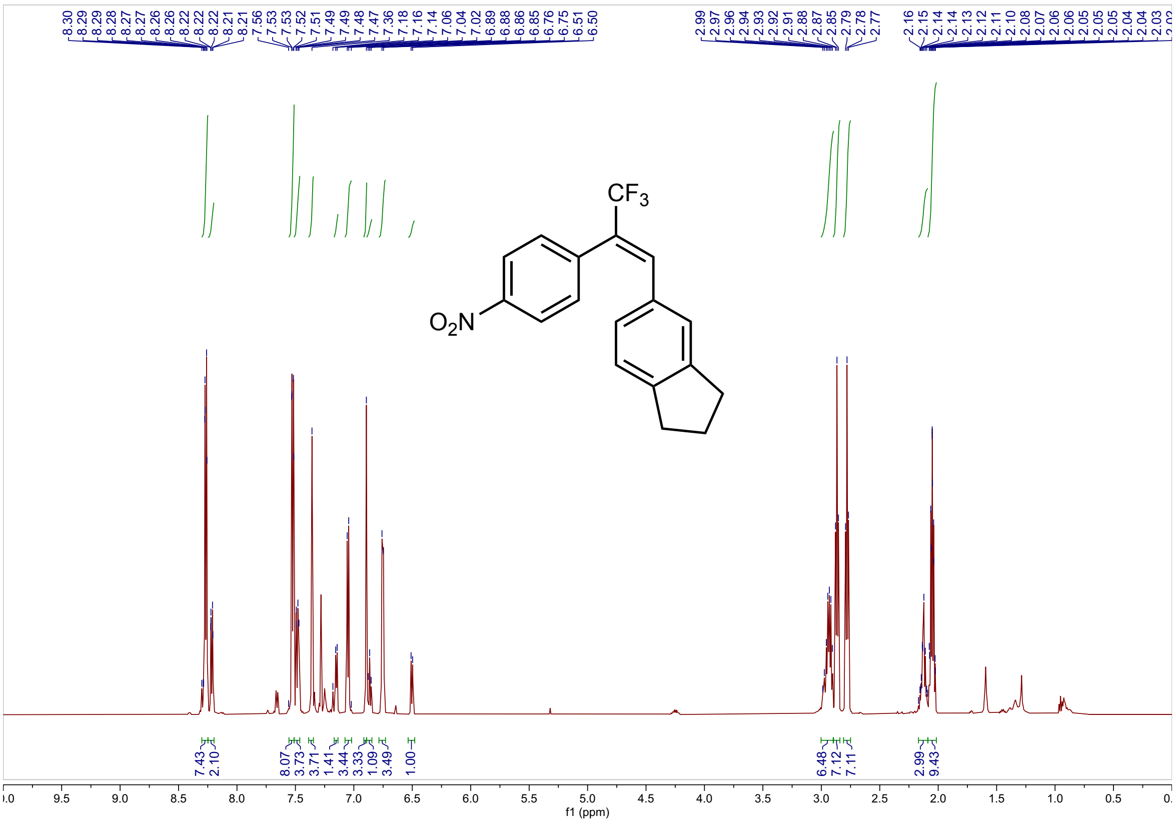

^{19}F NMR (565 MHz, Chloroform-*d*)

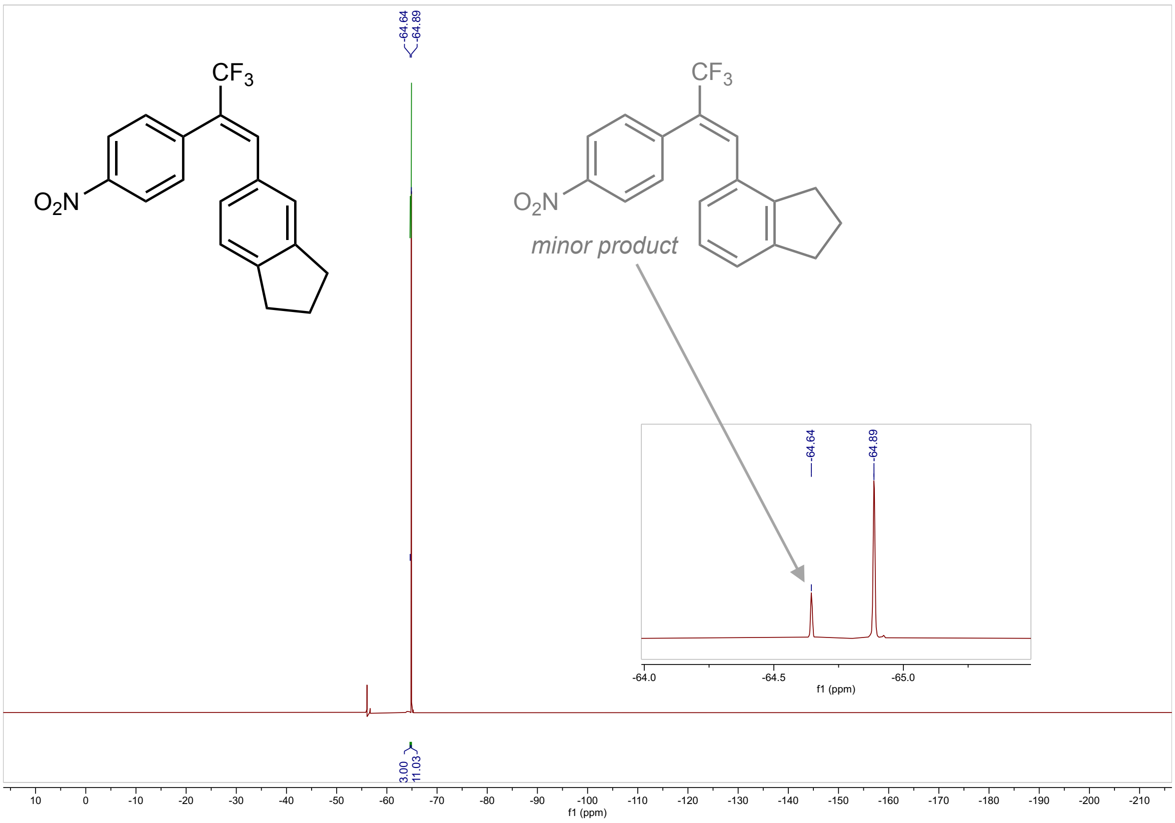

^{13}C NMR{^{1}H, ^{19}F} (151 MHz, Chloroform-*d*)

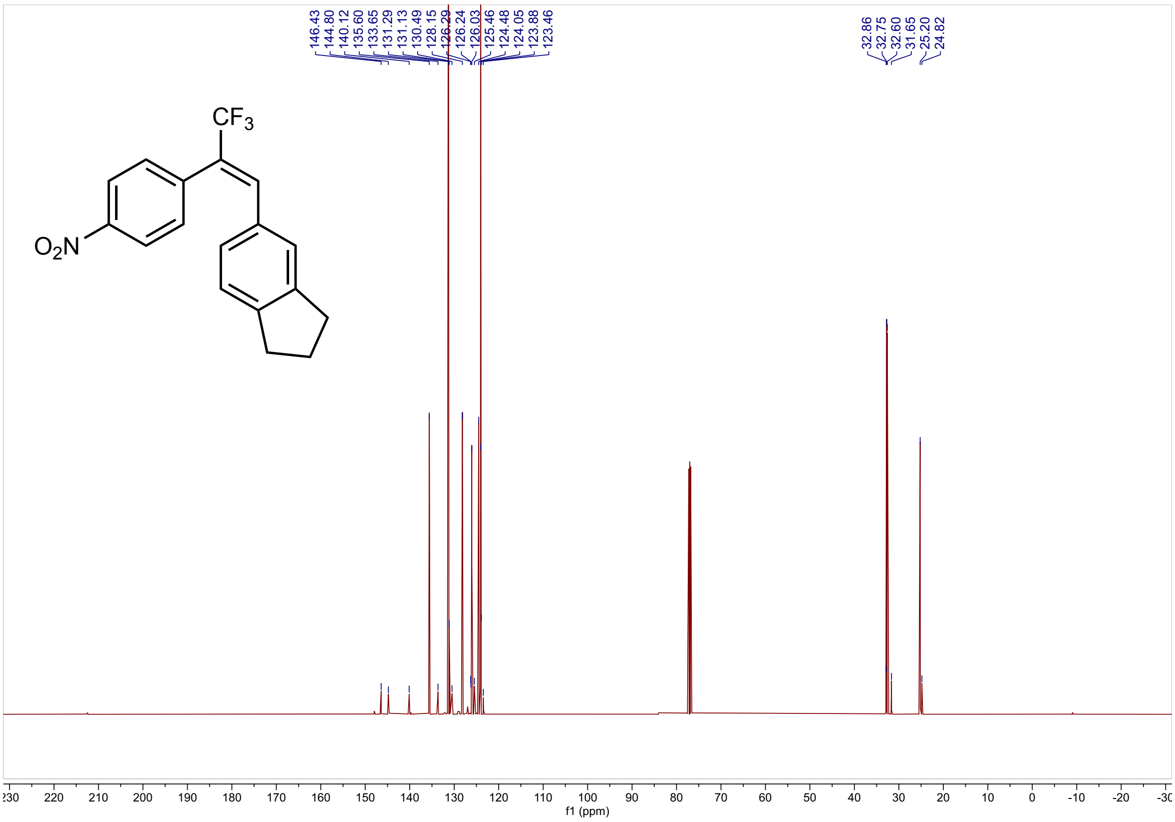

(*E*)-1-Methoxy-4-(3,3,3-trifluoro-2-(4-nitrophenyl)prop-1-en-1-yl)benzene (12i)

^{1}H NMR (600 MHz, Chloroform-*d*)

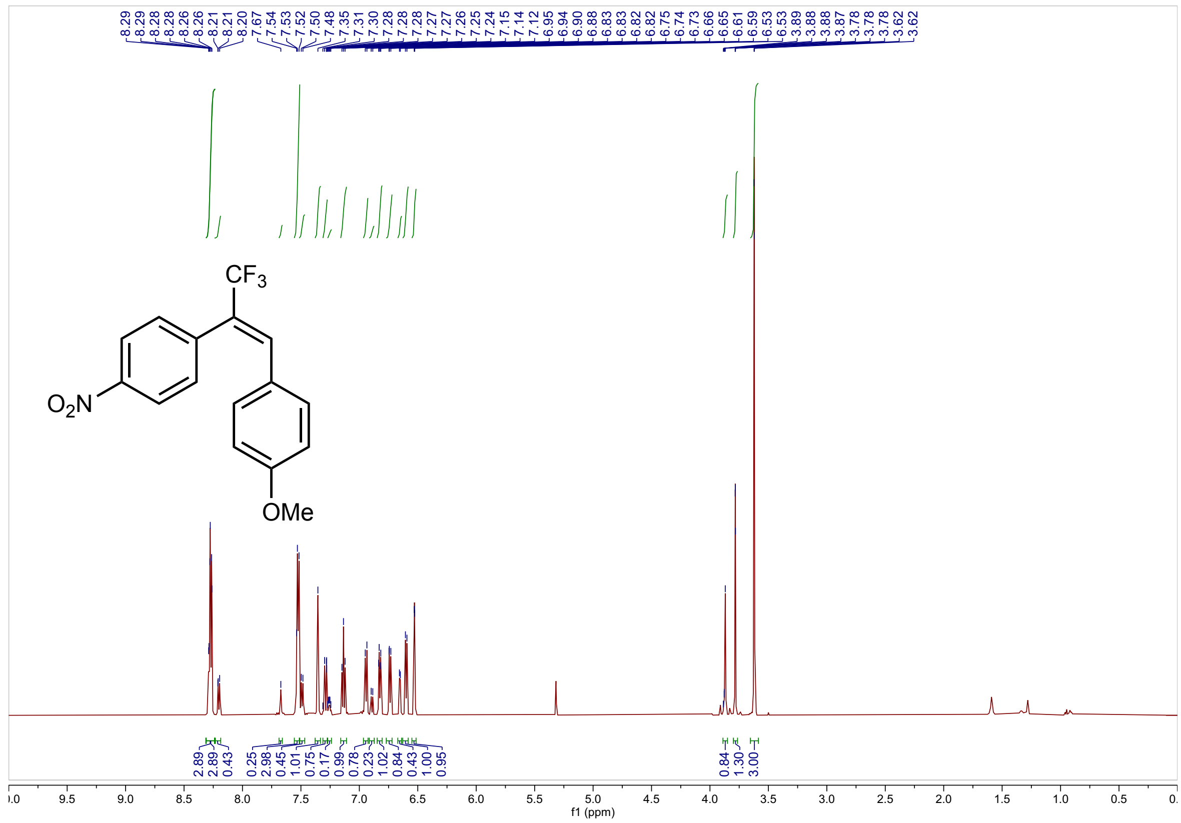

^{19}F NMR (565 MHz, Chloroform-*d*)

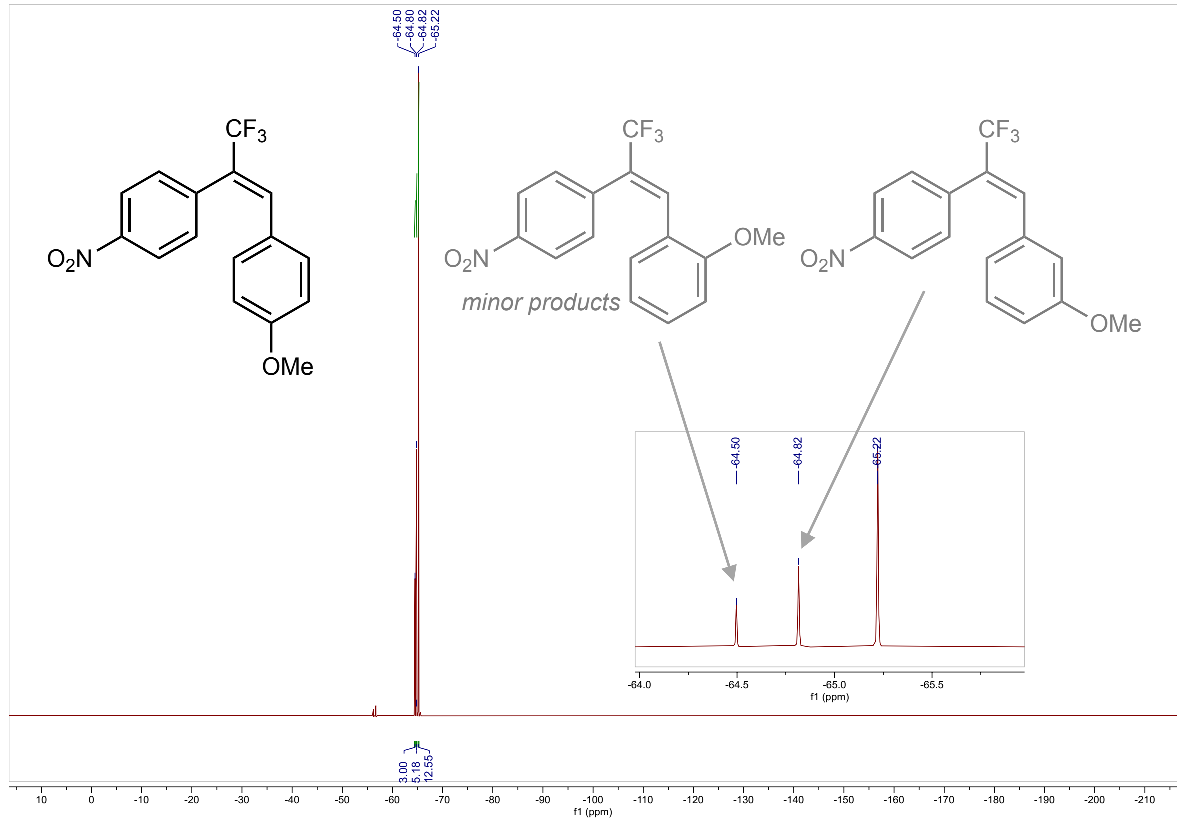

^{13}C NMR{^{1}H, ^{19}F} (151 MHz, Chloroform-*d*)

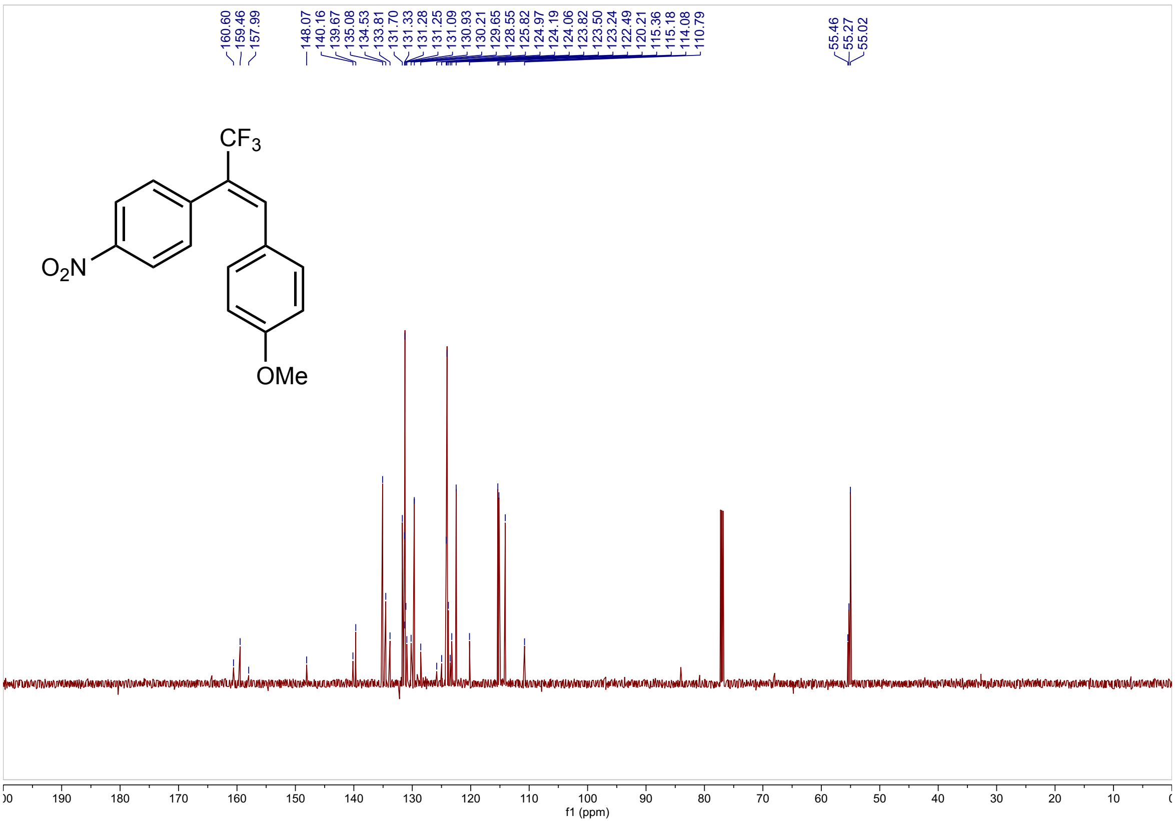

(*E*)-1-(3-(3,3,3-trifluoro-2-(4-nitrophenyl)prop-1-en-1-yl)phenyl)ethan-1-one (12j)

^{1}H NMR (600 MHz, Chloroform-*d*)

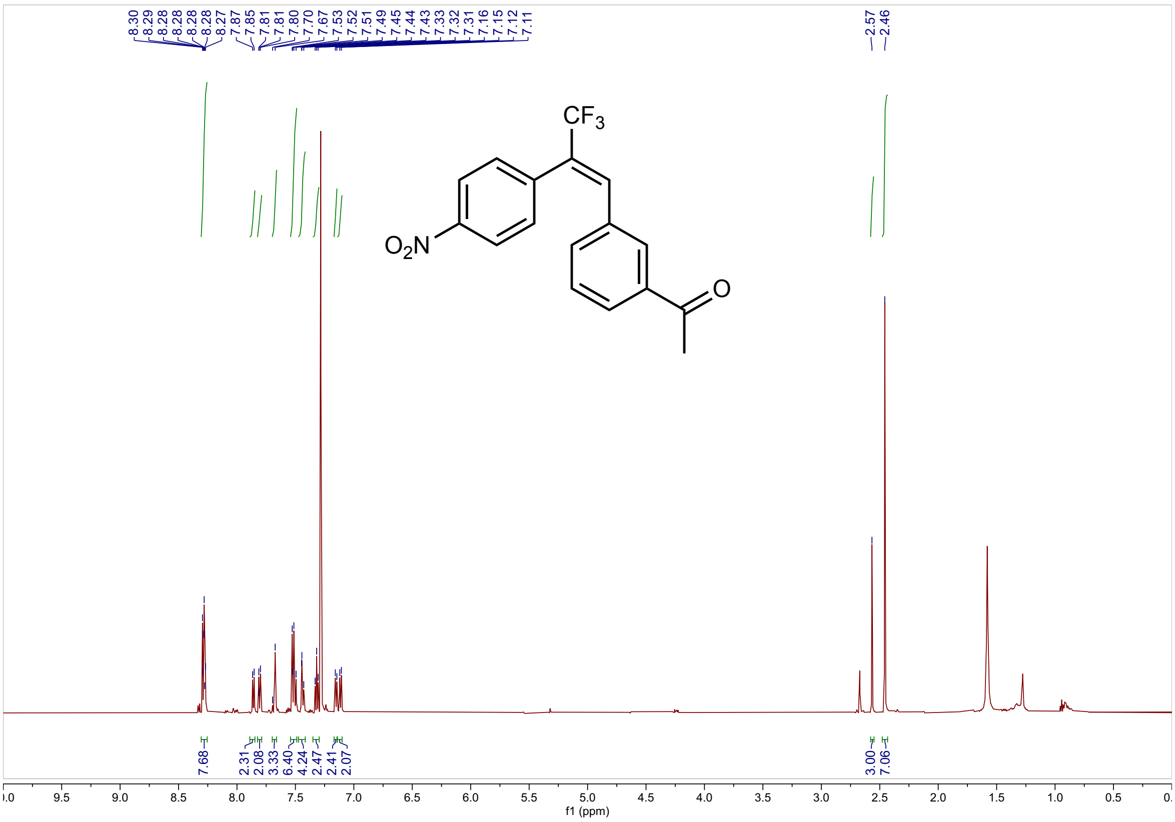

^{19}F NMR (565 MHz, Chloroform-*d*)

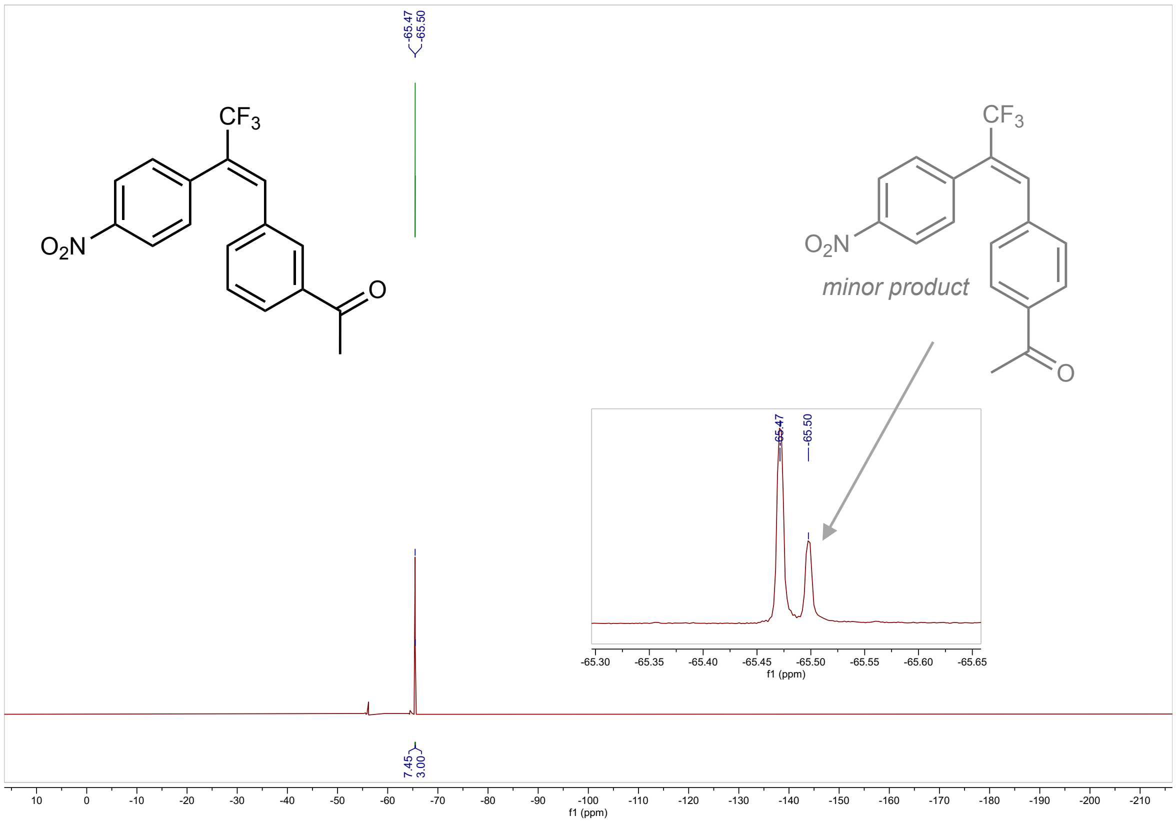

^{13}C NMR{^{1}H, ^{19}F} (151 MHz, Chloroform-*d*)

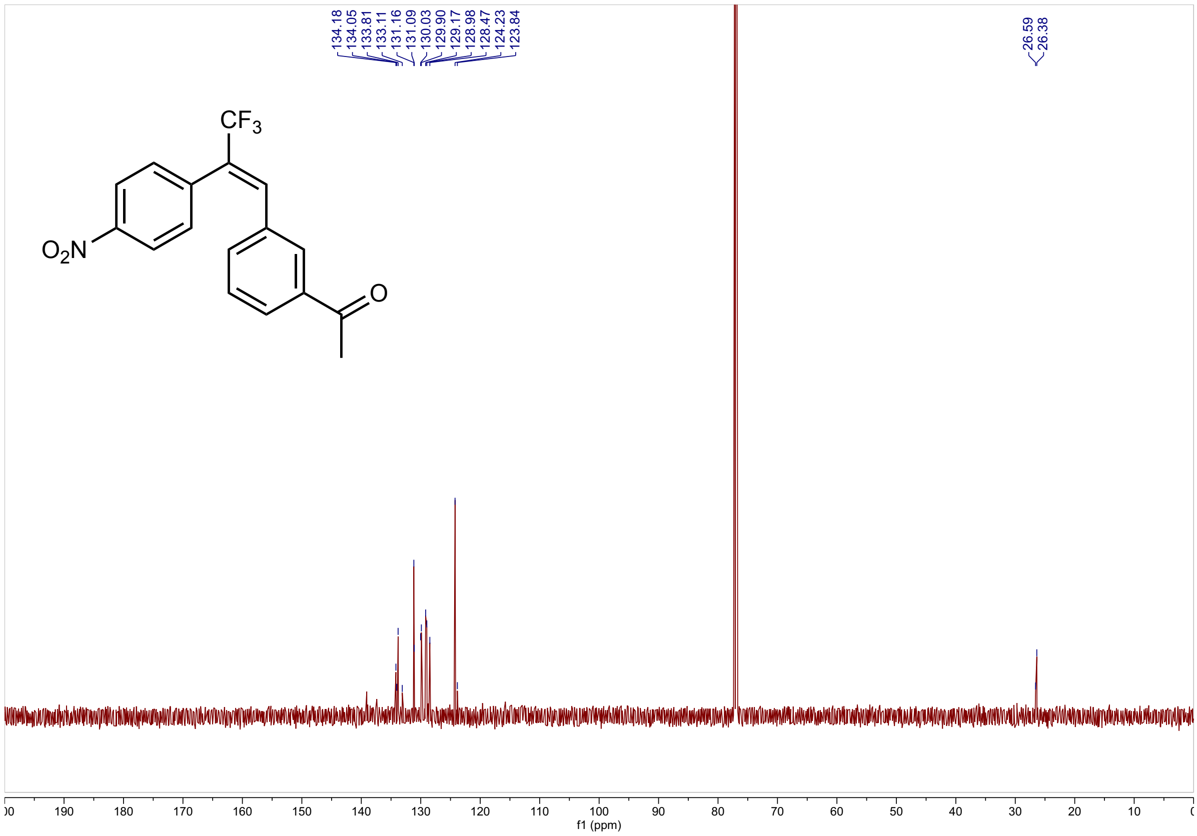

Methyl (*E*)-3-(3,3,3-trifluoro-2-(4-nitrophenyl)prop-1-en-1-yl)benzoate (12k)

^{1}H NMR (600 MHz, Chloroform-*d*)

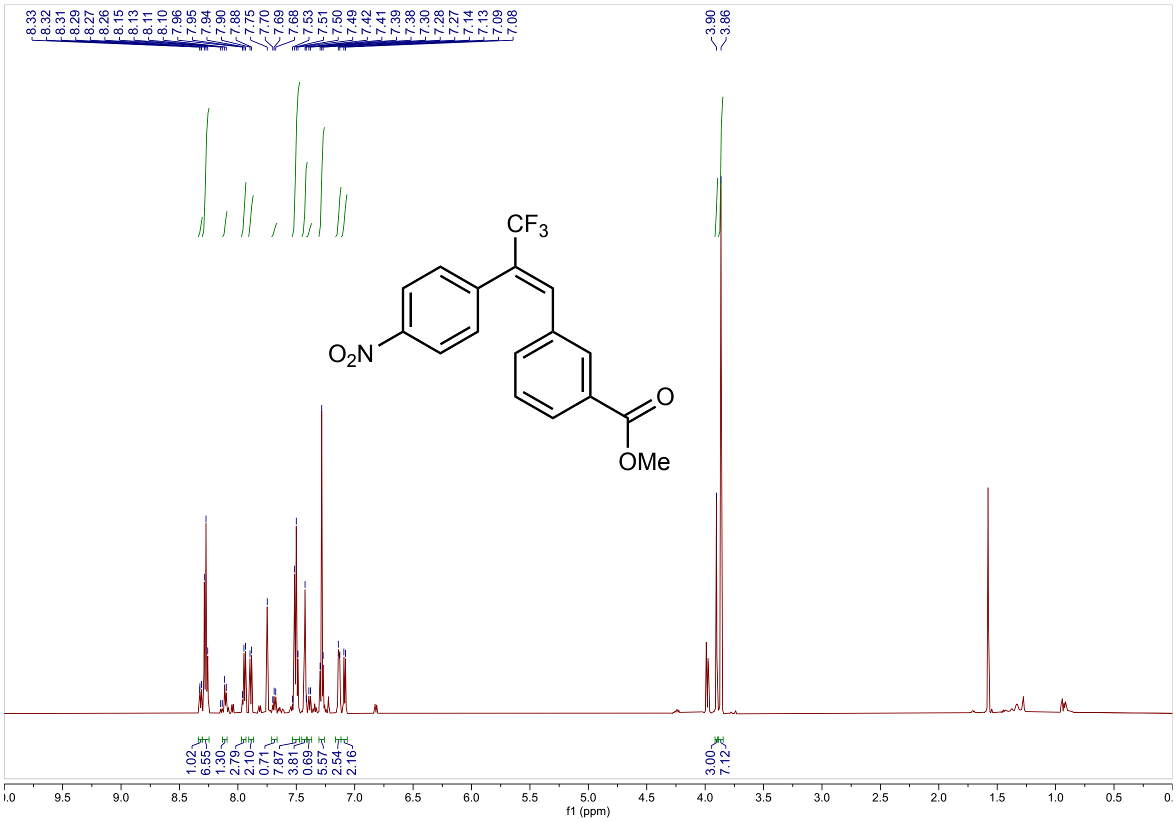

^{19}F NMR (565 MHz, Chloroform-*d*)

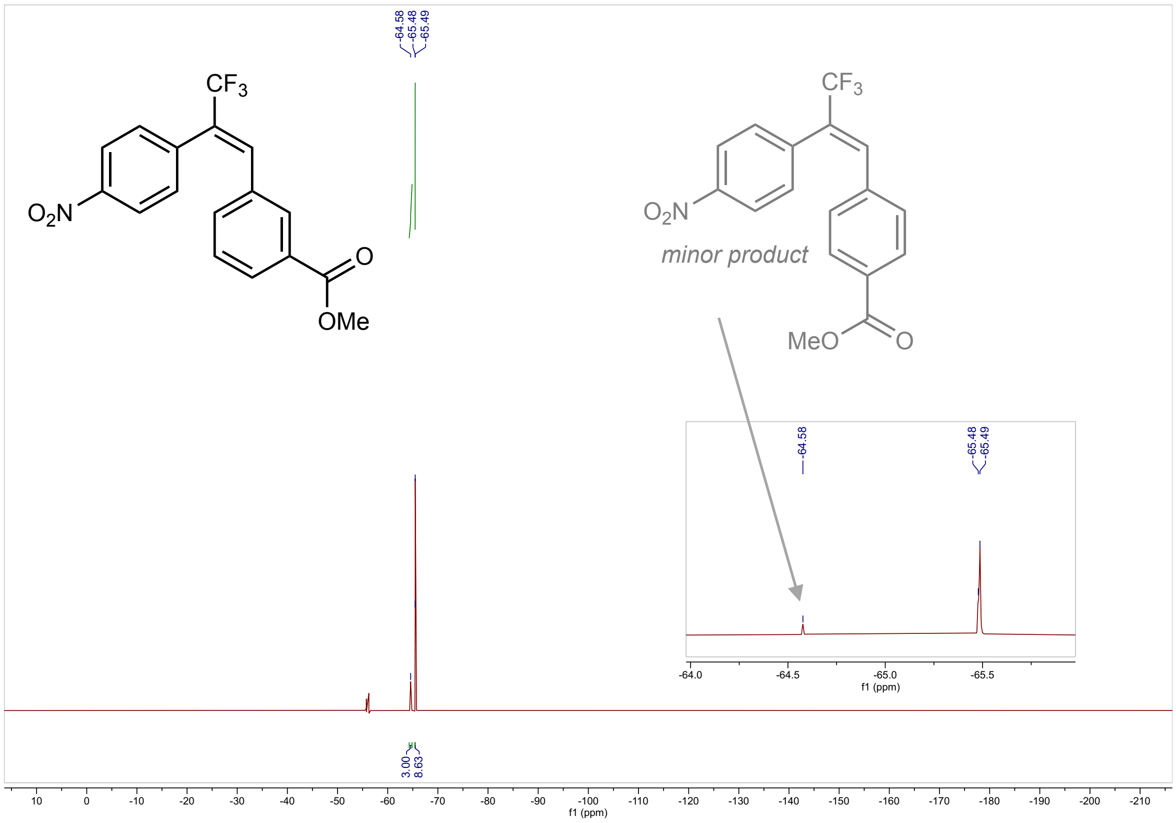

^{13}C NMR{^{1}H, ^{19}F} (151 MHz, Chloroform-*d*)

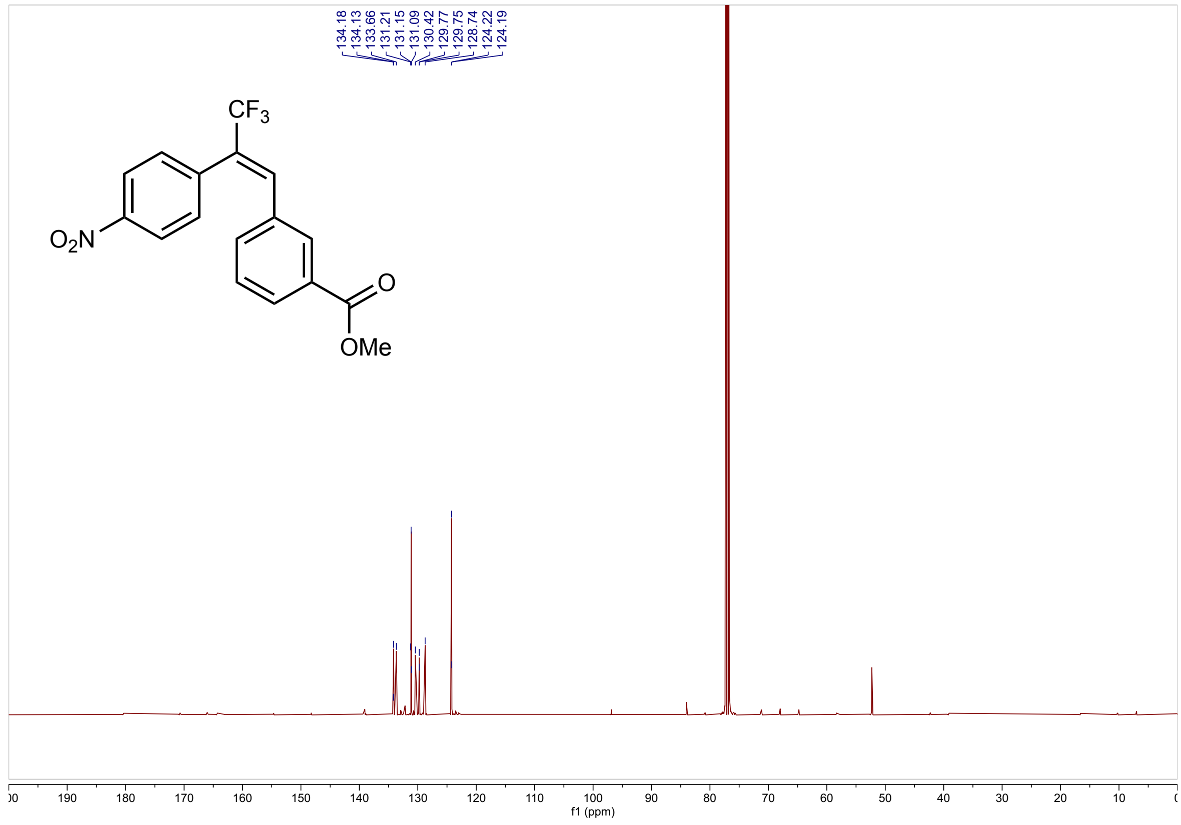

8. References

[16] X. Wang, Y. Xu, Y. Deng, Y. Zhou, J. Feng, G. Ji, Y. Zhang, Y. *Chem. Eur. J.* **2014**, *20*, 961-965.

[17] M. Leiendecker, C.-C. Hsiao, L. Guo, N. Alandini, M. Rueping, *Angew. Chem. Int. Ed.* **2014**, *53*, 12912-12915.

[21] T. Konno, J. Chae, T. Tanaka, T. Ishihara, H. Yamanaka, *J. Fluorine Chem.* **2006**, *127,* 36-43.
